# Explaining human interactions on the road by large-scale integration of computational psychological theory

**DOI:** 10.1093/pnasnexus/pgad163

**Published:** 2023-06-20

**Authors:** Gustav Markkula, Yi-Shin Lin, Aravinda Ramakrishnan Srinivasan, Jac Billington, Matteo Leonetti, Amir Hossein Kalantari, Yue Yang, Yee Mun Lee, Ruth Madigan, Natasha Merat

**Affiliations:** Institute for Transport Studies, University of Leeds, LS2 9JT Leeds, UK; School of Psychology, University of Leeds, LS2 9JT Leeds, UK; Institute for Transport Studies, University of Leeds, LS2 9JT Leeds, UK; Institute for Transport Studies, University of Leeds, LS2 9JT Leeds, UK; School of Psychology, University of Leeds, LS2 9JT Leeds, UK; Department of Informatics, King’s College London, WC2B 4BG London, UK; Institute for Transport Studies, University of Leeds, LS2 9JT Leeds, UK; Institute for Transport Studies, University of Leeds, LS2 9JT Leeds, UK; Institute for Transport Studies, University of Leeds, LS2 9JT Leeds, UK; Institute for Transport Studies, University of Leeds, LS2 9JT Leeds, UK; Institute for Transport Studies, University of Leeds, LS2 9JT Leeds, UK

## Abstract

When humans share space in road traffic, as drivers or as vulnerable road users, they draw on their full range of communicative and interactive capabilities. Much remains unknown about these behaviors, but they need to be captured in models if automated vehicles are to coexist successfully with human road users. Empirical studies of human road user behavior implicate a large number of underlying cognitive mechanisms, which taken together are well beyond the scope of existing computational models. Here, we note that for all of these putative mechanisms, computational theories exist in different subdisciplines of psychology, for more constrained tasks. We demonstrate how these separate theories can be generalized from abstract laboratory paradigms and integrated into a computational framework for modeling human road user interaction, combining Bayesian perception, a theory of mind regarding others’ intentions, behavioral game theory, long-term valuation of action alternatives, and evidence accumulation decision-making. We show that a model with these assumptions—but not simpler versions of the same model—can account for a number of previously unexplained phenomena in naturalistic driver–pedestrian road-crossing interactions, and successfully predicts interaction outcomes in an unseen data set. Our modeling results contribute to demonstrating the real-world value of the theories from which we draw, and address calls in psychology for cumulative theory-building, presenting human road use as a suitable setting for work of this nature. Our findings also underscore the formidable complexity of human interaction in road traffic, with strong implications for the requirements to set on development and testing of vehicle automation.

**Significance Statement**

Before automated vehicles can be deployed in highly interactive traffic environments, they need to be capable of taking part in these interactions in a safe and human-acceptable manner. However, this requires quantitative models of how humans interact, communicate, and understand each other. We demonstrate that several phenomena of this nature in driver–pedestrian interaction can be explained by adopting a range of existing but previously separate mathematical theories in psychology, and integrating these into one model. This is useful because it provides a concrete demonstration of integration of psychological theory, increases our understanding of human road user interaction, and demonstrates the high complexity underlying the human behaviors with which automated vehicles will need to coexist.

## Introduction

A large share of our daily interactions with other humans in society happen while moving on or near roads, as drivers, pedestrians, cyclists, and so on. The empirical literature suggests that interactions between road users depend on much of the same complex underlying cognitive machinery as other forms of human interaction, including decision-making mechanisms (1), speed-accuracy tradeoffs between goal-achieving and risk-taking (2), game-theoretic reasoning (3), a theory of mind to estimate the intentions of others (4) and how those intentions are influenced by one’s own actions (5), such as implicit and explicit communication (6, 7). However, no integrated theory or model exists which combines these putative cognitive mechanisms to more comprehensively explain and predict road user interaction behavior.

In recent years, there has been a push toward introducing automated vehicles on public roads, but safe and human-acceptable deployment of these vehicles into more complex, interactive environments currently remains hampered by a lack of models of how human road users interact. Such models are needed both in real-time algorithms to predict human behavior (8, 9) and for simulated testing with virtual human agents (10, 11). This need has prompted a surge of research developing road user interaction models. Most of these models have been application-oriented and emphasize high-level metrics of average deviation between observed and predicted trajectories, such as root mean square error (8–11), rather than engaging with the specific behavioral phenomena and putative underlying cognitive mechanisms implicated by the empirical literature. Other models take this more cognitive–behavioral perspective, but limit the scope to one or a few mechanisms at a time (12–15). This leaves the question open: How complex is the cognitive machinery underlying human interaction on the road, and what will it take to capture the resulting behavior in models?

At the same time, it should be noted that there are entire subfields of psychology focusing exclusively on each of the cognitive mechanisms listed above, with computational cognitive models developed to account for behavior in abstract tasks in the laboratory (16–24). Some authors find this somewhat fragmented state of psychological theory limiting, and there is a recurring argument for more cumulative theory-building, where one possible way forward is integration of separate theories into larger models, to describe human behavior in more complex, real-world tasks (25, 26). Here, we take on this challenge by developing a psychological model of road user interaction, focusing on interactions between drivers and pedestrians, which is a particularly difficult scenario for automated vehicles (8). Drawing from theoretical work on model selection in psychology (27), we diverge from previous road user interaction modeling work by starting not from a data set to be fitted, but instead from a set of behavioral phenomena we wish to account for. We adopt mathematical formulations from existing computational psychological theories about perception, cognition, action, and interaction, and integrate these into a single, modular framework, allowing us to investigate what theoretical assumptions are required to reproduce what behavioral phenomena.

**Fig. 1.**
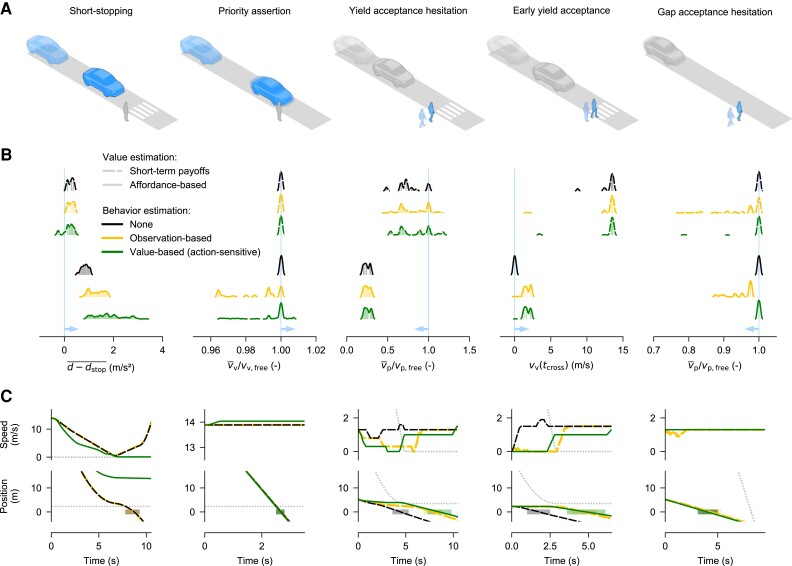
Behavioral phenomena and deterministic model results. A) The targeted phenomena, with the modeled agent in blue (the driver in the first two columns, and the pedestrian in the other three). B) Distributions of behavior metrics obtained across the parameter space of six selected deterministic model variants (explained further in the text; see also Online Supplementary Fig. S1). The light blue vertical lines and arrows indicate the region where each phenomenon is increasingly clearly exhibited. C) Example time-series simulations for three of the models in B. Gray dotted lines show behavior of the nonmodeled agent; shaded rectangles indicate when agents are in the contested road space.

Fig. 1A shows the five empirically well-established driver–pedestrian interaction phenomena we use as our primary targets for modeling, including different forms of apparent implicit communication (*priority assertion*: drivers speeding up when denying priority to pedestrians (5, 28); *short-stopping*: drivers exaggerating deceleration to encourage pedestrians to cross (6, 29)), hesitation (*yield acceptance hesitation* and *gap acceptance hesitation*: pedestrians slowing down to delay their crossing in front of both yielding and nonyielding cars (1, 2, 6)), and interpretation of others’ intentions (*early yield acceptance*: pedestrians beginning to cross before a yielding vehicle has come to a full stop (1, 6)). These phenomena were chosen here for having been hypothesized to draw from a wide range of underlying cognitive mechanisms (1–6), and similar phenomena are present also in interactions involving other types of road users, e.g. driver–driver interactions (30, 31).

**Fig. 2.**
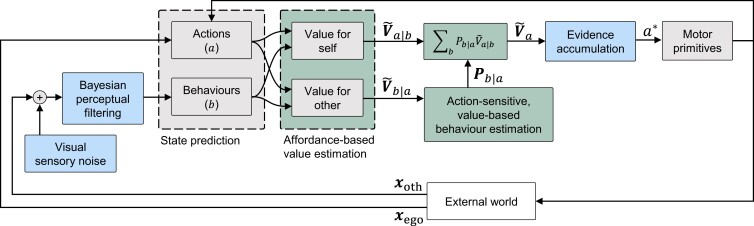
Maximally successful model variant. Colors indicate assumptions included in the base model (gray) and selected in the deterministic (green) and stochastic (blue) model tests. See Online Supplementary Fig. S1 for an illustration of the full model framework from which this specific model variant is derived.

Online Supplementary Fig. S1 shows the perception–action loop of the full model framework we developed to account for the targeted empirical phenomena, and Fig. 2 shows the maximally successful model variant we identified within this framework (the lowest-complexity model explaining the largest number of phenomena). Two framework assumptions (shown in gray in Fig. 2) are shared among all our tested model variants: First, based on theories of motor primitives (32) and intermittent sensorimotor control (33), which have been shown to explain driver behavior in both routine and near-crash situations (34, 35), and based on observations and models of stepwise adjustments to pedestrian walking speed (2, 36, 37, 38), we model the longitudinal locomotion of driver and pedestrian as constructed from intermittent adjustments to acceleration and speed, respectively. Second, aligning with a long modeling tradition in psychology and neuroscience (21), we assume that agents decide what motor primitives to apply by estimating the value (or utility, or predicted reward) from applying each alternative action.

In the following, we extend on this base model with different combinations of assumptions from other psychological theories. For each tested model variant, we comprehensively searched a space of plausible model parameterizations, simulated each model parameterization across kinematical variants of the targeted scenarios, and quantified, using various behavior metrics, whether the model exhibited the phenomena in Fig. 1A. This model selection process first considered fully deterministic model variants only, thereafter expanding the selection to also include stochastic model assumptions. We then narrowed down our model selection further using data from a controlled experiment, and performed a validation test of the final model by comparing its predictions to data from a second experiment. Below, our main findings will be presented; details about the model, model selection metrics, data sets, and tests are presented in the Methods section, and full results across all tested model variants are provided in the Online Supplementary Material.

## Results

### Short-term value estimation captures only basic collision aversion

Fig. 1B shows, for six selected deterministic model variants, distributions of observed behavior metrics across each model’s entire tested parameter space, thus showing the range of behaviors each model is capable of expressing. Fig. 1C shows example time-series simulations for selected model parameterizations.

In game-theoretic models of human interaction (3, 18), value is often formulated as a single payoff obtained after a one-shot interaction (a single, simultaneous decision by all players). We first formulated value estimation as this type of payoff, a short-term (0.5 s) prediction into the future from each time step for each possible movement adjustment, with positive reward from making locomotor progress, a cost for being on a collision course, discomfort/effort costs from speed and accelerations, and a cost for violating priority rules. We found that this base model variant (black dashed line in Fig. 1B and C) could account for two of our targeted phenomena, namely yield acceptance hesitation (the pedestrian slows down before beginning to cross in front of the yielding vehicle; ${\bar{v}}_{p}/v_{p,\mathrm{free}}<1$ in Fig. 1B for many model parameterizations) and early yield acceptance (the pedestrian begins crossing before the yielding car has come to a full stop; $v_{v}(t_{\mathrm{cross}})>0$ in Fig. 1B), although with a tendency for very early yield acceptance (high $v_{v}(t_{\mathrm{cross}})$ values).

However, this simple model was not able to account for the other three targeted phenomena: short-stopping (as can be seen in Fig. 1C, the slightly positive average excess deceleration $\bar{d-d_{\mathrm{stop}}}$ in Fig. 1B for this model does not translate to stopping short; see further Online Supplementary Section 2.1 and Figs. S5 and S6), priority assertion (no ${\bar{v}}_{v}/v_{v,\mathrm{free}}>1$), or gap acceptance hesitation (no ${\bar{v}}_{p}/v_{p,\mathrm{free}}<1$).

### Extending short-term value estimation with a theory of mind is not enough

It has been hypothesized that phenomena like short-stopping, priority assertion, and hesitation arise because human road users have a theory of mind about each other (19, 39), in the specific sense that they reason about each other’s intentions during interactions (1, 4, 6). Separate psychological theories exist for how humans infer others’ intentions by considering the situation at hand from the other’s perspective and either observing their actions (19, 20) (referred to here as observation-based behavior estimation), or judging what behavior would be rational—i.e. value-maximizing—for the other agent (18, 22) (value-based behavior estimation), in the latter case sometimes also considering the impact of one’s own actions on the other (12) (action-sensitive, value-based behavior estimation). We show in the Methods section how several such theories can be combined into a joint, modular model of behavior estimation, capable of considering both the actual observed movement of the other agent, and what would be rational of the other agent, with or without consideration of the impact of one’s own actions. Since the possible outcomes of a (collision-free) interaction is always that one or the other agent passes the crossing location first, we assume that the agents are estimating the behaviors of the other agent in terms of probabilities of these two intended access orders. We extended the base model with all possible variants of this behavior estimation model, and found that some of the resulting model variants were capable of exhibiting gap acceptance hesitation (yellow and green dashed lines in Fig. 1B and C; ${\bar{v}}_{p}/v_{p,\mathrm{free}}<1$), but there were still no parameterizations for which these model variants were capable of priority assertion or short-stopping (Fig. 1B and C, Online Supplementary Fig. S5; again, as can be seen for the yellow dashed line in Fig. 1C, the slightly positive excess deceleration $\bar{d-d_{\mathrm{stop}}}$ in Fig. 1B did not translate to the model actually stopping short).

**Fig. 3.**
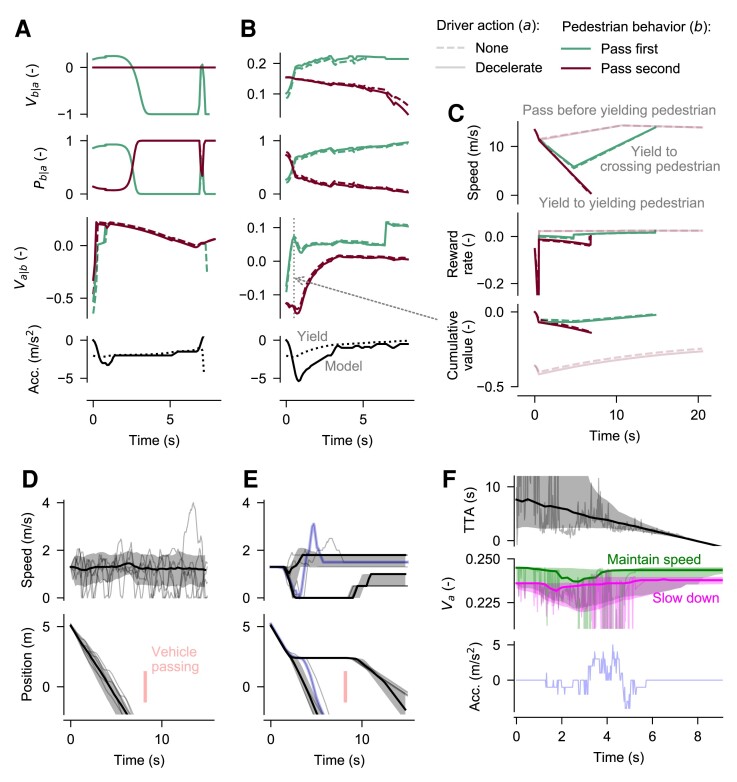
Model behavior in the driver short-stopping (panels A–C) and pedestrian gap acceptance hesitation (panels D–F) scenarios. A) Short-term payoff model with action-sensitive value-based behavior estimation, unable to exhibit short-stopping. B) Affordance-based model with action-sensitive value-based behavior estimation, exhibiting short-stopping. C) Detailed view of the affordance-based value estimation (from t=0.5 s in B). D) Model with value noise, not exhibiting gap acceptance hesitation. Thin lines show example simulations; thick lines and shaded areas show median and 20–80 percentiles across 500 different model parameterizations. E) As in panel E, but for a model with sensory noise, exhibiting gap acceptance hesitation. F) Internal model states for the simulations in E; thin lines are for the example simulation highlighted in blue in E.

Fig. 3A illustrates this limitation, in an example simulation of the driver agent model, with action-sensitive, value-based behavior estimation (i.e. the driver assumes that the pedestrian will behave rationally, to maximize own reward, in response to the driver’s actions) in a scenario with pedestrian priority, where as mentioned human drivers will often exaggerate deceleration and short-stop. In this simulation, the driver agent never judges that the own action *a* will affect the behavior *b* of the pedestrian (there is complete overlap between the dashed and solid lines for value $V_{b|a}$ and probability $P_{b|a}$ of pedestrian behavior *b* given own action *a*). The reason for this weak coupling between own actions and behavior of the other is that the short-term payoff model has a highly constrained view of future events. This prevents the driver from seeing a benefit of increasing deceleration (black solid line in bottom panel of Fig. 3A) beyond the minimum required to yield to the pedestrian (black dotted line).

### Implicit communication from theory of mind and long-term value estimation

In contrast to the short-term payoff assumption, in models of reward-driven behavior, it is often assumed that humans maximize value over a longer prediction horizon (40), sometimes also in interactive, game-theoretic settings (12, 13). We tested a second value estimation formulation which evaluates, for each possible motor primitive, the long-term value for the agent of passing before or after the other agent, after having applied the motor primitive in question. We used the same rewards and costs as for the short-term payoff model, but now integrated these into the agent’s anticipated future, with exponential temporal discounting, i.e. a preference for earlier rewards (40). The obtained values quantify simultaneously how desirable and available the two possible access orders are for the agent, or differently put the agent’s expected *affordances* for the two access orders (41, 42), after applying a given motor primitive.

This affordance-based model, combined with action-sensitive, value-based behavior estimation, was capable of exhibiting not only yield acceptance hesitation and early yield acceptance but also short-stopping (Fig. 1B; $\bar{d-d_{\mathrm{stop}}}>0$, leading to a substantial final stopping distance as seen in Fig. 1C) and (modest) priority assertion (${\bar{v}}_{v}/v_{v,\mathrm{free}}>1$). Fig. 3B shows an example simulation of this model in the same scenario as in Fig. 3A. It can be seen that the highest-value outcome for the affordance-based driver would consistently be that the pedestrian crosses first (green $V_{a|b}$ lines higher than red), and from about t≥0.5 s, at which point the driver has reached a sufficient deceleration for yielding, the driver would prefer that the pedestrian crossed first without the driver having to decelerate further (dashed green $V_{a|b}$ highest). To illustrate the calculations underlying this preference, Fig. 3C provides a snapshot of the affordance-based value estimation of $V_{a|b}$ at time t=0.5 s, showing how the driver agent envisions the different possible future outcomes from this point, in terms of own future speeds, and associated momentary and cumulative reward. Using a similar evaluation of future values for the pedestrian, the driver agent can now also estimate that the value, and therefore probability, for the pedestrian of actually beginning to cross in front of the car is higher if the driver does increase deceleration further (solid green $V_{b|a}$ and $P_{b|a}$ higher than dashed green lines). Thus, on balance, since the value of yielding to a yielding pedestrian is low (dark red lines in Fig. 3B and C), the driver chooses to further increase deceleration, resulting in short-stopping.

### Value-transformed sensory noise explains gap acceptance hesitation

As shown earlier, gap acceptance hesitation can arise in these deterministic models due to uncertainty about the other agent’s behavior, but this was not the case in any of the model variants capable of exhibiting the other four targeted phenomena. Thus, our maximally successful deterministic model (gray and green boxes in Fig. 2; solid green lines in Fig. 1B and C) leaves gap acceptance hesitation unexplained. There are many theories of perception and cognition which instead describe uncertainty as arising from nondeterministic processes, and we integrated two dominant such theories into our framework: Bayes-optimal interpretation of noisy sensory input (23, 24) and accumulation of noisy evidence in favor of an action (e.g. noisy action value) to a threshold before committing to a decision (16, 17). In both of these theories, noise is added at model input, as sensory noise and value noise, respectively. However, we separated out the assumptions about where noise is injected, to create a larger set of different models combining the two theories.

We found that the presence of sensory noise, but not value noise, caused the model to exhibit gap acceptance hesitation. Fig. 3D shows that adding symmetric value noise at the pedestrian’s decision-making stage (16, 17) introduces similarly symmetric variations in pedestrian walking speed, with no clear influence of the approaching car, and no bias for slowing down. In contrast, the simulations with sensory noise in Fig. 3E show a distinct onset of pedestrian deceleration, either to a full stop to wait for the car to pass, or followed by an acceleration to cross before the car. The reason for this model behavior can be seen in Fig. 3F: The sensory noise causes a symmetric uncertainty about, for example, the vehicle’s time to arrival (TTA), but since the consequences for the pedestrian of a lower rather than a higher TTA are highly asymmetric, the model’s value estimation transforms the symmetric sensory noise into value noise that is asymmetric, skewing more towards low values $V_{a}$ (the shaded areas indicating 20–80 percentiles of $V_{a}$ extend much further below than above the medians). This skew is particularly large for the action of maintaining initial speed to cross in front of the car, resulting in a risk-averse preference for slowing down, i.e. gap acceptance hesitation. It can be noted that the same risk aversion causes those pedestrians who do cross in front of the car to do so at an elevated walking speed, even though this is objectively unnecessary; this type of elevated road-crossing speed is also well known in human pedestrians (2, 43).

### Model with Bayesian perception and evidence accumulation predicts empirical data

**Fig. 4.**
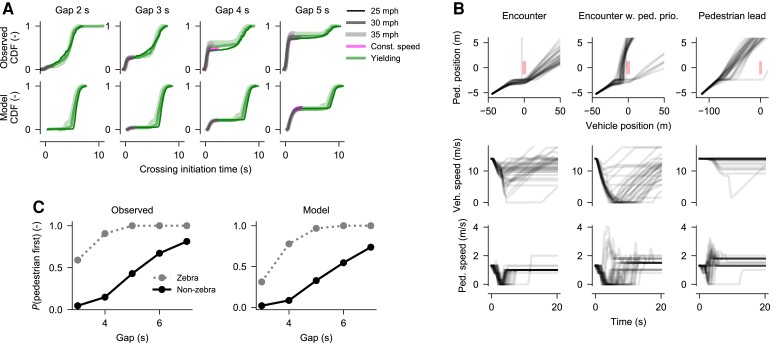
Predictions by the maximally successful model, and comparisons to controlled experiment data. A) Observed and model-predicted crossing initiation times in Experiment 1. B) Example of two-agent model simulations. The red rectangular areas in the top panels indicate positions in which the agents would be colliding. C) Observed and model-predicted interaction outcomes in Experiment 2.

We next combined the deterministic and stochastic model variants that were successful in the tests described above, generating a number of more complex model variants, with populations of parameterizations sampled from those that had been previously successful in the separate deterministic and stochastic tests. We subjected the resulting models to further tests. First, we used a data set from a high-fidelity pedestrian simulator experiment, where 60 participants in a CAVE virtual reality environment decided if and when to cross in gaps between approaching, constant-speed vehicles. The resulting data set of 7,200 trials showed nontrivial distributions of crossing onset time (Fig. 4A), with an early mode of crossing, of mass depending on gap size, and in the deceleration trials also a second mode of crossing as the yielding vehicle was approaching zero speed. We generated population-level predictions for these experimental conditions from all of our more complex model variants, and found that only those model variants which included both Bayesian perceptual filtering and evidence accumulation predicted the bimodal crossing pattern of the humans (Fig. 4A; see further Online Supplementary Fig. S18). We consider the simplest of these model variants our maximally successful model (Fig. 2).

Next, we tested this maximally successful model in interactive simulations, where both driver and pedestrian were controlled by the model (Fig. 4B), and found that the model was capable of collision-free two-agent interaction. Beyond the main phenomena targeted in this paper it also exhibited one additional phenomenon: In time-symmetric vehicle–pedestrian encounters without clear pedestrian priority (leftmost panels in Fig. 4B), vehicles almost always end up passing first (5).

Finally, we conducted a controlled experiment where 32 pairs of human participants interacted as driver and pedestrian, in a high-fidelity, distributed driver–pedestrian simulator. We varied the presence of a zebra crossing indicating pedestrian priority, and cued the pedestrian participant to step up to the curb from behind a vision obstruction when the driver participant was at different time gaps from the crossing, thus varying the initial kinematics of the interaction. As can be seen in Fig. 4C, in the obtained data set of 1,280 human–human interactions, the outcome in terms of who passed the crossing first varied with both initial kinematics and priority rule, and we found that two-agent simulations of our model in these experimental conditions predicted these dependencies well (Fig. 4C).

It is worth emphasizing that Fig. 4 does not show fits of the model to either of the two considered data sets; simply predictions from the model after it has been constrained to exhibit our other targeted phenomena, and after excluding model parameterizations with a tendency to “get stuck.”

## Discussion

We have presented a framework for modeling road user interactions, demonstrating how a range of existing computational psychological theories fit together mathematically, and can be combined to create joint models integrating these theories. Our framework describes behavior as shaped by long-term reward, behavioral game theory, sensory noise, Bayesian perception, a theory of mind regarding the intentions of others, and evidence accumulation decision-making. Our main finding is that only a model which includes all of these assumptions can account for all of our five targeted phenomena, observed in naturalistic driver–pedestrian interaction (Fig. 1A). Previous models have accounted for early yield acceptance (1) and short-stopping (44) separately; our maximally successful model accounts for both, and our other three targeted phenomena as well. Furthermore, we found that this model, without further parameter-fitting, reproduced two additional phenomena (pedestrian crossing speed-up and driver advantage in symmetric vehicle–pedestrian encounters), and successfully predicted behavior patterns observed in two controlled studies. We draw three main conclusions from our findings.

The first main conclusion is quite simply that a comprehensive understanding and modeling of human road user interaction seems likely to require substantial integration of psychological theory. As described in the Introduction section, this hypothesis has previously been indirectly discernible across the empirical literature on road user behavior. Here, we explicitly formulated and tested it, and found clear support in its favor, by means of one of the largest-scale integrations of psychological theory of which we are aware. The fact that this integration was done in the context of road user interaction is meaningful also in the sense that it provides a concrete demonstration of how the existing theories, often tested mostly in abstract laboratory tasks, can be put to use in a real-world context with high applied relevance.

Following on from the above, our second main conclusion is that constructing a task-specific model of a complex real-world task can indeed be a fruitful approach to cumulative theory-building in psychology, as has been suggested by some (25, 26). A more common approach to psychological modeling of real-world tasks has been to first pursue task-general cognitive architectures (45, 46), and then build on these to model specific tasks, for example in driving (47). However, few or none of the model assumptions we have found useful here come “for free” in these architectures, so adopting these assumptions into an existing architecture would have resulted in an even more complex model than the one we have presented here. This type of combined approach could also be useful, for example to address modeling challenges relating to road user distraction, for which ACT-R has been previously used (48). A benefit of the task-specific approach to theoretical integration that we took here was that it allowed us to focus specifically on how the existing theories fit together mathematically, opening up new possible lines of psychological modeling research on these combined models. Most notably, our joint formulation of combined observation-based and value-based behavior estimation, as well as our approach of modeling noise in decision variables as value-transformed sensory noise, could merit targeted further investigation.

It is clear that there is still much room for future improvement of our model. For example, its priority assertion (Figs. 1B and C, 4B) is quite modest compared to what has been observed in human drivers (5). It is possible that this could be improved upon by minor adjustments to our current model formulations. Another possibility is that human driver acceleration in these scenarios is determined not only by a desire for own progress but also for minimizing waiting time for the pedestrian; this could be accounted for by extending our model framework to incorporate a theory such as social value orientation (13, 49). Another limitation of our most successful model is that some of the parameterizations retained from the model selection tests showed a tendency to remain stationary at zero speed in some interactions. We discuss in the Online Supplementary Section 2.3 how one main reason for this behavior is the specific formulation for evidence accumulation we adopted here; the large literature on evidence accumulation decision-making suggests a range of more general alternatives which could be tested (16, 17, 50, 51). Overall, disciplinary experts in the various subfields of psychological modeling from which we are drawing may find that parts of our framework ought to be formulated differently. This can suggest valuable avenues for further improvements to our model, with one question of specific interest being whether some alternative model formulations lend themselves better than others to integration within a larger theoretical construction, such as the one we have developed here.

A more general limitation of any test of a theory is that some entirely different theory or set of theories might explain the same data just as well or better. In other words, the results we have presented here do not prove that human road user interaction relies on the exact mechanisms we have posited in our most successful models. For example, the brain may directly estimate value of alternative actions without predicting any future states (52), or may use direct action policies or perceptual-motor heuristics, perhaps without any internal representation at all (53, 54). However, even if so, it seems likely that the rich variety of empirical phenomena addressed in this paper would still require a highly complex set of state-action value mappings, perceptual-motor heuristics, or the like. Indeed, one conclusion drawn by Domeyer et al. (14) from their direct perceptual-motor modeling of driver–pedestrian interaction was that additional model mechanisms seemed necessary to capture what they interpreted as changes of mind during interactions, and decision-making unfolding over time. This perspective is in line with our conclusion here about the need for cross-theoretical integration to model road user interaction, irrespective of what overall modeling framework is adopted. An important line of future work will be to test models based on different frameworks against each other.

Our third and final main conclusion concerns the model complexity resulting from theoretical integration: What we have shown here suggests that modeling of human road user interaction is a formidable challenge. While the structure of our model framework as shown in Fig. 2 may in principle be general enough to address more complex road user interactions, the scenarios and phenomena we have modeled here are still just scratching the surface of interactions occurring in real traffic (31). It is possible that the assumptions in our current model will be sufficient to describe human behavior in a more general range of scenarios, but given the complexity of the model, actually performing this generalization and testing whether it is successful is no small task.

This high model complexity has implications not least in development and testing of vehicle automation. For example, automated vehicles rely heavily on real-time algorithms for recognizing intentions of surrounding road users. However, the rapidly growing literature and data sets on this topic (8, 9, 55) rests largely on the premise that near-term movement of other road users is something that automated vehicles can just passively estimate, rather than something they are intrinsically involved in influencing, as some authors have suggested (12) and as our work here suggests that human road users do. Another important application area is simulated benchmarking and virtual testing of automated vehicles, where scenario-general, realistic models of human road user interaction are needed (8, 10, 11). It is clear from our results that such models are not within easy reach. Mechanistic modeling is approaching its limits here, and a natural alternative is to instead look to data-driven, machine-learned models (10, 11). However, it should be noted that also with respect to this type of modeling, our results highlight the complexity of underlying mechanisms and behavioral phenomena that need to be learned and tested for. There is a mounting argument in favor of thoroughly investigating machine-learned behavior (56), and recent analyses of machine-learned road user models have indeed shown that the standard approach of training these, to minimize deviation between model-predicted and human trajectories, is not guaranteed to yield human-like interaction behavior (57, 58). The mechanistic insights we have presented here, as well our approach of explicitly targeting interaction phenomena to be accounted for, may guide work towards more cognitively and behaviorally informed machine learning, to capture the subtleties of interaction that matter to humans.

## Methods

### Model framework

Below we describe the model update, for each agent, at time step *k* in a discrete-time simulation of the maximally complex model (i.e. including all assumptions tested in this paper; as illustrated in the Online Supplementary Fig. S1). We start from the applied movement control and work backwards through the model. For the sake of brevity, we keep some details to Section 1 of the Online Supplementary Material, which also provides additional explanatory notes, and describes how individual assumptions in the model can be modularly disabled to create lower-complexity model variants. A list of all optional model assumptions is provided in the Online Supplementary Table S1, together with the free model parameters associated with each assumption. Online Supplementary Fig. S2 shows the general geometry of the simulated pedestrian-vehicle interaction scenarios.

#### Movement control by motor primitives

As in reference (34), we assume that the agents construct their sensorimotor control *C* (speed and acceleration, for pedestrian and driver agents, respectively) as a superposition of fixed, stereotyped motor primitives *G* with amplitude $g_{a}$ for action *a* (32):


(1) $$C(k)=\sum_{i=0}^{k-1}g_{a^{*}(k-i)}G(i),$$

where $a^{*}(k)$ is the action chosen at time step *k*, which may often be the action $a_{\emptyset }$ of not adjusting control (i.e. with amplitude $g_{a_{\emptyset }}=0$).

#### Action decisions based on accumulated action value estimates

At each time step, the agent chooses the action $a^{*}$ with the highest accumulated value ${\hat{V}}_{a}(k)$, as long as that accumulated value is more than a threshold $\Delta V_{\mathrm{th}}$ higher than the value of the no-adjustment action $a_{\emptyset }$, i.e.:


(2) $$a^{*}(k)=\left\{ \begin{array}{ll} \underset{a}{argmax}\;{\hat{V}}_{a}(k),\; & \text{if}\;\max_{a}\left({\hat{V}}_{a}(k)-{\hat{V}}_{a_{\emptyset }}(k)\right)>\Delta V_{\mathrm{th}}.\;\\ a_{\emptyset }, & \text{otherwise}. \end{array}\right.$$

The accumulated value of action *a* is calculated using a special case of the more general evidence accumulation schemes studied in the literature (16, 17, 50), effectively a first-order low-pass filter of the noisy action value ${\tilde{V}}_{a}$:


(3) $$\begin{array}{rl} {\hat{V}}_{a}(k) & =A_{T,\sigma_{V}}({\hat{V}}_{a},{\tilde{V}}_{a},k)\\ & =\left(1-\frac{\Delta t}{T}\right){\hat{V}}_{a}(k-1)+\frac{\Delta t}{T}{\tilde{V}}_{a}(k)+\epsilon (k)\sigma_{V}\sqrt{\Delta t}, \end{array}$$

where *T* is a time constant, and where also normally distributed value noise ϵ(k) is injected, scaled to a standard deviation $\sigma_{V}$.

#### Noisy action value estimates

The ${\tilde{V}}_{a}$ are calculated as a probability-weighted sum over the other agent’s possible behaviors:


(4) $${\tilde{V}}_{a}(k)=\sum_{b}P_{b|a}(k){\tilde{V}}_{a|b}(k),$$

where $P_{b|a}$ is the estimated probability of the other agent exhibiting behavior *b* given that the ego agent chooses action *a* at the current time step, and ${\tilde{V}}_{a|b}$ is the noisy value estimate of action *a* given that the other agent exhibits behavior *b*.

#### Noisy action value estimates given behaviors of the other agent

The ${\tilde{V}}_{a|b}$ are calculated from the noisy perceived world state $\tilde{x}$:


(5) $${\tilde{V}}_{a|b}(k)=f_{s}(u_{ego}[\tilde{x}(k),(a,b)]),$$

where $f_{s}$ is a tanh sigmoid function constraining the value to [−1,1], and where $u_{ego}$ estimates the unconstrained value for the ego agent, in two steps: (1) A prediction of the states of both ego and other agent a fixed time $T_{P}=0.5$ s into the future, given their current positions and speeds as specified in $\tilde{x}(k)$, ego acceleration and any ego agent motor primitives previously triggered but not yet completed (i.e. the agent is keeping track of the current effects of its past actions on the world, in line with theories about efference copy and corollary discharge (59, 60)), the future action *a* being evaluated, and the acceleration corresponding to the other agent’s behavior *b*. (2) An estimation of the value of reaching the predicted state. By value, we mean the total, possibly time-discounted, future reward (40). We tested two alternative versions of this value estimation, as described below.

#### Short-term payoff values

Aligning with the classical game theory perspective of a single payoff after a one-shot decision by the players (18, 3), in our first value function variant, previously introduced in (61), we assume that value is composed solely of a reward payoff at the predicted time point $t(k)+T_{P}$. We assumed a reward function based on ego agent kinematics, collision aversion, and priority rules:


(6) $$u_{ego}[\tilde{x}(k),(a,b)]=K({\tilde{x}}_{P})-C({\tilde{x}}_{P})-R({\tilde{x}}_{P}),$$

where ${\tilde{x}}_{P}$ is the predicted world state given $\tilde{x}(k)$ and (a,b). The ego kinematics reward is defined as


(7) $$K({\tilde{x}}_{P})=k_{g}v-k_{\mathrm{dv}}v^{2}-k_{\mathrm{da}}a^{2},$$

where *v* and *a* are predicted ego speed and acceleration in ${\tilde{x}}_{P}$, and the $k_{∙}$ are reward function parameters (which we fix to yield human-like startup and equilibrium locomotion; Online Supplementary Fig. S3). The first term in Eq. 7 provides positive reward for making progress, whereas the second and third terms are costs from effort and discomfort associated with speed and acceleration, of a form familiar from many optimal control models of human motor and locomotor behavior (33, 62, 63). As for the remaining terms in Eq. 6, C quantifies the severity of an apparent collision course, and R, which in our tests is only relevant to driver agents in simulations with pedestrian priority, quantifies the extent to which current deceleration is insufficient to stop before the pedestrian crossing.

#### Affordance-based values

In our second value estimation scheme, we instead assume that the agent anticipates rewards over its entire, time-discounted, future (12, 13, 40), but we avoid a computationally expensive tree search of future actions by noting that in the space-sharing conflicts modeled here, the set of salient near-term futures for the agent can be plausibly limited to the the two access orders Ω∈{pass first, pass second} (31):


(8) $$u_{ego}[\tilde{x}(k),(a,b)]=\max_{\Omega }u_{ego}^{\prime }[\tilde{x}(k),(a,b),\Omega ],$$

where the value of each access order after applying action *a* (which as explained in the main text can be regarded as the extent to which *a* creates an *affordance* for Ω (41, 42)) is obtained by integrating over future rewards:


(9) $$\begin{array}{r} u_{ego}^{\prime }[\tilde{x}(k),(a,b),\Omega ]\;=\int_{t(k)}^{\infty }(K[{\tilde{x}}_{P}(t^{\prime })]-L[{\tilde{x}}_{P}(t^{\prime })])\cdot \delta (t^{\prime }-t(k))\cdot dt^{\prime }-R(\Omega ), \end{array}$$

with δ providing exponential discounting (40). In Eq. 9, K is exactly the same ego kinematics reward function as in Eq. 7, but ${\tilde{x}}_{P}(t^{\prime })$ now describes the predicted world state at future time $t^{\prime }$ not only given (a,b) but also given the ego agent access order Ω in question. Since the agent now has a concept of passing first or second, the priority rule cost R can be written directly in terms of Ω. Already with only K, Eq. 9 is collision-averse, obviating the need for the C in Eq. 7, but we also separately tested the impact of an additional cost L for experiencing visual looming (64), which was however not found crucial in our tests.

#### Noisy sensory input

We assume that the agent’s perceived world state $\tilde{x}(k)$ includes perfect estimates of own position, speed, and acceleration, and noisy estimates of the other agent’s position and speed:


(10) $$\tilde{x}(k)=P[x(k)].$$

where, in the maximally complex model, P involves both sensory noise (23, 65, 24) and Bayesian perceptual filtering. The ego agent observes the position of the other agent along its line of travel with Gaussian noise of standard deviation $\sigma_{x}(k)$, which is either constant $\sigma_{x}(k)=\sigma_{s}$, or varying with the true world state $\sigma_{x}=f_{v}[x(k)]$, in the latter case assuming that the agent estimates distance using visual angle under the horizon (66), with constant angular noise at the agent’s retina (24), i.e. in practice with larger position noise at greater distances.

#### Bayesian perceptual filtering

In line with theories of Bayesian perception (23, 67), we assume that the agent makes use of a Kalman filter (24) estimating both position and speed of the other agent from the noisy observations of position described above. We do not assume that $\tilde{x}(k)$ contains the maximum probability point estimate of the position and speed of the other agent, instead we assume that they are a random draw from the Kalman’s posterior distribution at time step *k*. This means that the noisy action values that get estimated from this perceptual input, and thereafter compared in the evidence accumulation decision-making, will span the distribution of action values for currently plausible world states. Since the value function is highly nonlinear, the average of the distribution of values estimated across the Kalman posterior is typically not the same as the value that would be estimated for the average of the Kalman posterior.

#### Behavior probabilities given actions

The probability $P_{b|a}$ that the other agent will exhibit behavior *b* given that the ego agent chooses action *a* is modeled as a normalized exponential of the evidence $A_{b|a}$ over the evidence for all behaviors (a softmax function), with all behavior evidence taken from the previous time step k−1:


(11) $$P_{b|a}(k)=S[\{A_{b^{\prime }|a}{\}}_{b^{\prime }},b,k]=\frac{e^{A_{b|a}(k-1)}}{\sum_{b^{\prime }}e^{A_{b^{\prime }|a}(k-1)}}.$$

We model the behavior evidence as a weighted sum of evidence $A_{V,b|a}$ from the estimated value of the behavior for the other agent, given that the ego agent chooses action *a*, and evidence $A_{O,b}$ from observation of the other agent:


(12) $$A_{b|a}(k)=\beta_{V}A_{V,b|a}(k)+\beta_{O}A_{O,b}(k),$$

where we can fix $\beta_{O}=1$ without loss of generality.

#### Behavior evidence from estimated behavior value given actions

The value-based evidence $A_{V,b|a}$ is defined simply as the noisy value ${\tilde{V}}_{b|a}$ for the other agent of behavior *b* given own action *a*, passed through the same type of evidence accumulation mechanism as for the ego agent’s value estimates in Eq. 3:


(13) $$A_{V,b|a}(k)={\hat{V}}_{b|a}(k)=A_{T,\sigma_{V}}({\hat{V}}_{b|a},{\tilde{V}}_{b|a},k),$$

where the noisy behavior value ${\tilde{V}}_{b|a}$ is calculated completely analogously to how the noisy action value ${\tilde{V}}_{a|b}$ is calculated for the ego agent in Eq. 5:


(14) $${\tilde{V}}_{b|a}(k)=u_{\mathrm{oth}}[\tilde{x}(k),(b,a)].$$

In practice, Eqs. 11 through 14 say that the ego agent judges the probability of the other agent’s behavior as the weighted exponential of the (accumulated) value of that behavior. This type of formulation is common in models of value-based intention attribution, for example in behavioral game theory (18, 22).

#### Behavior evidence from observation of the other agent

Based on theories of human Bayesian inference about behaviors (or intentions, or goals) of others from observation of their actions (19, 20, 68), we assume the following update equation for the observation-based evidence $A_{O,b}$:


(15) $$A_{O,b}(k)=\left(1-\frac{\Delta t}{T_{\mathrm{Of}}}\right)A_{O,b}(k-1)+\frac{\Delta t}{T_{O1}}\ln\;\;p[\tilde{x}(k)\;|\;\tilde{x}(k-1),b],$$

where $p[\tilde{x}(k)\;|\;\tilde{x}(k-1),b]$ is the probability of the perceived world state $\tilde{x}$ at the current time step *k* given that the other agent is currently exhibiting behavior *b*. These probabilities were modeled as normal distributions for the observed position of the other agent, with mean at the position predicted by $\tilde{x}(k-1)$ together with the acceleration corresponding to behavior *b*, and with standard deviation $\sigma_{O}$. $T_{\mathrm{Of}}$ is a forgetting time constant, determining how quickly old observation evidence is considered obsolete, and $T_{O1}$ represents the time needed for the human to perform one of these evidence updates; this time need not be identical to the model simulation time step Δt. It is demonstrated in the Online Supplementary Material that Eqs. 11 and 15 provide a generalized Bayesian update equation, such that Eqs. 11 and 15 together form a combined value-based and observation-based behavior estimation scheme, where values provide a prior for the observations, but a prior which can vary over time as the world state changes.

### Controlled experiments

Ethical approval for both experiments was given by the University of Leeds Research Ethics Committee (reference numbers LTTRAN-107 and AREA 21-022, respectively).

#### Experiment 1

In this experiment, which has been previously reported on in reference (1), (69), 60 participants (35 male, 25 female; ages 19–36 years, mean 27.3 years; all having lived at least one year in the UK) experienced repeated road-crossing scenarios in the University of Leeds Highly Immersive Kinematic Experimental Research (HIKER) laboratory, a 9×4 m high-fidelity CAVE pedestrian simulator with projection on three walls and floor. As schematically illustrated in the Online Supplementary Fig. S4A, the task given to the participants was to observe two vehicles approaching on a 3.5 m wide one-lane road, and cross between them if they felt comfortable to do so. The vehicles were initially driving at one of 25, 30, or 35 mph (11.2, 13.4, or 15.6 m/s), with a time gap between them of 2, 3, 4, or 5 s. In half of the trials, both vehicles maintained constant speed throughout, whereas in the other half of trials, the second vehicle yielded to the pedestrian with a constant deceleration starting and ending 38.5 and 2.5 m, respectively, from the pedestrian’s crossing location. In total, there were thus 3×4×2=24 different kinematic scenario variations.

After an initial practice block, each participant experienced three experimental blocks, where each block included two repetitions of each of the 24 kinematic scenario variations, with these 48 trials presented in a randomized order, different for each participant. In the original experiment, for 40 out of the 60 participants, in half of the trials where the second car yielded, it also displayed an external human–machine interface indicating its yielding intentions, but these trials were not included here, making for a total data set of 3×(20×48+40×36)=7,200 road-crossing trials, out of which 77 (1.1%) were excluded due to gaps in the recorded data. The crossing initiation time (as shown in Fig. 4A) in each included trial was measured as the time from when the rear of the first vehicle passed the crossing location, until when the participant began crossing the road.

#### Experiment 2

In this experiment, described in full detail in (70), the HIKER pedestrian simulator used in Experiment 1 was connected to the University of Leeds Driving Simulator (UoLDS), a high-fidelity simulator where the participant is seated in part of a Jaguar S-type car, housed within a 4 m diameter spherical projection dome with a $300^{\circ }$ field-of-view projection system, on an eight degree of freedom motion platform (a hexapod mounted on an XY translation table). This distributed simulation system, integrating the two simulators HIKER and UoLDS, allowed pedestrian and driver participants to interact in a shared virtual environment. As shown in the Online Supplementary Fig. S4B, the virtual scene was a two-lane road with 4.5 m wide lanes and a pedestrian refuge in the middle, with or without a zebra crossing. The pedestrian wore markers on their head and body to allow tracking their position and pose in the HIKER, rendered to the driver in the UoLDS as a set of colored spheres representing the pedestrian’s body motion (71).

A total of 32 pedestrians (ages 19–34 years, mean 25.1 years) and 32 drivers (ages 21–50 years, mean 31.5 years) were recruited into 32 pairs, with 8 pairs for each possible combination of genders in the driver and pedestrian roles. Both parties were informed that they would be interacting with another human in a number of road-crossing scenarios. They were instructed to handle the road-crossing interactions like they would in real traffic, imagining that they were late to a meeting (to minimize risk of some participants taking an overly passive role), and were reminded that a zebra crossing indicates pedestrian priority. The driver participants were instructed to consider the 30 mph (48 km/h) speed limit the same way they would in real traffic. The pedestrian participants were instructed to stand initially in each trial at a position where the two participants could not see each other due to a vision obstruction, and to then step up to the curb to look for oncoming traffic after hearing an auditory tone, from which point the participants could see each other and were free to interact as they saw fit. Unbeknownst to the participants, the auditory tone (which was only audible to the pedestrian participant) was triggered when the driver participant was at a certain time gap, one of 3, 4, 5, 6, or 7 s, from the pedestrian’s crossing location.

After a first practice block for just the driver participant, to get familiarized with the UoLDS, and a joint practice block involving both parties, each participant pair experienced two experimental blocks, each containing 20 trials, two repetitions of each of the 5×2=10 different scenarios (the different time gaps, and presence or not of a zebra crossing), in a randomized order, different for each participant pair. From the driver’s perspective, the interactions with the pedestrian were interspersed with similar vision obstructions and pedestrian crossing locations without a pedestrian present, to avoid the interactions being unnaturally predictable. In total, a data set of 32×2×20=1,280 interaction trials were recorded, out of which one (0.1%) was excluded due to technical problems. We analyzed the recorded data from each trial to observe who crossed first, pedestrian or driver (Fig. 4C).

### Model selection

Our model selection approach was based on exhaustive investigation of each model variant’s capability of reproducing a number of empirically observed phenomena, a previously advocated approach to testing of cognitive models (27, 72). Our method is similar in spirit to the “parameter subspace partitioning” method proposed in (27), but following that method exactly would have required us to predefine quantitative thresholds for each phenomenon, something which the available empirical evidence did not allow in all cases. Instead, we studied the behavior of each model variant across an entire plausible parameter space, using grid search. This approach also has the benefit of permitting a sensitivity analysis of the impact on model selection of any chosen quantitative thresholds, since the entire set of metric values across the parameter space is observed (as shown in Fig. 1B, and in for example Online Supplementary Figs. S5 and S13). The key findings from our model selection tests are presented in the main text of this paper, and the full results are provided in Section 2 of the Online Supplementary Material.

#### Deterministic model selection tests

Our first set of model selection tests addressed only those model assumptions which could be studied in deterministic simulation. In these tests, the model simulation time step was always Δt=0.1 s. We combined the short-term payoff and affordance-based value estimation assumptions with all possible combinations of the model assumptions about behavior estimation, making for a total of 36 tested model variants. We tested each variant in a grid search with ten values per free model parameter, logarithmically spaced within the parameter ranges listed in Table S2, which also provides motivations for the chosen range for each parameter. The most complex models in the deterministic tests had five free parameters, and were thus tested across 10^5^ parameterizations. It could be argued that this grid was relatively coarse, and could generate “false negatives,” i.e. overlooking some model variants’ abilities to express some behavioral phenomena. However, it should be borne in mind that our purpose here was not precise parameter-fitting to quantitative data, and if only a very small subset of a model’s plausible parameter space supports an empirically observed behavior pattern, then this is weak evidence in favor of the model, if compared to a model exhibiting the same pattern across a larger subset of its parameter space (27, 73). Nonetheless, we did further verify our main negative conclusions (about the short-term payoff models not being capable of priority assertion or short-stopping, and the maximally successful deterministic model not being capable of gap acceptance hesitation) using finer grids of 30 values per parameter for the models in question.

Across all of the tests mentioned above, we simulated each parameterization of each model variant to measure its ability of exhibiting the five targeted phenomena illustrated in Fig. 1A. For each phenomenon we defined a scenario, described in the Online Supplementary Table S3, where one agent (the agent expected to exhibit a certain behavior in the phenomenon in question) was controlled by the model, and the other agent’s behavior was predefined. For each such scenario, we tested three kinematic variants, by varying the initial TTA (time to arrival at the conflict space) of the driver agent by {−0.5,0,+0.5} s around the values mentioned in the Online Supplementary Table S3. This table also defines the metrics which we applied to the simulation results (see the *x* axes in Fig. 1B), to ascertain whether the modeled agent exhibited the sought-after behavior in any of the kinematic variants of each scenario. The vertical light blue lines and arrows in Fig. 1B indicate the metric value ranges indicative of the models exhibiting each phenomenon. We also defined quantitative thresholds for the metrics to determine which model parameterizations to reject or retain for the later nondeterministic analyses. These thresholds are also listed in the Online Supplementary Table S3, and were chosen to be inclusive, to rather retain too many parameterizations than too few. Full results across all scenario metrics are provided in Online Supplementary Figs. S5 and S6, with selected simulation examples in Online Supplementary Figs. S7–S10, and with retained parameterizations for the maximally successful deterministic model and two other model variants in the Online Supplementary Figs. S10–S12, respectively.

#### Stochastic model selection tests

One main aim of our tests of the stochastic model assumptions was to see whether they would permit the model to exhibit gap acceptance hesitation, since none of the most successful deterministic model variants could achieve this phenomenon. Therefore, we reused the same gap acceptance hesitation scenario as in the deterministic tests. However, rather than testing different kinematic variants of the scenario, due to the model stochasticity we instead simulated each tested model parameterization across five repetitions of the base kinematic variant of the scenario (as specified in the Online Supplementary Table S3).

While we hypothesized that adding noise to the model might make it capable of exhibiting gap acceptance hesitation, we also expected that excessive noise could generate collision-prone model behavior, particularly in interactive simulations, where both road users were controlled by the model. Therefore, in the stochastic tests we included three such scenarios (with the same parameterization for both road users, for simplicity). One of these scenarios was a two-agent version of the gap acceptance hesitation scenario, with exactly the same initial conditions. The other two interactive scenarios were “encounter” scenarios (5), where both agents initially travelled at their respective equilibrium speeds, both 3 s from the conflict space, i.e. with a clear collision conflict to be resolved. In one of these two encounter scenarios, the pedestrian had crossing priority. (These are the three scenarios in which the most successful model variant overall is simulated in Fig. 4B.) We simulated also these scenarios five times per tested parameterization, and observed whether collisions arose between the agents.

The inclusion of noise required a shorter simulation time step; we used Δt=0.025 s. We performed the stochastic model tests in two steps. First, we wanted to test whether the stochastic assumptions in themselves would be enough to achieve gap acceptance hesitation. Since the deterministic model tests rejected the short-term payoff value estimation scheme, we took the simplest possible model with affordance-based value estimation as our starting point, and tested it with all 10 possible combinations of our stochastic model assumptions. Just as in the deterministic tests, we performed a grid search with 10 values for each parameter; see Online Supplementary Table S4. The full results of these tests are described in the Online Supplementary Section 2.2.1 (Figs. S13–S15).

Second, we wanted to see whether we could identify stochastic model variants which included the assumptions successful in the deterministic tests, but which still exhibited both gap acceptance hesitation and were collision-free. We also wanted to obtain a set of model parameterizations that we could test against our controlled experiment data, as an artificial population including some variability in behavior, interpretable as describing road users with different characteristics. To this end, we tested all possible 24 combinations of the six deterministic model variants which achieved all of the targeted phenomena except gap acceptance hesitation (see Online Supplementary Section 2.1.1), with the four simple stochastic model variants which achieved gap acceptance hesitation and collision-free interactions in the tests described just above (see Online Supplementary Section 2.2.1). To obtain parameterizations for these models, we sampled from all possible combinations of the parameterizations that were found to achieve at least three of the targeted phenomena in the deterministic tests (example illustrations in the Online Supplementary Figs. S10–S12) with the parameterizations that met all of the criteria in the tests of the simple stochastic model variants (example illustrations in the Online Supplementary Figs. S14 and S15). The choice of requiring only three phenomena in the deterministic tests was made to promote variability in behavior, as mentioned above. For each of these 24 more complex stochastic model variants, we randomly selected 5,000 of these combined parameterizations (or less, in the few cases where the number of combined parameterizations was less than 5,000), and subjected these parameterizations to exactly the same simulated tests as described above for the simpler stochastic model variants. The full results of these tests are described in the Online Supplementary Section 2.2.2 (Online Supplementary Figs. S16 and S17). It should be noted that our approach here, of combining successful model variants and parameterizations from the deterministic and simple stochastic tests, is far from providing an exhaustive exploration of the possible behaviors of all stochastic model variants and parameterizations permitted by our modeling framework. However, for the specific aims outlined above, this simplified approach proved useful, and again we think the argument mentioned previously applies: Exhibiting a phenomenon across a relatively smaller region of model parameter space provides relatively weaker evidence in favor of the model in question.

### Tests on controlled experiment data

To compare the model’s behavior to the human behavior observed in the two controlled experiments, simulated scenarios were defined to closely replicate the experimental settings. For Experiment 1, where each scenario was defined by the vehicle speed and time gap, all simulations were initialized with the pedestrian at zero speed, 1 m from the conflict space, with the two approaching vehicles at their initial speed and at distances such that the first vehicle was 3 s from completely passing the pedestrian (to allow the model, like the participants in the study, to see the second car for a while before the first car passed), and the second vehicle following its designated time gap later. The experimental instruction to the pedestrian to not pass before the first vehicle was in practice implemented by adding −∞ to the unconstrained value *u* (before the arctan sigmoid) of passing before the second vehicle, during that part of the simulation when the first vehicle had not yet passed the pedestrian. For each of the 23 complex stochastic model variants that met the retention criteria described in the previous section (reported on in the Online Supplementary Section 2.2.2), 500 of the retained parameterizations were drawn at random, and each such parameterization was tested on all scenarios in Experiment 1, with 6 repetitions of each scenario, as for the human participants. The pedestrian crossing initiation time in each scenario was measured as the time at which the pedestrian agent began moving.

For Experiment 2, where the experimental scenarios were defined by the initial vehicle time gap and the presence or not of a zebra crossing indicating pedestrian priority, all simulations were initialized with the pedestrian at zero speed, 1.95 m from the conflict space, with the vehicle at 30 mph and at a temporal distance corresponding to the time gap for the scenario in question, minus one second. These initial conditions were chosen to reflect the typical observed response delay of the human pedestrian participants between hearing the auditory tone at the start of the trial and reaching the point from which driver and pedestrian participants were able to see each other. We tested our maximally successful model variant on these scenarios, again drawing 500 parameterizations at random from those retained in the model selection tests described above and in the Online Supplementary Section 2.2.2.

In both of the tests described above, model parameterizations were excluded which were found to generate nonprogressing agent behavior, i.e. where the agents got stuck at zero speed. For Experiment 1, nonprogression was defined as the pedestrian agent not crossing even after the car had fully yielded, and for Experiment 2 it was defined as at least one of the two agents not entering the conflict space within 20 s of the start of the simulation. The full results of these tests are described in the Online Supplementary Section 2.3 (Figs. S18–S20).

## Supplementary Material

pgad163_Supplementary_Data

## Data Availability

All data, and all model and analysis code, are available at https://doi.org/10.17605/OSF.IO/ZMK9T.

## Preprints

This manuscript was posted as a preprint at: https://doi.org/10.31234/osf.io/hdxbs

## References

[1] Pekkanen J . 2022. Variable-drift diffusion models of pedestrian road-crossing decisions. Comput Brain Behav. 5(1):60–80.

[2] Gorrini A, Crociani L, Vizzari G, Bandini S. 2018. Observation results on pedestrian-vehicle interactions at non-signalized intersections towards simulation. Trans Res F: Traffic Psychol Behav. 59:269–285.

[3] Elvik R . 2014. A review of game-theoretic models of road user behaviour. Accid Anal Prev. 62:388–396. 23838049 10.1016/j.aap.2013.06.016

[4] Chen W, Zhuang X, Cui Z, Ma G. 2019. Drivers’ recognition of pedestrian road-crossing intentions: performance and process. Trans Res F: Traffic Psychol Behav. 64:552–564.

[5] Várhelyi A . 1998. Drivers’ speed behaviour at a zebra crossing: a case study. Accid Anal Prev. 30(6):731–743. 9805516 10.1016/s0001-4575(98)00026-8

[6] Domeyer J . 2019. Proxemics and kinesics in automated vehicle–pedestrian communication: representing ethnographic observations. Transp Res Rec. 2673(10):70–81.

[7] Lee YM . 2021. Road users rarely use explicit communication when interacting in today’s traffic: implications for automated vehicles. Cogn Technol Work. 23(2):367–380.

[8] Camara F . 2020. Pedestrian models for autonomous driving part II: high-level models of human behavior. IEEE Trans Intell Transp Syst. 22(9):5453–5471.

[9] Ettinger S . 2021. Large scale interactive motion forecasting for autonomous driving: the Waymo open motion dataset. In: 2021 IEEE/CVF International Conference on Computer Vision (ICCV), October, p. 9690–9699. ISSN:2380-7504.

[10] Suo S, Regalado S, Casas S, Urtasun R. 2021. TrafficSim: learning to simulate realistic multi-agent behaviors. In: Proceedings of the IEEE/CVF Conference on Computer Vision and Pattern Recognition (CVPR). p. 10400–10409.

[11] Feng S, Yan X, Sun H, Feng Y, Liu HX. 2021. Intelligent driving intelligence test for autonomous vehicles with naturalistic and adversarial environment. Nat Commun. 12(1):748. 33531506 10.1038/s41467-021-21007-8 PMC7854639

[12] Sadigh D, Landolfi N, Sastry SS, Seshia SA, Dragan AD. 2018. Planning for cars that coordinate with people: leveraging effects on human actions for planning and active information gathering over human internal state. Auton Robots. 42(7):1405–1426.

[13] Schwarting W, Pierson A, Alonso-Mora J, Karaman S, Rus D. 2019. Social behavior for autonomous vehicles. Proc Natl Acad Sci USA. 116(50):24972–24978. 31757853 10.1073/pnas.1820676116 PMC6911195

[14] Domeyer JE, Lee JD, Toyoda H, Mehler B, Reimer B. 2022. Driver-pedestrian perceptual models demonstrate coupling: implications for vehicle automation. IEEE Trans Human-Mach Syst. 24, 1–10.

[15] Prédhumeau M, Mancheva L, Dugdale J, Spalanzani A. 2022. Agent-based modeling for predicting pedestrian trajectories around an autonomous vehicle. J Artif Intell Res. 73:1385–1433.

[16] Ratcliff R, Smith PL, Brown SD, McKoon G. 2016. Diffusion decision model: current issues and history. Trends Cogn Sci. 20(4):260–281. 26952739 10.1016/j.tics.2016.01.007 PMC4928591

[17] Busemeyer JR, Gluth S, Rieskamp J, Turner BM. 2019. Cognitive and neural bases of multi-attribute, multi-alternative, value-based decisions. Trends Cogn Sci. 23(3):251–263. 30630672 10.1016/j.tics.2018.12.003

[18] Wright JR, Leyton-Brown K. 2017. Predicting human behavior in unrepeated, simultaneous-move games. Games Econ Behav. 106:16–37.

[19] Baker CL, Saxe R, Tenenbaum JB. 2009. Action understanding as inverse planning. Cognition. 113(3):329–349. 19729154 10.1016/j.cognition.2009.07.005

[20] Pezzulo G, Donnarumma F, Dindo H. 2013. Human sensorimotor communication: a theory of signaling in online social interactions. PLoS ONE. 8(11):e79876. 24278201 10.1371/journal.pone.0079876 PMC3835897

[21] Levy DJ, Glimcher PW. 2012. The root of all value: a neural common currency for choice. Curr Opin Neurobiol. 22(6):1027–1038. 22766486 10.1016/j.conb.2012.06.001 PMC4093837

[22] Jara-Ettinger J, Schulz LE, Tenenbaum JB. 2020. The Naïve Utility Calculus as a unified, quantitative framework for action understanding. Cogn Psychol. 123:101334. 32738590 10.1016/j.cogpsych.2020.101334

[23] Knill DC, Pouget A. 2004. The Bayesian brain: the role of uncertainty in neural coding and computation. Trends Neurosci. 27(12):712–719. 15541511 10.1016/j.tins.2004.10.007

[24] Kwon O-S, Tadin D, Knill DC. 2015. Unifying account of visual motion and position perception. Proc Natl Acad Sci USA. 112(26):8142–8147. 26080410 10.1073/pnas.1500361112 PMC4491751

[25] Newell A . 1973. You can’t play 20 questions with nature and win. In: Chase WG, editor. Visual information processing. New York: Academic Press. p. 283–308.

[26] Robinaugh DJ, Haslbeck JMB, Ryan O, Fried EI, Waldorp LJ. 2021. Invisible hands and fine calipers: a call to use formal theory as a toolkit for theory construction. Perspect Psychol Sci. 16(4):725–743. 33593176 10.1177/1745691620974697 PMC8273080

[27] Pitt MA, Kim W, Navarro DJ, Myung JI. 2006. Global model analysis by parameter space partitioning. Psychol Rev. 113(1):57–83. 16478301 10.1037/0033-295X.113.1.57

[28] Rasouli A, Kotseruba I, Tsotsos JK. 2018. Understanding pedestrian behavior in complex traffic scenes. IEEE Trans Intell Veh. 3(1):61–70.

[29] Risto M, Emmenegger C, Vinkhuyzen E, Cefkin M, Hollan J. 2017. Human-vehicle interfaces: the power of vehicle movement gestures in human road user coordination. In: Proceedings of the Ninth International Driving Symposium on Human Factors in Driver Assessment, Training and Vehicle Design, Manchester Village, Vermont. p. 186–192.

[30] Portouli E, Nathanael D, Marmaras N. 2014. Drivers’ communicative interactions: on-road observations and modelling for integration in future automation systems. Ergonomics. 57(12):1795–1805. 25204887 10.1080/00140139.2014.952349

[31] Markkula G . 2020. Defining interactions: a conceptual framework for understanding interactive behaviour in human and automated road traffic. Theor Issues Ergon Sci. 21(6):728–752.

[32] Giszter SF . 2015. Motor primitives—new data and future questions. Curr Opin Neurobiol. 33:156–165. 25912883 10.1016/j.conb.2015.04.004 PMC6524953

[33] Gawthrop P, Loram I, Lakie M, Gollee H. 2011. Intermittent control: a computational theory of human control. Biol Cybern. 104(1–2):31–51. 21327829 10.1007/s00422-010-0416-4

[34] Markkula G, Boer E, Romano R, Merat N. 2018. Sustained sensorimotor control as intermittent decisions about prediction errors: computational framework and application to ground vehicle steering. Biol Cybern. 112(3):181–207. 29453689 10.1007/s00422-017-0743-9 PMC6002515

[35] Svärd M, Markkula G, Bärgman J, Victor T. 2021. Computational modeling of driver pre-crash brake response, with and without off-road glances: parameterization using real-world crashes and near-crashes. Accid Anal Prev. 163:106433. 34673380 10.1016/j.aap.2021.106433

[36] Thorstensson A, Roberthson H. 1987. Adaptations to changing speed in human locomotion: speed of transition between walking and running. Acta Physiol Scand. 131(2):211–214. 10.1111/j.1748-1716.1987.tb08228.x 3673618

[37] Hase K, Stein RB. 1998. Analysis of rapid stopping during human walking. J Neurophysiol. 80(1):255–261. 9658047 10.1152/jn.1998.80.1.255

[38] Robin Th, Antonini G, Bierlaire M, Cruz J. 2009. Specification, estimation and validation of a pedestrian walking behavior model. Trans Res B: Methodol. 43(1):36–56.

[39] Whiten A, Byrne RW. 1991. Natural theories of mind: evolution, development and simulation of everyday mindreading. UK: B. Blackwell Oxford.

[40] Sutton RS, Barto AG. 2018. Reinforcement learning: an introduction. 2nd ed. Cambridge (MA): The MIT Press.

[41] Pezzulo G, Cisek P. 2016. Navigating the affordance landscape: feedback control as a process model of behavior and cognition. Trends Cogn Sci. 20(6):414–424. 27118642 10.1016/j.tics.2016.03.013

[42] Fajen BR . 2008. Perceptual learning and the visual control of braking. Percept Psychophys. 70(6):1117–1129. 18717396 10.3758/pp.70.6.1117

[43] Montufar J, Arango J, Porter M, Nakagawa S. 2007. Pedestrians’ normal walking speed and speed when crossing a street. Transp Res Rec. 2002(1):90–97.

[44] Crosato L, Shum HPH, Ho ESL, Wei C. 2023. Interaction-aware decision-making for automated vehicles using social value orientation. IEEE Trans Intell Veh. 8(2):1339–1349.

[45] Laird JE, Newell A, Rosenbloom PS. 1987. SOAR: an architecture for general intelligence. Artif Intell. 33(1):1–64.

[46] Anderson JR . 2004. An integrated theory of the mind. Psychol Rev. 111(4):1036–1060. 15482072 10.1037/0033-295X.111.4.1036

[47] Salvucci DD . 2006. Modeling driver behavior in a cognitive architecture. Hum Factors. 48(2):362–380. 16884055 10.1518/001872006777724417

[48] Salvucci DD . 2009. Rapid prototyping and evaluation of in-vehicle interfaces. ACM Trans Comput Hum Interact. 16(2):1–33.22563232

[49] de Dreu CKW, van Lange PAM. 1995. The impact of social value orientations on negotiator cognition and behavior. Pers Soc Psychol Bull. 21(11):1178–1188.

[50] Usher M, McClelland JL. 2001. The time course of perceptual choice: the leaky, competing accumulator model. Psychol Rev. 108(3):550–592. 11488378 10.1037/0033-295x.108.3.550

[51] Nunes LF, Gurney K. 2016. Multi-alternative decision-making with non-stationary inputs. R Soc Open Sci. 3(8):160376. 27853619 10.1098/rsos.160376 PMC5108969

[52] Dayan P, Berridge KC. 2014. Model-based and model-free Pavlovian reward learning: revaluation, revision, and revelation. Cogn Affect Behav Neurosci. 14(2):473–492. 24647659 10.3758/s13415-014-0277-8 PMC4074442

[53] Wilson AD, Golonka S. 2013. Embodied cognition is not what you think it is. Front Psychol. 4:58. 23408669 10.3389/fpsyg.2013.00058 PMC3569617

[54] Hayden BY, Niv Y. 2021. The case against economic values in the orbitofrontal cortex (or anywhere else in the brain). Behav Neurosci. 135(2):192–201. 34060875 10.1037/bne0000448 PMC12398331

[55] Gulzar M, Muhammad Y, Muhammad N. 2021. A survey on motion prediction of pedestrians and vehicles for autonomous driving. IEEE Access. 9:137957–137969.

[56] Rahwan I . 2019. Machine behaviour. Nature. 568(7753):477–486. 31019318 10.1038/s41586-019-1138-y

[57] Siebinga O, Zgonnikov A, Abbink D. 2022. A human factors approach to validating driver models for interaction-aware automated vehicles. ACM Trans Hum-Robot Interact. 11(4):47.

[58] Srinivasan AR . 2022. Beyond RMSE: do machine-learned models of road user interaction produce human-like behavior?, arXiv, arXiv:2206.11110, preprint: not peer reviewed.

[59] Franklin DW, Wolpert DM. 2011. Computational mechanisms of sensorimotor control. Neuron. 72(3):425–442. 22078503 10.1016/j.neuron.2011.10.006

[60] Straka H, Simmers J, Chagnaud BP. 2018. A new perspective on predictive motor signaling. Curr Biol. 28(5):R232–R243. 29510116 10.1016/j.cub.2018.01.033

[61] Lin Y-S, Srinivasan AR, Leonetti M, Billington J, Markkula G. 2022. A utility maximization model of pedestrian and driver interactions. IEEE Access. 10:118888–118899.

[62] Wang M, Hoogendoorn SP, Daamen W, van Arem B, Happee R. 2015. Game theoretic approach for predictive lane-changing and car-following control. Trans Res C: Emerg Technol. 58:73–92.

[63] Hoogendoorn S, Bovy PHL. 2003. Simulation of pedestrian flows by optimal control and differential games. Optim Control Appl Methods. 24(3):153–172.

[64] Tian K . 2022. Explaining unsafe pedestrian road crossing behaviours using a psychophysics-based gap acceptance model. Saf Sci. 154:105837.

[65] Brunton BW, Botvinick MM, Brody CD. 2013. Rats and humans can optimally accumulate evidence for decision-making. Science. 340(6128):95–98. 23559254 10.1126/science.1233912

[66] Ooi TL, Wu B, He ZJ. 2001. Distance determined by the angular declination below the horizon. Nature. 414(6860):197–200. 11700556 10.1038/35102562

[67] Fetsch CR, DeAngelis GC, Angelaki DE. 2013. Bridging the gap between theories of sensory cue integration and the physiology of multisensory neurons. Nat Rev Neurosci. 14(6):429–442. 23686172 10.1038/nrn3503 PMC3820118

[68] Dindo H, Zambuto D, Pezzulo G. 2011. Motor simulation via coupled internal models using sequential Monte Carlo. In: Proceedings of IJCAI 2011, Barcelona, Spain. p. 2113–2119.

[69] Lee YM . 2022. Learning to interpret novel eHMI: the effect of vehicle kinematics and eHMI familiarity on pedestrian’ crossing behavior. J Safety Res. 80:270–280. 35249607 10.1016/j.jsr.2021.12.010

[70] Kalantari AH . 2022. Who goes first? A distributed simulator study of vehicle-pedestrian interaction.

[71] Sadraei E . 2020. Vehicle-pedestrian interaction: a distributed simulation study. In: Proceedings of the Driving Simulation 2020 Europe Conference; Antibes, France. p. 147–1154.

[72] Navarro DJ . 2019. Between the devil and the deep blue sea: tensions between scientific judgement and statistical model selection. Comput Brain Behav. 2(1):28–34.

[73] Roberts S, Pashler H. 2000. How persuasive is a good fit? A comment on theory testing. Psychol Rev. 107(2):358–367. 10789200 10.1037/0033-295x.107.2.358

