# Correction to: Explaining human interactions on the road by large-scale integration of computational psychological theory

**DOI:** 10.1093/pnasnexus/pgad377

**Published:** 2023-11-15

This is a correction to: Gustav Markkula, Yi-Shin Lin, Aravinda Ramakrishnan Srinivasan, Jac Billington, Matteo Leonetti, Amir Hossein Kalantari, Yue Yang, Yee Mun Lee, Ruth Madigan, Natasha Merat, Explaining human interactions on the road by large-scale integration of computational psychological theory, *PNAS Nexus*, Volume 2, Issue 6, June 2023, pgad163, https://doi.org/10.1093/pnasnexus/pgad163

During a retroactive audit conducted by *PNAS Nexus*, it was discovered that this paper was missing a statement acknowledging compliance with the *PNAS Nexus* Human and Animal Participants and Clinical Trials policy:

Informed consent was obtained from all participants prior to their participation.

This error has been corrected in the original article.

