# Supplementary material for: Explaining human interactions on the road by large-scale integration of computational psychological theory
Source: PNAS Nexus. 2023 Jun 20;2(6):pgad163. doi: 10.1093/pnasnexus/pgad163 (PMC10281388; doi:10.1093/pnasnexus/pgad163)
Supplement: pgad163_Supplementary_Data [file pgad163_supplementary_data.pdf]

Supplementary information for:

Explaining human interactions on the road by large-scale integration of computational psychological theory

Gustav Markkula, Yi-Shin Lin, Aravinda Ramakrishnan Srinivasan, Jac Billington, Matteo Leonetti, Amir Hossein Kalantari, Yue Yang, Yee Mun Lee, Ruth Madigan, Natasha Merat

Contents

1 Model framework – full details and explanatory notes **2**
1.1 Movement control by motor primitives . . . 2
1.2 Action decisions based on accumulated action value estimates . . . 3
1.3 Noisy action value estimates . . . 3
1.4 Behaviours of the other agent . . . 3
1.5 Noisy action value estimates given behaviours of the other agent . . . 4
1.6 Short-term payoff values . . . 4
1.7 Affordance-based values . . . 5
1.8 Noisy sensory input . . . 6
1.9 Bayesian perceptual filtering . . . 6
1.10 Behaviour probabilities given actions . . . 6
1.11 Behaviour evidence from estimated behaviour value given actions . . . 6
1.12 Behaviour evidence from observation of the other agent . . . 7
1.13 Summary of model assumptions . . . 8

2 Model selection – full results **8**
2.1 Deterministic tests . . . 8
2.1.1 Models achieving individual phenomena . . . 8
2.1.2 Model variants achieving multiple phenomena . . . 17
2.1.3 Additional tests of negative results . . . 20
2.2 Stochastic tests . . . 20
2.2.1 Simple model variants, with minimal deterministic assumptions . . . 20
2.2.2 Combining with more complex deterministic assumptions . . . 22
2.3 Tests on controlled experiment data . . . 24

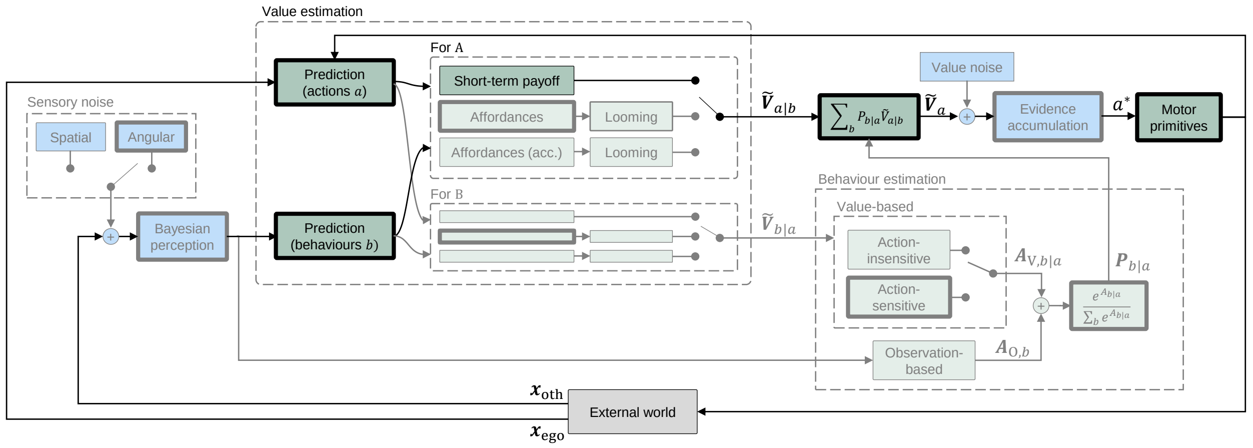

Figure S1: The full modular model framework. All model assumptions are shown: Those included in the base model (dark green), the deterministic assumptions (dark and light green), the stochastic assumptions (light blue), and the assumptions included in the maximally successful model (bold edges; also shown in Fig. 2 in the main paper). The "switches" in the illustration indicate mutually exclusive assumptions.

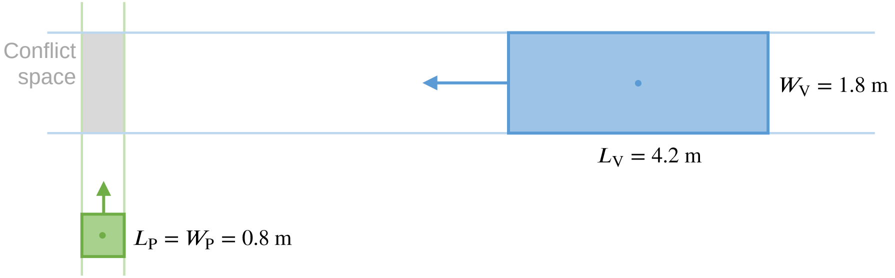

Figure S2: The basic geometry of all pedestrian-vehicle interaction scenarios studied here. Both car and pedestrian are represented as rectangles moving forward only (or standing still), with a conflict space arising at the intersection of their trajectories.

1 Model framework – full details and explanatory notes

A Python implementation of the model framework is available at `https://doi.org/10.17605/OSF.IO/ZMK9T`. Abbreviations will be introduced here for referring to the optional assumptions; e.g., `oEA` for evidence accumulation, `oSNv` for visual sensory noise, and so on. These abbreviations are used both in these supplementary materials and in the implementation of the model.

Below, additional detail and explanations are provided for the various parts of the model; most subsection headings below map to paragraph headings in the model description provided in the Methods section of the main paper.

1.1 Movement control by motor primitives

In Eq. (1) in the main text, G defines the stereotypical shape of the motor primitive used by the agent, here assumed to be a linear unity step increase of duration ΔT:

$$G(k) = \min\left(1, \frac{k\Delta t}{\Delta T}\right), \qquad \text{(S1)}$$

where Δt is the model simulation time step.

We assume the control signal C to be acceleration in the case of the driver agent, and speed in the case of the pedestrian agent. In other words, we are assuming that the default state of driver and pedestrian is to maintain their current acceleration and speed respectively, but that they can intermittently decide to apply linear adjustments to these quantities. This is in line with naturalistic observations of intermittent stepwise pedal adjustments by drivers [35,74] and stepwise walking speed changes by pedestrians [2,36–38]. In the latter case the walking speed adjustments could be regarded as higher-level motor primitives, for example constructing a supraspinal walking speed signal as an input to spinal gait control circuits [75].

Loosely based on existing naturalistic data [2,35,38,74] we fix $\Delta T = 0.5$ s for both driver and pedestrian, and the set of alternative actions we consider for the agents are the motor primitive amplitudes of $\{-1, -0.5, 0, 0.5, 1\}$, in m/s^2 and m/s, respectively. Note that by superpositioning these motor primitives, the agents can achieve larger-amplitude adjustments, or control that looks more continuous than intermittent (as can be seen for example in Fig. S3 or Figs. S7–S9, the intermittent control can "masquerade" as continuous control [33]).

1.2 Action decisions based on accumulated action value estimates

Eq. (2) and Eq. (3) in the main text define a special case of the type of evidence accumulation process that has been much studied in models of perceptual and value-based decision-making [16, 17, 50], here with decision variable

$$\Delta V_a(k) = \hat{V}_a(k) - \hat{V}_{a_\varnothing}(k) \tag{S2}$$

and decision boundary ΔV_{th}. The three terms on the right hand side of Eq. (3) should be recognisable from existing evidence accumulation models [50] as a leakage term (i.e., in practice implementing a form of forgetting [51]), an evidence input (or drift) term, and a noise (or diffusion) term, respectively. Note that in our formulation, the relative scaling of the leakage and input terms has been constrained such that if the input value $\tilde{V}_a$ is constant, the accumulated value $\hat{V}_a$ will converge to $\tilde{V}_a$ over time. In other words, $\mathscr{A}_{T,\sigma_\text{V}}$ is effectively a first order low-pass filter with time constant T, and the accumulated value $\hat{V}_a$ is a low-pass filtered version of the momentary value estimate $\tilde{V}_a$, but with added value noise (the third term in Eq. (3)).

We set the decision boundary indirectly, as $\Delta V_{\text{th}} = \Delta V_{\text{th,rel}} V_{\text{free}}$, where $\Delta V_{\text{th,rel}}$ is a model parameter, and V_{free} is the value for the agent of being at its equilibrium speed without another agent present (see Section 1.5). Once an action other than $a_\varnothing$ is selected by an agent, all accumulated action values $\hat{V}_a$ are reset to zero.

We defined the following optional model assumptions:

- **Evidence accumulation** (`oEA`), enabled for $T > \Delta t$ and $\Delta V_{\text{th,rel}} > 0$.
- **Value noise** (injected as accumulator noise; `oAN`), enabled for $\sigma_\text{V} > 0$.

1.3 Noisy action value estimates

Note that we describe the value estimates in Eq. (4) in the main text as noisy because they are based, in the maximally complex model, on noisy perceptual input. They are only actually noisy if one of the assumptions `oSNc` or `oSNv` is included.

1.4 Behaviours of the other agent

A behaviour of the other agent is in practice defined as a constant acceleration from the current time step k. With different assumptions included in the model, different possible behaviours are considered:

If neither observation-based behaviour estimation (`oBEo`) nor value-based behaviour estimation (`oBEv`) is assumed, only the single behaviour $b_\varnothing$ of the other agent maintaining its current speed, i.e., a zero acceleration, is considered.

If the model variant assumes either or both types of behaviour estimation, two behaviours of the other agent are considered, corresponding to the two possible access orders: passing before or after the ego agent. In practice, the acceleration needed for the other agent to pass first(/second) is defined as the acceleration that is needed, given the current positions and speeds of the two agents, in order for the other agent to be a distance margin of D_s beyond(/before) the conflict space (see Fig. S2) a time margin T_s before(/after) the ego agent enters(/exits) the conflict space. Loosely based on data on "post encroachment times" in naturalistic pedestrian-vehicle encounters [76], we fixed $D_\text{s} = 1$ m and $T_\text{s} = 1$ s. There are also some special cases to consider: (i) The behaviour of passing second may in practice have the other agent stop completely at distance D_s before the conflict space, more than T_s before the ego agent's arrival. (ii) If the other agent can achieve one of the access orders by simply accelerating to and thereafter maintaining its equilibrium speed, the behaviour for that access order is defined this way instead. This regaining of equilibrium speed is assumed done in $\Delta T = 0.5$ s for the pedestrian agent (since this is the speed with which the pedestrian is assumed to always adjust its speed), and for simplicity always in 10 s for the driver agent (strictly speaking the model predicts that different deviations from equilibrium speed will be rectified by the driver agent in different amounts of time, but this simplification here does not make much difference in practice to the estimated behaviour accelerations).

We also tested an additional optional model assumption, only applicable in model variants assuming behaviour estimation (i.e., either or both of `oBEo` or `oBEv` are enabled):

- **Estimation of constant speed behaviour** (`oBEc`), also considering the constant speed behaviour $b_\varnothing$ in addition to the pass first/second behaviours, i.e., considering three possible behaviours in total.

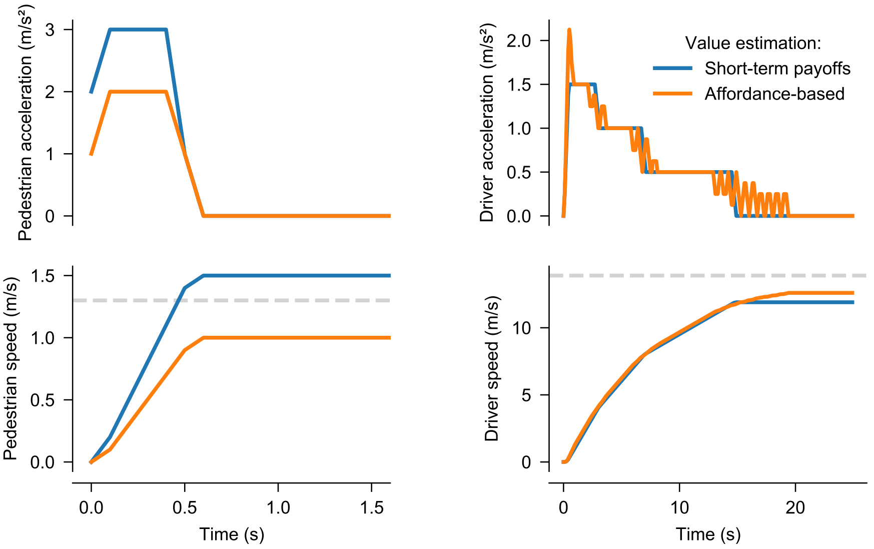

Figure S3: Acceleration behaviour of the pedestrian and driver agents when starting from standstill, without the other agent present, for the two value estimation schemes studied here. The obtained startup behaviour is qualitatively consistent with empirically observed human behaviour: Pedestrians typically reach normal steady-state walking speed within 0.5-1 s [77, 78], and car drivers starting from standstill tend to apply an early peak of acceleration within the first few seconds, at about 1-3 m/s^2, and then scale down acceleration gradually, over a time scale of about 10-20 s [79, 80]. Note that due to acceleration discomfort costs and the discretised action space, the agents typically do not converge exactly to their theoretical equilibrium speeds (the dashed horizontal lines). With short-term payoff value estimation, the pedestrian does not experience acceleration discomfort, hence the higher steady-state walking speed.

1.5 Noisy action value estimates given behaviours of the other agent

In Eq. (5) in the main text, f_s is the sigmoid:

$$f_\mathrm{s}(u) = \tanh \frac{u}{u_0} \tag{S3}$$

where u_0 is a parameter which we fixed to $u_0 = 4u_\mathrm{free}$, where u_free is the unconstrained value for the agent of being at equilibrium speed without another agent present. This corresponds to $V_\mathrm{free} = f_\mathrm{s}(u_\mathrm{free}) \approx 0.24$, such that the constrained values in $[-1, 1]$ cover a range both above and considerably below V_free.

1.6 Short-term payoff values

In the absence of collision courses ($\mathscr{C} = 0$) and priority concerns ($\mathscr{R} = 0$), the maximum attainable unconstrained value u for an agent arises when it is predicted to be moving at its equilibrium speed (obtainable by differentiating Eq. (7) in the main text with respect to v) of $v_\mathrm{free} = k_\mathrm{g}/2k_\mathrm{dv}$. We fixed the parameters k_g and k_dv so as to achieve equilibrium speeds of $v_\mathrm{p,free} = 1.3$ m/s for the pedestrian [77, 78] and $v_\mathrm{v,free} = 50$ km/h ≈ 13.9 m/s for the driver, and such that the reward for being in this equilibrium state was, for both agents, normalised to $\mathscr{K} = 1$ (without loss of generality, since the reward is in arbitrary units), which means that also $u_\mathrm{free} = 1$.

We fixed $k_\mathrm{da} = 0.01$, to obtain vehicle startup accelerations from standstill reasonably in line with naturalistic driver behaviour, as shown in Fig. S3. Since in our simulations we used the same value (0.5 s) both for the value prediction time T_P and the motor primitive duration ΔT, the predicted ego acceleration of the pedestrian is always zero, so k_da does not affect the pedestrian with short-term payoff value estimation.

The collision aversion cost $\mathscr{C}$ in Eq. (6) in the main text is zero if the predicted positions and speeds of the agents in $\tilde{\mathbf{x}}_\mathrm{P}$ do not have them at a collision course, but if there is a collision course, with time to collision τ, then for the pedestrian agent the following formulation is used:

$$\mathscr{C}(\tilde{\mathbf{x}}_\mathrm{P}) = \frac{k_\mathrm{c}}{\tau}, \tag{S4}$$

based on literature suggesting human collision aversion is informed by optical cues approximating time to collision, or its inverse as above [81, 82]. For the driver agent, we instead follow literature suggesting that drivers are sensitive to optical cues approximating the deceleration d_stop required to stop without collision [42],

and formulate the collision aversion as another acceleration discomfort cost:

$$\mathscr{C}(\tilde{\mathbf{x}}_{\mathrm{P}}) = k_{\mathrm{sc}} d_{\mathrm{stop}}^2 = k_{\mathrm{sc}} \frac{v^2}{4\tau^2}. \quad \text{(S5)}$$

The priority rule cost $\mathscr{R}$ is always zero for the pedestrian in our studied scenarios, but if there is priority for the pedestrian (zebra crossing), this cost is nonzero for the vehicle agent if the predicted acceleration of the vehicle a is larger than the acceleration a_{2nd} needed to pass second (as defined in Section 1.4). If so:

$$\mathscr{R}(\tilde{\mathbf{x}}_{\mathrm{P}}) = \left(2 - \frac{a}{a_{\mathrm{2nd}}}\right) \cdot c \cdot u_{\mathrm{free}}, \quad \text{(S6)}$$

such that the cost for not braking enough to yield to the pedestrian starts at $c \cdot u_{\mathrm{free}}$ and increases for even higher predicted accelerations. We fixed $c = 1.5$, implying that the cost for the driver of not yielding to a pedestrian who has priority is larger than the value for the driver of progressing at equilibrium speed.

1.7 Affordance-based values

The exponential time discounting of future rewards [40] in Eq. (9) in the main text is defined as:

$$\delta(t) = 2^{-t/T_\delta}, \quad \text{(S7)}$$

with time constant a model parameter T_δ.

For the first interval T_{P} into the future, the predicted world state $\tilde{\mathbf{x}}_{\mathrm{P}}(t')$ in Eq. (9) is the same prediction of the outcome of action a as in the short-term payoff value estimation. However, thereafter the future ego agent kinematics in $\tilde{\mathbf{x}}_{\mathrm{P}}(t')$ are described by first the constant acceleration needed to achieve access order Ω, calculated exactly as described in Section 1.4 for the behaviours of the other agent, and thereafter, if needed, an interval of waiting for the other agent to pass (only if Ω is 'pass second' and the kinematics demand it), and finally, if needed, an interval of constant acceleration to regain the equilibrium speed, also as previously described. An example illustration is provided in Fig. 3C in the main paper; for each combination of own action, behaviour of the other agent, and access order, the future kinematics are predicted, and the corresponding rewards and values are calculated. (In the example in Fig. 3C, the outcome of "passing before a crossing pedestrian" is not available, since in the given situation a crossing pedestrian would already be within the conflict space after the initial prediction interval T_{P}, so it is no longer possible for the driver to pass first.)

Note that given Eq. (9), the value of being at equilibrium speed (such that $\mathscr{K} = 1$, as described in Section 1.6), without another agent present ($\mathscr{L} = \mathscr{O} = 0$), is:

$$u_{\mathrm{free}} = \int_{t(k)}^{\infty} \delta(t' - t(k)) \cdot \mathrm{d}t' = \frac{T_\delta}{\ln 2} \quad \text{(S8)}$$

Based on findings that human road users are sensitive to the visual looming (optical expansion rate) $\dot{\theta}$ of collision threats [64] we also included a discomfort cost for any visual looming experienced during the predicted time horizon:

$$\mathscr{L}[\tilde{\mathbf{x}}_{\mathrm{P}}] = \frac{\dot{\theta}}{\dot{\theta}_1}, \quad \text{(S9)}$$

where $\dot{\theta}$ is the visual looming experienced by the ego agent in the predicted world state $\tilde{\mathbf{x}}_{\mathrm{P}}$ ($\dot{\theta} = 0$ if there is no collision course), and $\dot{\theta}_1$ is a model parameter, interpretable as the visual looming at which $\mathscr{L} = 1$, i.e., the amount of visual looming that fully eradicates the maximum value of ego agent kinematics $\mathscr{K} = 1$ (see Section 1.6).

Compared to the short-term payoff values, the formulation based on access orders in Eq. (9) allows for a simpler expression of priority cost for the vehicle agent of passing first when the pedestrian has priority:

$$\mathscr{R}(\Omega = \text{pass first}) = c \cdot u_{\mathrm{free}} \quad \text{(S10)}$$

with $\mathscr{R} = 0$ otherwise, and with fixed $c = 1.5$ as previously. In the example in Fig. 3C in the main paper, the kinematics-based rewards for the driver agent of passing before a yielding pedestrian are consistently higher than the rewards for the other outcomes (middle panel), but since the pedestrian has the priority in this scenario, the total estimated value for this outcome is shifted downward by the cost in Eq. (S10), making it the lowest-value outcome overall (bottom panel).

We defined three different optional assumptions based on this value estimation scheme:

- **Affordance-based value estimation** (`oVA`), assuming zero acceleration of the other agent after the action prediction interval T_{P}.

- **Affordance-based value estimation with persistent behaviour acceleration** (`oVAa`), assuming that the other agent's acceleration corresponding to behaviour b continues also beyond the action prediction interval[1].
- **Visual looming aversion** (`oVAl`), enabled if $\dot{\theta}_1 < \infty$

Note that `oVA` and `oVAa` are mutually exclusive, whereas `oVAl` can be combined with either of the two.

1.8 Noisy sensory input

To calculate the distance-dependent position noise f_v, we assume that the ego agent estimates the position of the other agent along its line of travel by observing the angle below horizon of the other agent's base [66], and that the sensing of this angle is subject to constant Gaussian noise σ_v [24]. This yields:

$$f_\text{v}[\mathbf{x}(k)] = |D_\text{oth}| \left(1 - \frac{h}{D \tan\!\left(\arctan \frac{h}{D} + \sigma_\text{v}\right)} \right), \tag{S11}$$

where D is the distance between the agents, D_oth is the other agent's longitudinal distance to the crossing point, and h is the eye height over ground of the ego agent, for simplicity here assumed equal to 1.5 m for both pedestrian and driver agents. In reality, humans likely make use of many different optical cues to gauge distances [83]. We considered also the possibility of instead assuming that the ego agent observes, with constant angular noise, the optical size of the other agent, but this does not change the form of the resulting noise in the position estimates.

We defined the following optional, and mutually exclusive, assumptions:

- **Spatial sensory noise** (in cartesian coordinates; `oSNc`), with $\sigma_x(k) = \sigma_\text{s}$, enabled for $\sigma_\text{s} > 0$
- **Angular sensory noise** (in visual coordinates; `oSNv`), with $\sigma_x(k) = f_\text{v}[\mathbf{x}(k)]$, enabled for $\sigma_\text{v} > 0$

1.9 Bayesian perceptual filtering

The Kalman filter estimating position and speed of the other agent based on noisy position observations assumes constant acceleration for the other agent, but includes process noise for the other agent's speed, with standard deviation $\sigma_{\dot{x}}$, which we fixed at 0.1 m/s. We assumed a prior distribution centered at the true initial position of the other agent, at its equilibrium speed, with standard deviations of twice these mean values. These values were subjectively chosen for yielding sensible filter behaviour.

We defined the following optional assumption:

- **Bayesian perceptual filtering** (`oPF`), as described above. If this assumption is not included, the agent is assumed to estimate speed of the other agent by simple between-sample differences in position estimates.

Note that including the `oPF` assumption is only meaningful if the sensory input is actually noisy, i.e., if either `oSNc` or `oSNv` are also included.

1.10 Behaviour probabilities given actions

We defined the following optional assumptions:

- **Value-based behaviour estimation** (`oBEv`), enabled for $\beta_\text{V} > 0$.
- **Observation-based behaviour estimation** (`oBEo`), enabled for $\beta_\text{O} > 0$.

1.11 Behaviour evidence from estimated behaviour value given actions

In past work on behavioural game theory [18,22] the weighted exponential in Eq. (11) is typically applied directly to raw estimated values, but it has also been previously proposed that this type of behaviour estimation can be based on values accumulated over time [84], like we do here. It may be noted that the difference between weighting raw vs accumulated values is smaller in our case than in [84], since our accumulation scheme is constrained such that the accumulated value is a low-pass filtered version of the raw, noisy value.

[1]From a normative point of view, the `oVAa` assumption would seem to make more sense than `oVA`, since if one believes, for example, that the other agent is yielding to oneself, it would seem natural for to believe that this yielding behaviour will not continue only $T_\text{P} = 0.5$ s into the future. However, as described in Section 2, we found the `oVA` assumption to better capture human behaviour.

An important aspect of our formulation is that (in the maximally complex model) the behaviour value $\tilde{V}_{b|a}$ for the other agent in Eq. (14) is contingent on the action a chosen by the ego agent, in line with the so-called Stackelberg formulation of game-theoretic interaction, whereby one agent (in our case, the ego agent), is assumed to move first, and the other agent is assumed to choose its behaviour to optimise value given that this first move is known. This type of formulation has been previously used in models of interaction in road traffic [12, 13, 85] and in other contexts [86]. We define one optional model assumption, only applicable if value-based behaviour estimation (`oBEv`) is assumed in the model:

- **Action-sensitive value-based behaviour estimation** (`oAI`): When disabled, Eq. (14) is modified such that $\tilde{V}_{b|a} = \tilde{V}_{b|a_\varnothing}$, for all a, i.e., the value for the other agent of behaviour b is calculated based on the assumption that the ego agent does not apply any new motor primitive, which in practice means that the ego agent does not attribute different behaviour probabilities to the other agent based on the ego agent's own future action.

1.12 Behaviour evidence from observation of the other agent

The specific form of Eq. (15) is chosen because if we fix $\beta_\text{O} = 1$ (which we do in all our simulations), then if $T_\text{Of} \to \infty$ (no forgetting), $\Delta t = T_\text{O1}$ (observation update duration equal to model time step) and $\beta_\text{V} = 0$ (`oBEv` disabled) we get:

$$\begin{aligned}
P_{b|a}(k) &= \{\text{Eq. (11)}\} = \mathscr{S}\left[\{A_{b'|a}\}_{b'}, b, k\right] \\
&= \{\beta_\text{V} = 0\} = \mathscr{S}\left[\{\hat{A}_{\text{O},b'}\}_{b'}, b, k\right] \\
&= \frac{e^{\hat{A}_{\text{O},b}(k)}}{\sum_{b'} e^{\hat{A}_{\text{O},b'}(k)}} \\
&= \{\text{Eq. (15)}, T_\text{Of} \to \infty, T_\text{O1} = \Delta t\} \\
&= \frac{e^{\hat{A}_{\text{O},b}(k-1)} p[\tilde{\mathbf{x}}(k)|\tilde{\mathbf{x}}(k-1), b]}{\sum_{b'} e^{\hat{A}_{\text{O},b'}(k-1)} p[\tilde{\mathbf{x}}(k)|\tilde{\mathbf{x}}(k-1), b']} \\
&= \frac{\frac{e^{\hat{A}_{\text{O},b}(k-1)}}{\sum_{b''} e^{\hat{A}_{\text{O},b''}(k-1)}} p[\tilde{\mathbf{x}}(k)|\tilde{\mathbf{x}}(k-1), b]}{\sum_{b'} \frac{e^{\hat{A}_{\text{O},b'}(k-1)}}{\sum_{b''} e^{\hat{A}_{\text{O},b''}(k-1)}} p[\tilde{\mathbf{x}}(k)|\tilde{\mathbf{x}}(k-1), b']} \\
&= \frac{P_{b|a}(k-1) \cdot p[\tilde{\mathbf{x}}(k)|\tilde{\mathbf{x}}(k-1), b]}{\sum_{b'} P_{b'|a}(k-1) \cdot p[\tilde{\mathbf{x}}(k)|\tilde{\mathbf{x}}(k-1), b']},
\end{aligned}$$

which is a standard Bayesian update.

In other words, taken together, Eq. (11) through Eq. (15) provide a general framework for behaviour estimation, of which pure Bayesian observation-based inference without forgetting is one special case (for $\beta_\text{V} = 0$, $T_\text{Of} \to \infty$), and pure value-based inference is another (for $\beta_\text{O} = 0$). Note further that since all $A_{\text{O},b}$ are initialised to zero, if both types of behaviour estimation (both `oBEv` and `oBEo`) are assumed, early $P_{b|a}$ estimates will be determined by early estimated behaviour value estimates $\hat{V}_{b|a}$ (or $\tilde{V}_{b|a}$, if `oEA` is not assumed), and will shift over time toward the behaviours suggested by the observations. In other words, the initial value estimates determine a prior for the Bayesian observation, but note that this prior can then also vary over time, if the estimated behaviour values for the other agent change as the world state changes.

It may be noted that the model of action understanding by Baker et al. [19] also integrates concepts of value for the other agent with observation of that agent's states. However, in their case the value of a state for the other agent—given the intended overall behaviour of that agent, i.e., its goal—is used to estimate $p[\tilde{\mathbf{x}}(k)|\tilde{\mathbf{x}}(k-1), b]$. Our model could be extended in this direction, but to avoid an overly complex model we took the simpler approach described in Methods, since in our case each behaviour b can be naturally mapped to a specific acceleration in a given situation. In our `oBEv` models, the value estimation instead happens at a higher level, to judge the value of the overall behaviour for the other agent. This aspect is missing in the Baker et al. model, but is important here, not least to make possible the `oAI` assumption.

With regards to the T_Of term in Eq. (15), we are not aware of any previous Bayesian models of action understanding incorporating forgetting, but assumptions of evidence leakage has been common in evidence accumulation models of decision-making [50, 51], to which Eq. (15) is analogous (cf., e.g., [87]).

There is redundancy between the parameters σ_O and T_O1, whereby increasing one and decreasing the other can leave the model behaviour relatively unchanged; for this reason we fixed $T_\text{O1} = 0.05$ s and left only σ_O as a free model parameter.

Table S1: Optional model assumptions.

	Assumption	Free parameters
`oAI`	Action-sensitive value-based behaviour estimation	-
`oAN`	Value noise	σ_V
`oBEc`	Estimation of constant-speed behaviour	-
`oBEo`	Observation-based behaviour estimation	T_Of, σ_O
`oBEv`	Value-based behaviour estimation	β_V
`oEA`	Evidence accumulation	T, $\Delta V_\mathrm{th,rel}$
`oPF`	Bayesian perceptual filtering	-
`oSNc`	Spatial sensory noise	σ_s
`oSNv`	Angular sensory noise	σ_v
`oVA`	Affordance-based value estimation	T_δ
`oVAa`	Affordance-based value estimation with persistent behaviour acceleration	T_δ
`oVAl`	Visual looming aversion	$\dot{\theta}_1$

1.13 Summary of model assumptions

Table S1 lists all of the optional model assumptions introduced above, together with the free parameters associated with each assumption. Note that if neither `oVA` nor `oVAa` are included, simple short-term payoff value estimation is assumed instead, with free parameters k_c and k_sc.

In these supplementary materials we will refer to model variants by combining the abbreviations for the included optional assumptions. For example, the most successful deterministic model `oVAoBEvoAI` (shown for example in Fig. 3B in the main paper) assumes affordance-based value estimation and action-sensitive value-based behaviour estimation, and the most successful model overall `oVAoBEvoAIoSNvoEAoPF` (shown for example in Fig. 3E and Fig. 4 in the main paper) also assumes angular sensory noise, evidence accumulation, and Bayesian perceptual filtering.

2 Model selection – full results

2.1 Deterministic tests

We tested all 36 model variants obtained from combining optional assumptions from one of:

{ <none>, `oVA`, `oVAa`, `oVAoVAl`, `oVAaoVAl`, `oVAaoBEc` }

with one of:

{ <none>, `oBEo`, `oBEv`, `oBEooBEv`, `oBEvoAI`, `oBEooBEvoAI` },

where, as mentioned in Section 1.13, the model variant with no optional assumptions is the base model with short-term payoff value estimation. Overall, the first set of six assumption combinations above define the model variant's value estimation scheme, and the second set of six assumption combinations define the model variant's behaviour estimation scheme. The exception is the inclusion of the `oBEc` assumption in the first set, placed there for simplicity, since we were only interested in testing it in combination with `oVAa`, as an alternative to `oVAl` for making the `oVAa` model less prone to collisions (which can easily arise if the `oVAa` model becomes overly certain that the other agent is passing first or second).

2.1.1 Models achieving individual phenomena

Priority assertion As can be seen in Fig. S5, metric values $\bar{v}_\mathrm{v}/v_\mathrm{v,free} > 1$ were observed for model variants with value estimation schemes `oVA` and `oVAoVAl`, but only if combined with `oBEvoAI`, with or without `oBEo`. For value estimation scheme `oVAoVAl`, all behaviour estimation schemes could yield priority assertion. Simulation examples of priority assertion can be seen in the middle and bottom plots in Fig. S8 and the bottom plot in Fig. S9.

Table S2: Searched ranges for free parameters of the deterministic model assumptions. In addition to the motivations mentioned below, these ranges were also in some cases extended based on the results of smaller-scale initial searches, when there were indications that a range of parameterizations achieving the empirical phenomena could be completely covered by the search by extending it.

Parameter	Range	Motivation
k_c	[0.2, 2]	Initial tests of a few close encounter scenarios with the other agent (in this case the vehicle) at constant speed, identifying k_c at which the model became collision-free; then selecting a range of an order of magnitude around that value.
k_{sc}	[0.02, 0.2]	As for k_c.
T_{Of}	[0.5, 10] s	Plausible values for how quickly old observations of the other agent's behaviour become irrelevant and can be discarded.
T_δ	[10, 100] s	Plausible values for how quickly the perceived value of future reward (in this case, future progress in traffic) halves with delays.
β_V	[1, 200]	Interpretable as the probability $P_\dagger$ of choosing a behaviour of maximally negative squashed value over an alternative with zero squashed value, ranging from $P_\dagger = 10^{-87}$ (for $\beta_V = 200$), to $P_\dagger = 0.27$ (for $\beta_V = 1$).
σ_O	[0.02, 2] m	Plausible values for position uncertainty in the agent's ability to predict the other agent's position for a given behaviour.
$\dot{\theta}_1$	[0.001, 0.1] rad/s	Based on human perceptual limitations for detecting visual looming in the range 0.001–0.003 rad/s [88] and values of 0.02 rad/s being clearly aversive to drivers [89].

Table S3: Base scenario kinematics, metrics, and metric thresholds for each of the five main targeted interaction phenomena. TTA = Time to arrival at conflict space. At the kerb = With a distance margin of $D_s = 1$ m to the conflict space. When metrics include averages, these are measured from the start of the simulation until the active agent reaches halfway to the conflict space.

Phenomenon (active agent; priority)	Base scenario	Motivation for kinematics	Behaviour metric and values indicative of phenomenon being exhibited (definition)	Retention threshold
Short-stopping (driver; does not have priority)	Car initially at $v_{\text{v,free}}$ with 4 s TTA. Pedestrian stationary at the kerb throughout.	Driver can comfortably yield to the pedestrian with deceleration < 2 m/s^2.	$\overline{d - d_{\text{stop}}} > 0$ m/s^2 (average excess of momentary deceleration d compared to the momentary deceleration d_{stop} required for stopping with a margin of $D_s = 1$ m before the conflict space)	> 0.5 m/s^2
Priority assertion (driver; has priority)	Car initially at $v_{\text{v,free}}$ with 2 s TTA. Pedestrian stationary at the kerb throughout.	Pedestrian cannot cross at $v_{\text{p,free}}$ without the car yielding, but the driver can yield to the pedestrian with deceleration $<$ 4 m/s^2.	$\bar{v}_{\text{v}}/v_{\text{v,free}} > 1$ (average of car speed divided by its equilibrium speed)	> 1.005
Yield acceptance hesitation (pedestrian; has priority)	Car initially at $v_{\text{v,free}}$ with 3 s TTA. Pedestrian initially at $v_{\text{p,free}}$ with 3 s TTA.	Pedestrian has some time for deliberation before reaching conflict space, agents initially on collision course, but the car can yield with deceleration < 4 m/s^2.	$\bar{v}_{\text{p}}/v_{\text{p,free}} < 1$ (average of pedestrian speed divided by its equilibrium speed)	< 0.95
Early yield acceptance (pedestrian; has priority)	Car initially at $v_{\text{v,free}}$ with 2 s TTA, applying constant deceleration to yield with distance margin $D_s = 1$ m to conflict space. Pedestrian initially stationary at kerb.	If pedestrian immediately starts walking at $v_{\text{p,free}}$ and car does not yield, the two agents collide, but the required yielding deceleration of the car is < 4 m/s^2.	$v_{\text{v}}(t_{\text{cross}}) > 0$ m/s (car speed when the pedestrian agent first starts moving)	> 0.5 m/s
Gap acceptance hesitation (pedestrian; does not have priority)	Car initially at $v_{\text{v,free}}$ with 8 s TTA. Pedestrian initially at $v_{\text{p,free}}$ with 3 s TTA.	Pedestrian has some time for deliberation before reaching conflict space, and if keeping constant speed throughout will exit conflict space with 1 s extra margin over the distance and time margins defined in Section 1.4.	$\bar{v}_{\text{p}}/v_{\text{p,free}} < 1$ (average of pedestrian speed divided by its equilibrium speed)	< 0.95

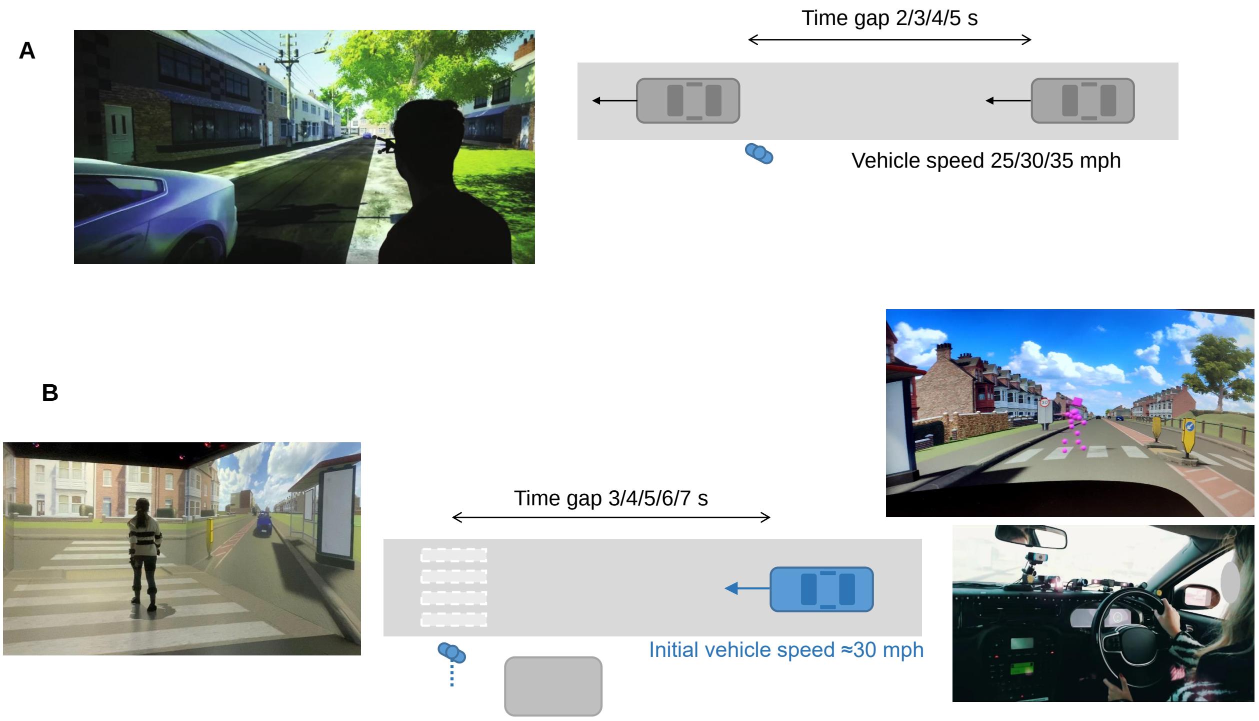

Figure S4: Schematic illustrations of the two controlled experiments. Note that the bird's eye view illustrations are not to scale. (A) Experiment 1, where a participant in a pedestrian simulator was waiting at the kerb and decided if and when to cross between two vehicles, the second of which sometimes yielded to the pedestrian. (B) Experiment 2, where a participant in the pedestrian simulator was cued to step up to the kerb (with or without a zebra crossing) from behind a vision obstruction, at which point they could see and interact with another participant in a driving simulator.

Short-stopping Fig. 1B in the main paper and Fig. S5 show that most model variants were capable of exhibiting at least small positive values of the $\overline{d - d_{\text{stop}}}$ excess deceleration metric. However, on closer scrutiny of the model simulations, we noticed that some model variants could show small initial excess deceleration, without later actually stopping short of the conflict space. For an example, see the bottom plot in Fig. S7.

For this reason, we identified model variants and parameterizations for which $\overline{d - d_{\text{stop}}} > 0.5$ m/s^2 for at least one of the kinematic variants of the scenario, and subjected these to further analysis: For each model variant meeting this excess deceleration criterion, we randomly selected up to 1,000 of the parameterizations meeting the criterion, and checked whether they actually stopped short: We simulated these model parameterizations until the vehicle had decelerated to a speed of 0.5 m/s, and measured its distance D_{stop} to the conflict space at this time, accepting parameterizations for which D_{stop} was larger than the vehicle length (4.2 m) as indicative of short-stopping [29]. As can be seen in Fig. S6, this type of actual short-stopping was observed only for model variants with value estimation schemes `oVA`, `oVAoVAl`, or `oVAaoVAl`, and only if combined with some form of behaviour estimation, with particularly pronounced short-stopping for models including `oBEvoAI`. For examples, see the middle and bottom plots in Fig. S8, and the bottom plot in Fig. S9.

For purposes of retaining model parameterizations for the later stochastic test, we considered a model parameterization as achieving short-stopping if its model variant passed the test based on final stopping distance, and if the parameterization itself passed the test based on initial excess deceleration. It should be noted that the model variants which failed the distance-based test all had less than 1,000 parameterizations passing the initial deceleration-based test, i.e., these model variants were all exhaustively tested before rejecting them.

Gap acceptance hesitation As can be seen in Fig. 1B and Fig. S5, we observed metric values $\bar{v}_{\text{p}}/v_{\text{p,free}} < 1$ for models with behaviour estimation scheme `oBEo`, across all value estimation schemes, and also in some cases where `oBEv` was included.

Yield acceptance hesitation As can be seen in Fig. 1B in the main paper, in Fig. S5, and in many of the simulation examples in Figs. S7–S9, all tested model variants could achieve yield acceptance hesitation for at least some model parameterizations.

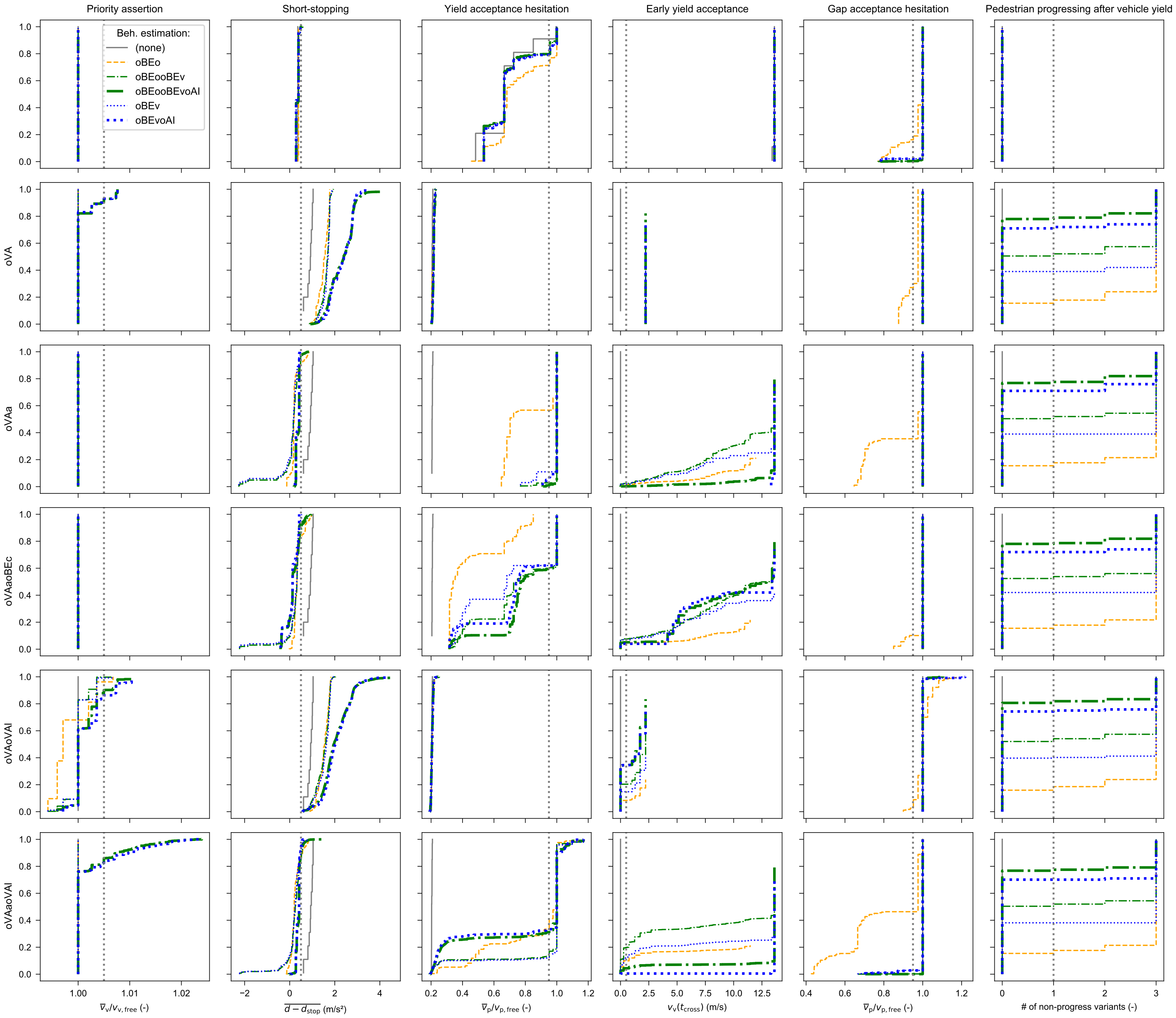

Figure S5: Cumulative distributions of the quantitative metric values obtained across all tested parameterizations of the deterministic model variants. The vertical dotted lines show the quantitative threshold applied to each metric when deciding which parameterizations to retain for further analysis. Each row of panels shows results for the model variant listed to the left of the row (the top row is the base model), combined with the different behaviour estimation schemes indicated by the line styles (see the plot legend). The first five columns correspond to the five main targeted phenomena illustrated in Fig. 1A in the main paper (although shown here in a different order), and the metrics shown on the x axes are the same as in that figure (these metrics are defined in Table S3). The rightmost column corresponds to an additional criterion for retention of parameterizations, requiring that the pedestrian agent in the early yield acceptance scenario begins crossing after the vehicle has come to a full stop, if not earlier.

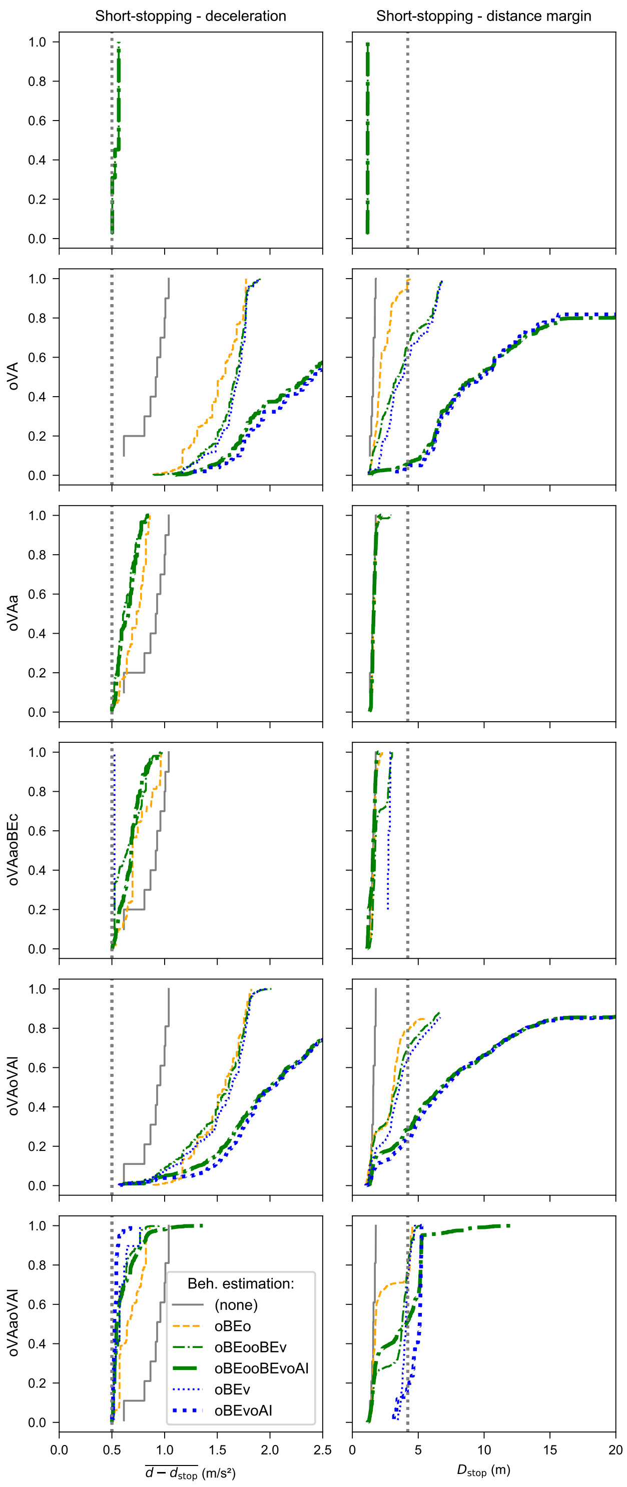

Figure S6: Cumulative distributions of the quantitative metric values obtained in the follow-up deterministic tests of model short-stopping behaviour. The left column shows the same results for short-stopping as in Fig. S5, but here only including those parameterizations which achieved short-stopping in the sense of exhibiting some exaggerated early deceleration. The right column shows the results from testing these parameterizations to see if they also actually stopped short of the pedestrian. See Fig. S5 for further explanations of how to read this figure.

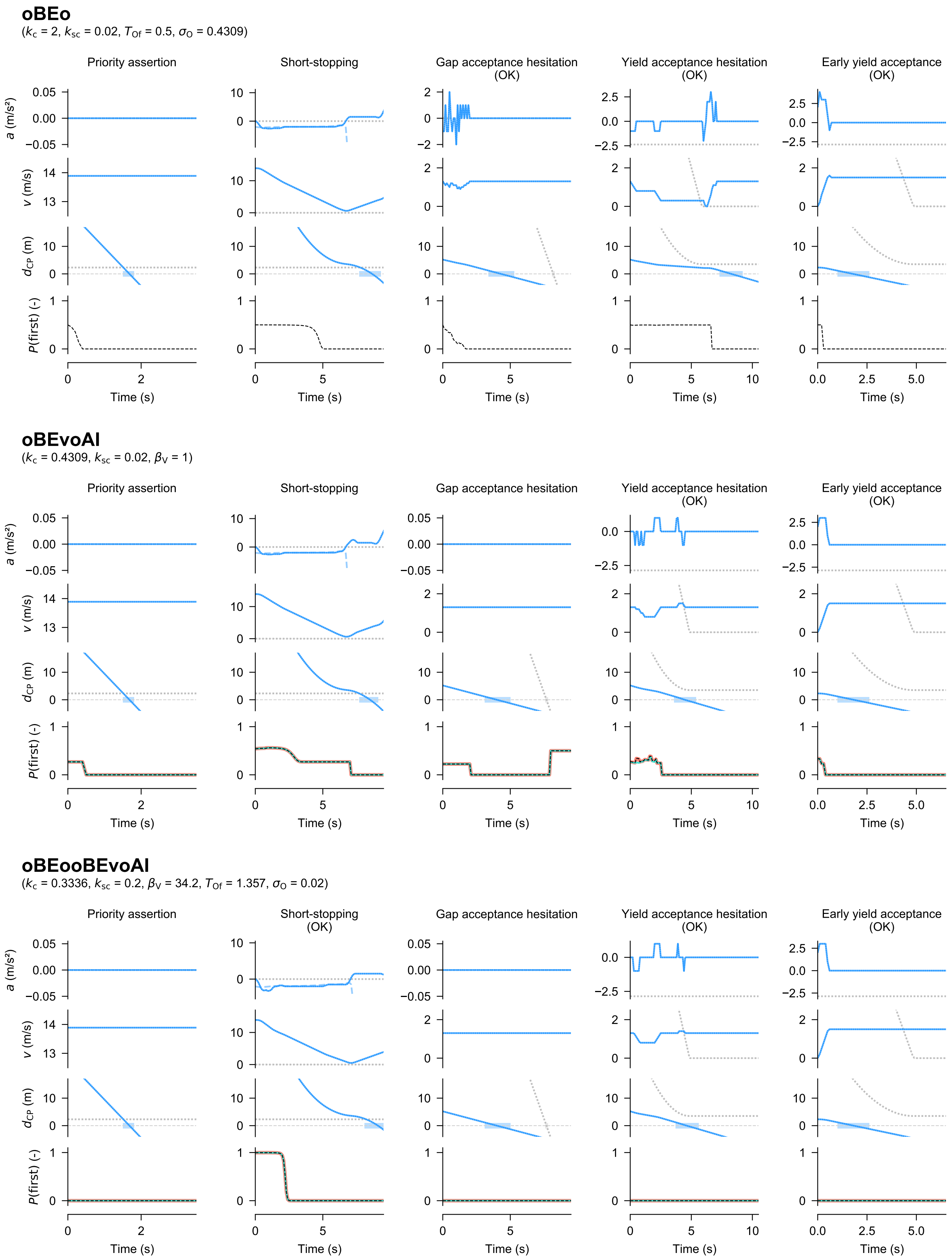

Figure S7: Example deterministic time-series simulations for model variants with short-term payoff value estimation, for the same scenarios as shown in Fig. 1A in the main paper, with blue and grey lines again referring to modelled and scripted agent, and with "OK" indicating meeting the criteria for retention defined in Table S3 (for short-stopping, this is the early deceleration criterion, not the additional final distance criterion described in Section 2.1.1). The bottom panels show the modelled agent's estimated probability of the other agent adopting the behaviour of passing first. For models assuming `oBEvoAI` light blue and red lines show these probabilities given that the modelled agent takes action to increase or decrease its speed, respectively; note that these probabilities may overlap or only differ very slightly.

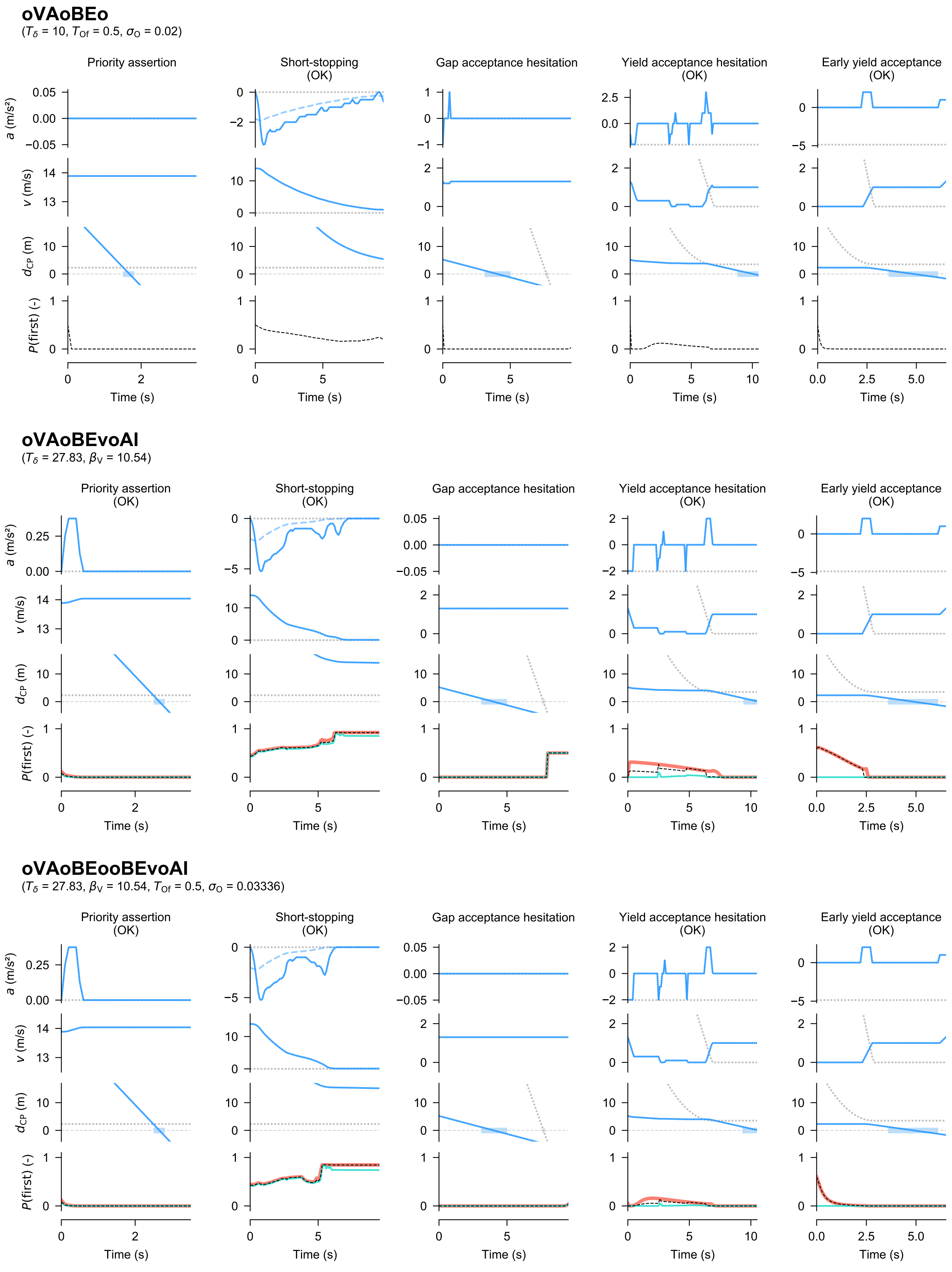

Figure S8: Example deterministic time-series simulations for model variants with affordance-based value estimation (oVA). See Fig. S7 for explanations.

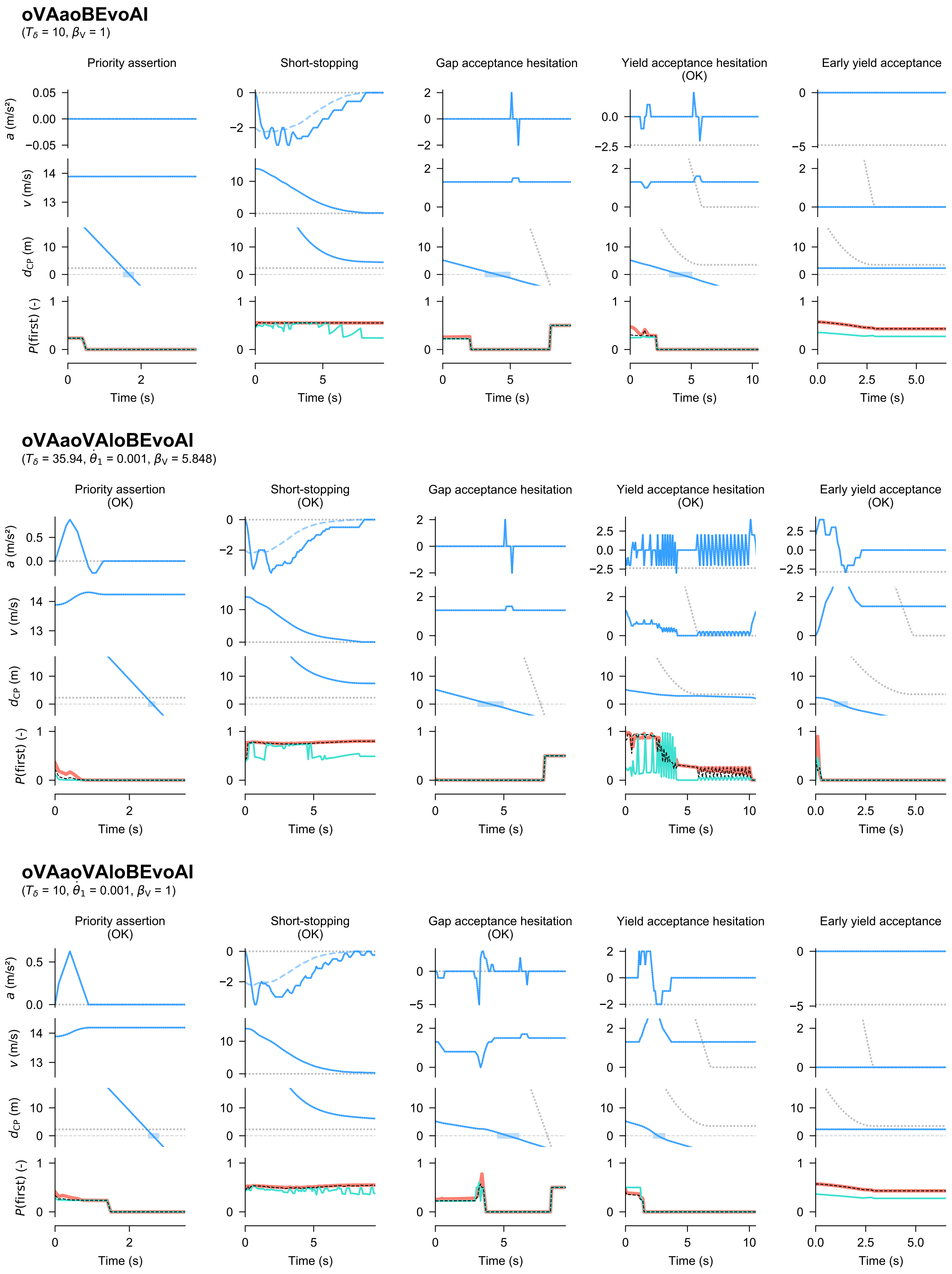

Figure S9: Example deterministic time-series simulations for model variants with affordance-based value estimation with persistent behaviour acceleration (`oVAa`). See Fig. S7 for explanations.

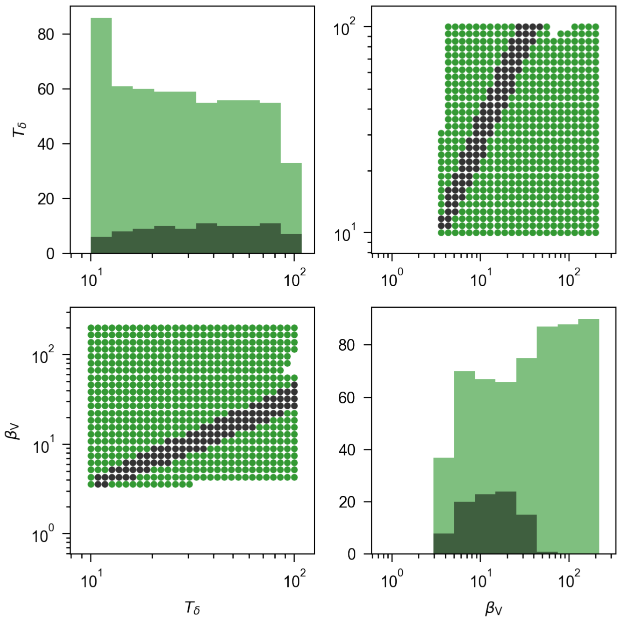

Figure S10: Parameters for model `oVAoBEvoAI` meeting the criteria for retention from the deterministic tests. In this figure (and others like it below), each row and column refers to one free model parameter, and the histograms on the main diagonal show the one-dimensional marginal distributions for each parameter (in these panels the y axis shows the number of retained parameterizations), whereas the other panels visualise the two-dimensional marginal distributions for each combination of two parameters, with each retained parameterization represented by one dot (with minor jitter introduced for models with more than two parameters, to improve visibility). Deterministic model parameterizations achieving three of the five targeted phenomena are shown in green, whereas parameterizations achieving four (all except gap acceptance hesitation) are shown in black. Note that the results in this figure are from the expanded grid search for this model variant, with 30 values per free parameter.

Early yield acceptance As can be seen in Fig. 1B in the main paper and in Fig. S5, this was observed for all model variants, except for those model variants which assumed affordance-based value estimation, but no behaviour estimation at all. The topmost plot in Fig. S9 shows an example of a model variant failing to exhibit early yield acceptance, with the pedestrian instead remaining at zero speed, due to high uncertainty about the car's behaviour.

2.1.2 Model variants achieving multiple phenomena

In the deterministic tests described above, the largest number of phenomena that was achieved by any parameterization of any of the model variants was four, and in all of these cases the single phenomenon not achieved was gap acceptance hesitation. For example model parameterizations of this nature, see middle and bottom of Fig. S8 and middle of Fig. S9. The model variants and parameterizations which achieved gap acceptance hesitation, consistently achieved fewer of the other phenomena; for examples see top of Fig. S7 and bottom of Fig. S9.

Six out of the 36 tested deterministic model variants had model parameterizations achieving the maximum of four out five phenomena, specifically all combinations of one of the value estimation schemes { `oVA`, `oVAoVAl`, `oVAaoVAl` } with one of the two behaviour estimation schemes { `oBEvoAI`, `oBEooBEvoAI` }. Figs. S10–S12 show, for three of these six models (`oVAoBEvoAI`, `oVAoVAloBEooBEvoAI`, and `oVAaoVAloBEooBEvoAI`), the parameterizations achieving three and four of the phenomena, as well as the added criterion also shown in Fig. S5, requiring that the pedestrian in the early yield acceptance does not remain stationary even after the vehicle has come to a full stop. As can be seen in Fig. S5, the models with affordance-based value estimation combined with some form of behaviour estimation scheme can "get stuck" in this way. This happens for parameterizations with high uncertainty in their beliefs; see the top and bottom right of Fig. S9 for two examples.

The minimally complex model variant which managed to account for all of the targeted phenomena except gap acceptance hesitation was thus `oVAoBEvoAI`, i.e., the model variant assuming affordance-based value estimation and action-sensitive value-based behaviour estimation. Fig. S10 shows that there is a narrow tradeoff between the T_δ and β_V parameters for which this model achieves four of the five phenomena. Adding looming aversion (`oVAl`) or observation-based behaviour estimation (`oBEo`) did not increase the model's capacity

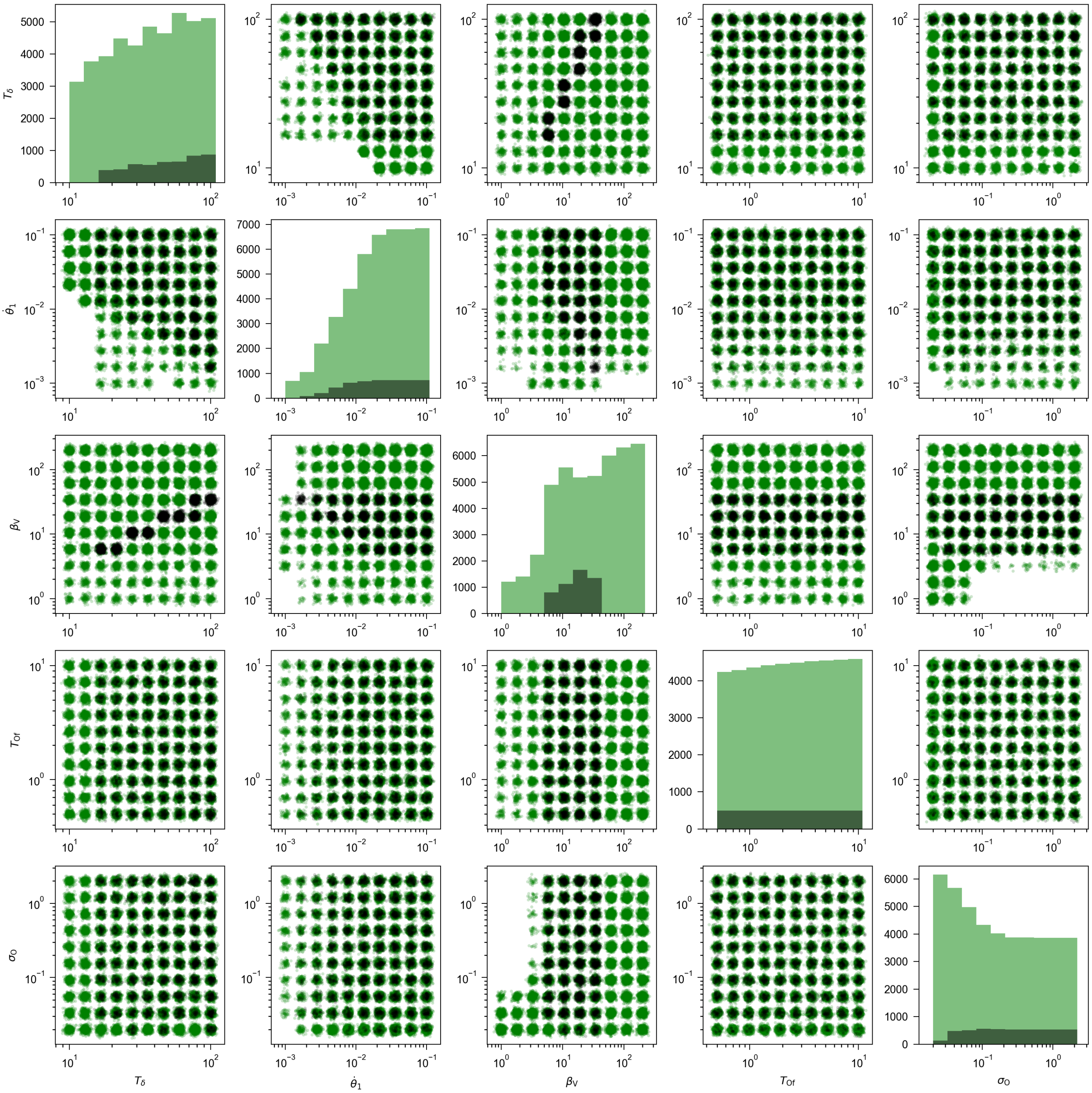

Figure S11: Retained parameterizations for deterministic model `oVAoVAloBEooBEvoAI`. See Fig. S10 for explanations.

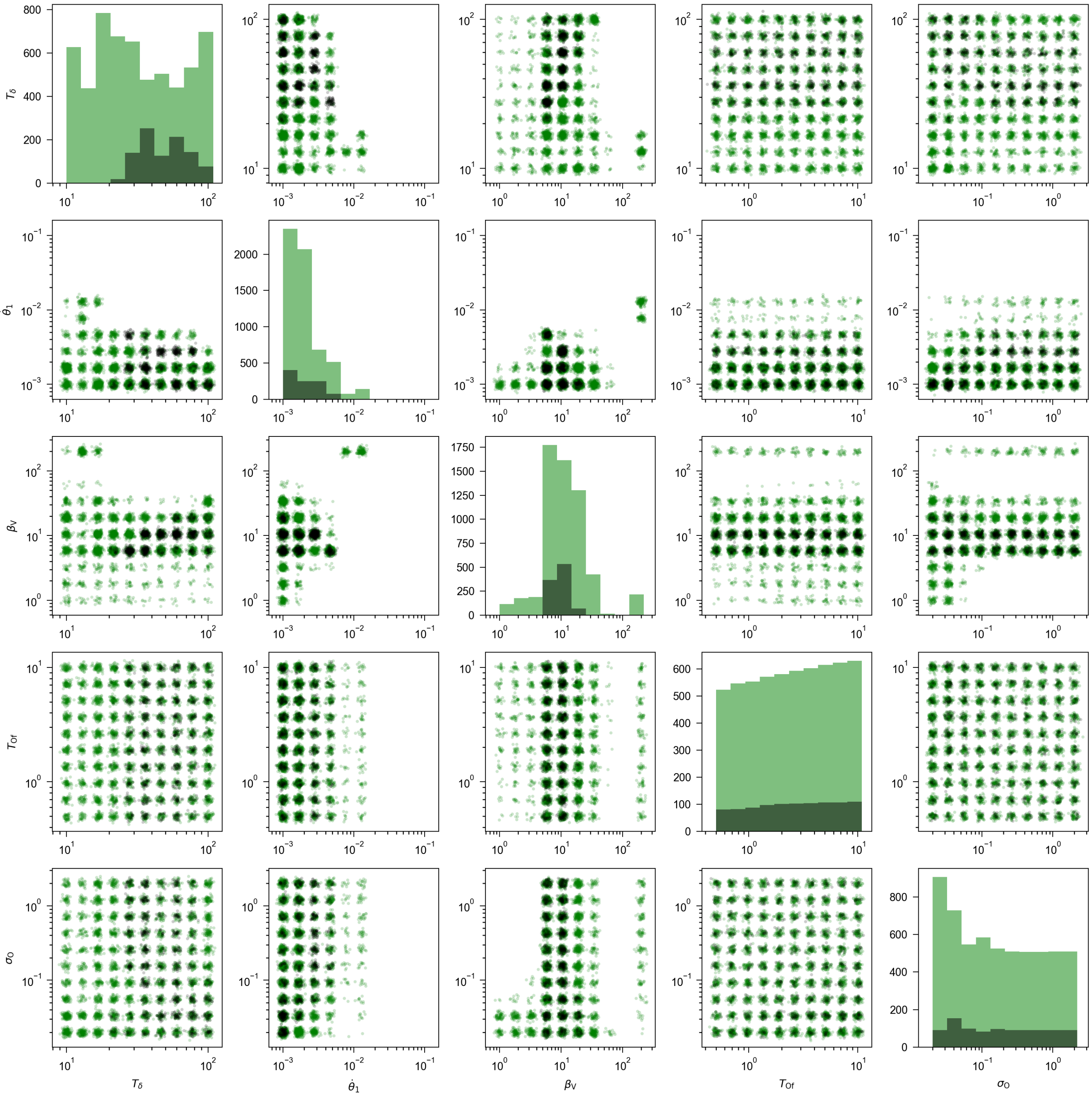

Figure S12: Retained parameterizations for deterministic model `oVAaoVAloBEooBEvoAI`. See Fig. S10 for explanations

Table S4: Searched ranges for free parameters of the stochastic model assumptions. If `oPF` is enabled, $C = 1$, otherwise $C = 0.001$. These ranges were to some extent based on a priori plausibility (in particular for T, σ_s, and σ_v), but mainly on preliminary model tests, identifying values for which the model gave subjectively reasonable behaviour, defining ranges around these values, and then (as also mentioned for the deterministic tests) further extending the ranges in some cases if small-scale parameter searches suggested that this would permit more complete coverage of parameterizations achieving the targeted phenomena. Note the multiplication of sensory noise magnitudes by a factor 0.001 when `oPF` is not included; this is because the `oPF` filtering very quickly reduces the perceptual uncertainty, such that the ranges of noise intensity at which the model exhibits gap acceptance hesitation is very different for model variants with and without `oPF`.

Parameter	Range
T	[0.1, 0.5] s
$\Delta V_{\mathrm{th,rel}}$	[0.001, 0.1]
σ_V	[0.001, 1]
σ_s	$C \times [0.5, 500]$ m
σ_v	$C \times [0.005, 5]$ rad

to reproduce the targeted behaviour phenomena, and Fig. S11 shows that the free parameters added by these additional assumptions did not affect the model's capability to reproduce the phenomena already achieved by the `oVAoBEvoAI` model, as long as looming aversion was not made too strong (too low $\dot{\theta}_1$). It is also interesting to note that the version of the affordance-based value estimation with persistent behaviour acceleration (`oVAa`) was only successful if paired with looming aversion, and as can be seen in Fig. S12 only for low values for $\dot{\theta}_1$, i.e., strong looming aversion, which cancels out much of the effect of the acceleration-persistence (because visual looming only depends on current position and speed, not acceleration), thus in practice making the `oVAaoVAl` value estimation scheme quite similar to `oVA`.

2.1.3 Additional tests of negative results

To further verify the most important negative findings mentioned above, we reran the deterministic tests with larger search grids of 30 values per parameter for (1) the base model and model `oBEvoAI`, confirming our conclusions that these models are only capable of basic collision-averse behaviour, and not priority assertion, short-stopping, or gap acceptance hesitation, and (2) the maximally successful deterministic model `oVAoBEVoAI`, confirming that this model is not capable of gap acceptance hesitation.

2.2 Stochastic tests

2.2.1 Simple model variants, with minimal deterministic assumptions

In our first tests of stochastic model variants, starting from the simplest deterministic model with affordance-based value estimation, we fixed the only parameter of that model $T_\delta = 40$ s, based on the deterministic test results (Fig. S10). We then tested all ten possible combinations of one of:

{ `oVA`, `oVAoEA` }

(i.e., either not assuming or assuming evidence accumulation) together with one of:

{ `oAN`, `oSNc`, `oSNv`, `oSNcoPF`, `oSNvoPF` }

(i.e., assuming either value noise or sensory noise, and if the latter then either in spatial or angular form, and either with or without Bayesian perceptual filtering).

As can be seen in Fig. S13, collisions did arise for some parameterizations of all model variants except `oVAoAN`. As for gap acceptance hesitation, all model variants exhibited at least some minimal degree of pedestrian slowing before crossing. However, when further investigating the behaviour of the models with value noise (`oAN`) it was discovered that these model variants had a general tendency to slow down from the initial (theoretical, derived for the deterministic case) equilibrium speed of the pedestrian agent, even in the absence of an approaching vehicle. For this reason, we first identified all model variants and parameterizations which achieved (i) quantitative gap acceptance hesitation of $\bar{v}_p/v_{p,\mathrm{free}} < 0.95$ (same threshold as in the deterministic tests) in at least four of the five repeated stochastic simulations, where (ii) the pedestrian entered the conflict space in all five repetitions (to exclude parameterizations with a tendency to get stuck), as well as (iii) collision-free behaviour in all repetitions of the three interactive scenarios. Six model variants were identified with parameterizations that met these criteria:

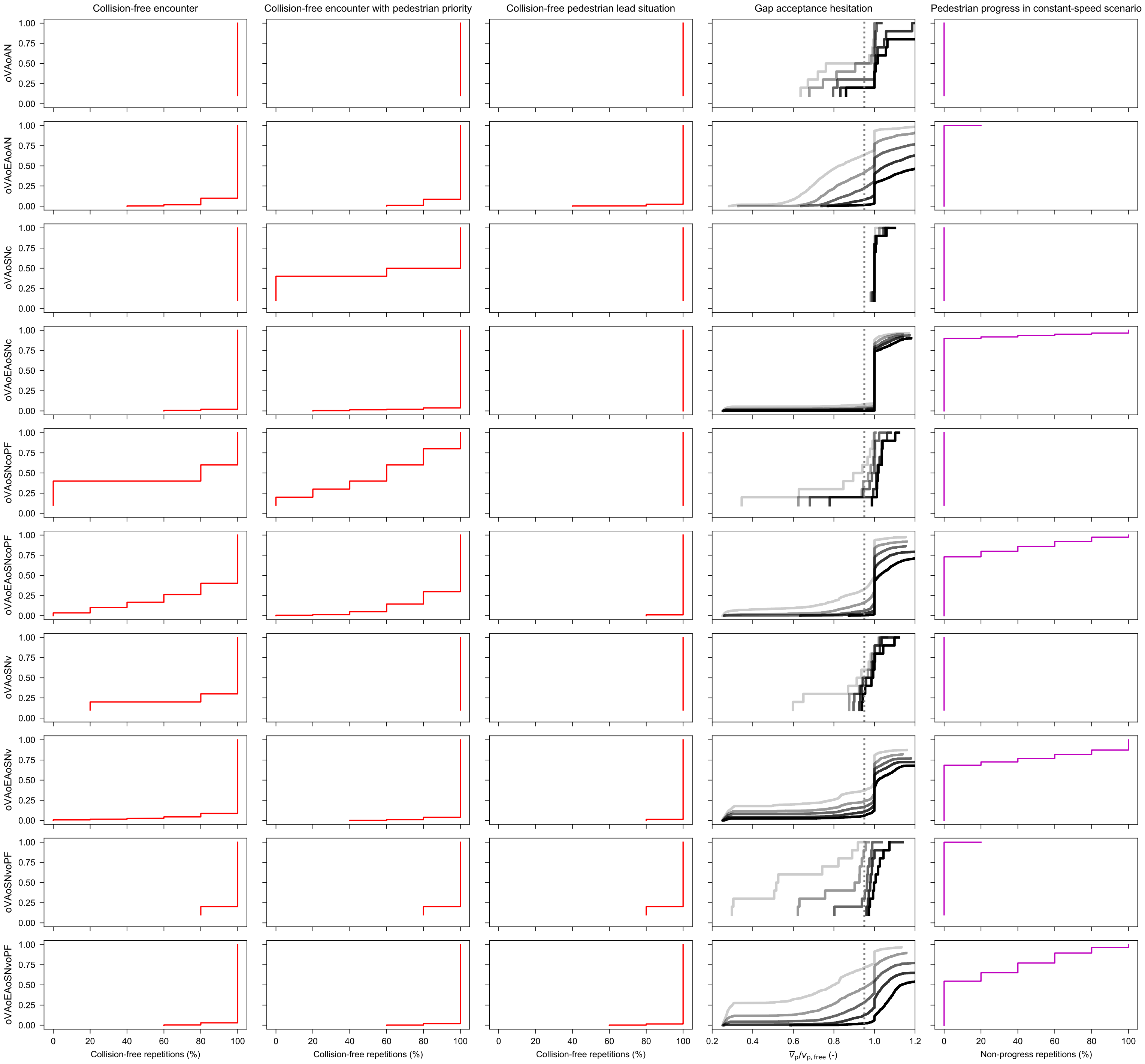

Figure S13: Cumulative distributions of quantitative metric values obtained across all tested parameterizations of the simple stochastic model variants. The first three columns of panels show the frequency of collisions in the five repetitions of each two-agent scenario. The five lines in the fourth column represent the cumulative distributions of the gap acceptance hesitation metric for different number of stochastic simulation repetitions. The line in lightest grey show the fraction of parameterizations with at least one simulation achieving a metric value at or below the x value, the next line show those with at least two simulations at or below the x value, such that the darkest line shows the fraction of parameterizations with all parameterizations achieving a metric value at or below the x value. The rightmost column is the same as the rightmost column in Fig. S5, but across the five repetitions of each scenario instead of across three kinematic variations.

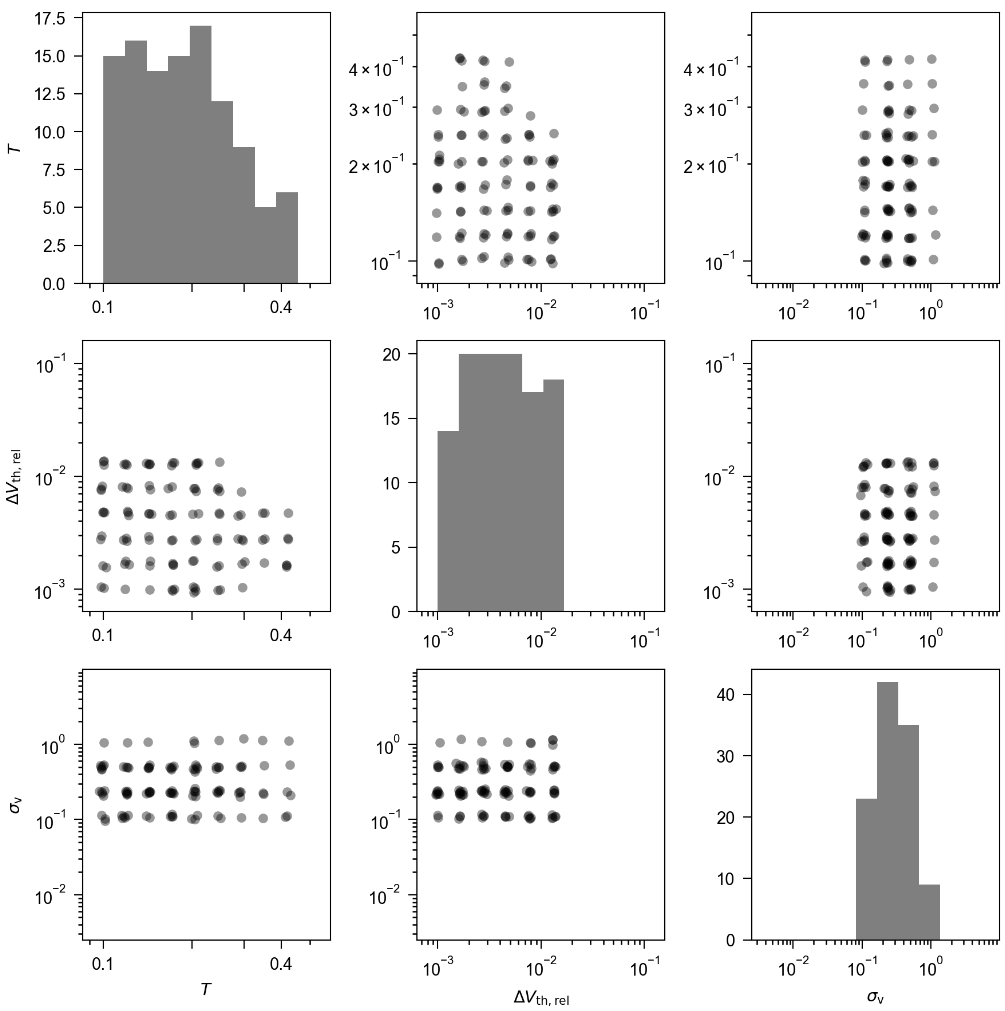

Figure S14: parameterizations retained for the simple stochastic model variant `oVAoEAoSNv`. See Fig. S11 for further explanations of how to read this figure.

{ `oVAoAN`, `oVAoSNv`, `oVAoEAoAN`, `oVAoEAoSNc`, `oVAoEAoSNv`, `oVAoEAoSNvoPF`}

For each identified model parameterization of each of these model variants, we simulated 50 additional repetitions of the gap acceptance hesitation scenario, as well as 50 repetitions of a scenario without an approaching vehicle, and calculated 95 % confidence intervals for the mean of the $\bar{v}_p/v_{p,\text{free}}$ metric in the two scenarios. For the two model variants including `oAN`, these confidence intervals were overlapping for all model parameterizations, indicating that the model did not slow down more in the presence of the approaching vehicle than otherwise, and thus did not exhibit gap acceptance hesitation. In contrast, for all of the other four model variants (i.e., `oVAoSNv`, `oVAoEAoSNc`, `oVAoEAoSNv`, and `oVAoEAoSNvoPF`), mean $\bar{v}_p/v_{p,\text{free}}$ was lower when the approaching vehicle was present, with non-overlapping confidence intervals for 99-100 % of the tested parameterizations, thus indicating robust gap acceptance hesitation. Fig. S14 and Fig. S15 shows the distribution of parameterizations meeting all of the criteria for the `oVAoEAoSNv` and `oVAoEAoSNvoPF` models respectively. It can be seen that these distributions are quite similar, except for the noise magnitude parameter σ_v, as mentioned earlier.

2.2.2 Combining with more complex deterministic assumptions

Out of the $6 \times 4 = 24$ more complex stochastic model variants tested, for all except one (`oVAaoVAloBEvoAIoEAoSNc`), parameterizations were identified which met all of the retention criteria, i.e., almost all of these more complex stochastic model variants were capable of gap acceptance hesitation and collision-free interaction. Fig. S16 shows the obtained results for a selection of the 24 tested model variants. The four first rows in the figure show the four different stochastic assumption combinations retained from the simple stochastic tests, together with the maximally successful deterministic model `oVAoBEvoAI`, suggesting that the capacity for gap acceptance of the simpler stochastic model variants was somewhat reduced by the addition of `oBEvoAI` (since the simpler stochastic model parameterizations were all retained based on a $4/5 = 80$ % rate of gap acceptance hesitation, which is now only achieved by around $25 - 75$ % of the tested parameterizations of the more complex models).

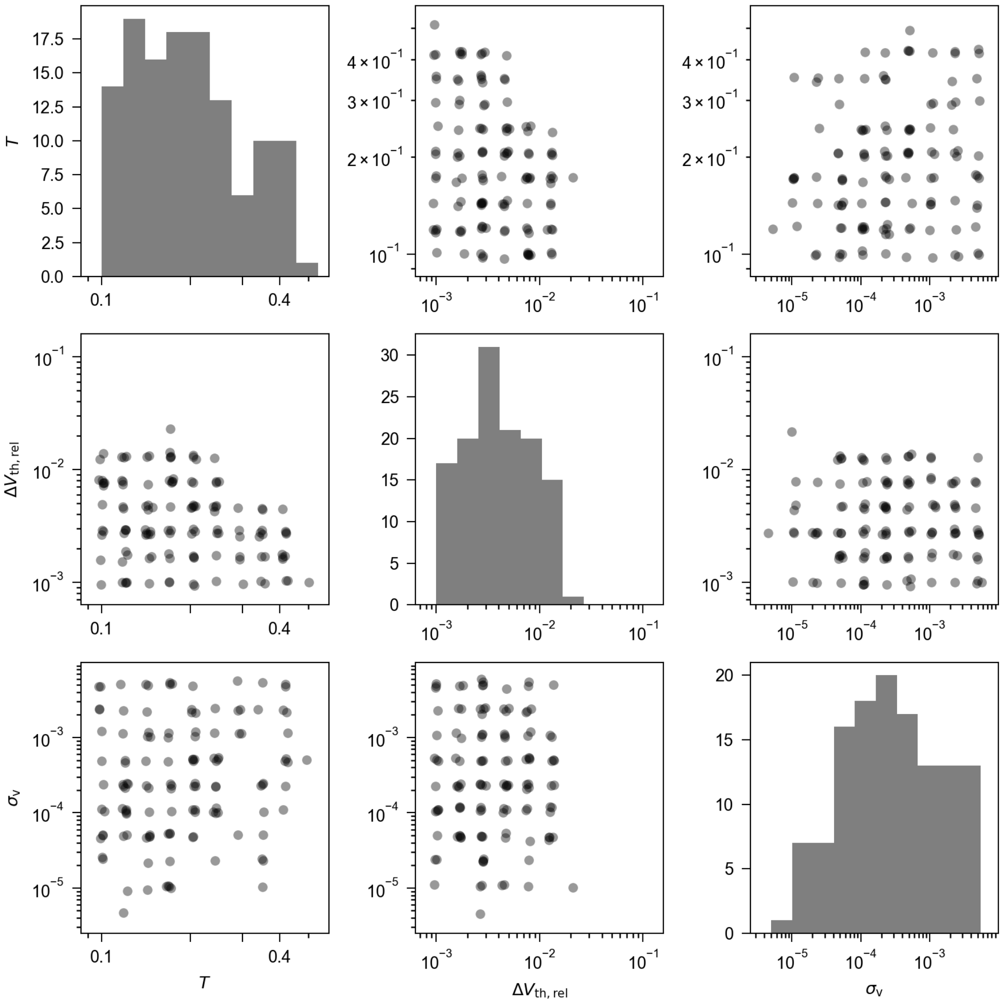

Figure S15: parameterizations retained for the simple stochastic model variant `oVAoEAoSNvoPF`. See Fig. S11 for a further explanation of how to read this figure.

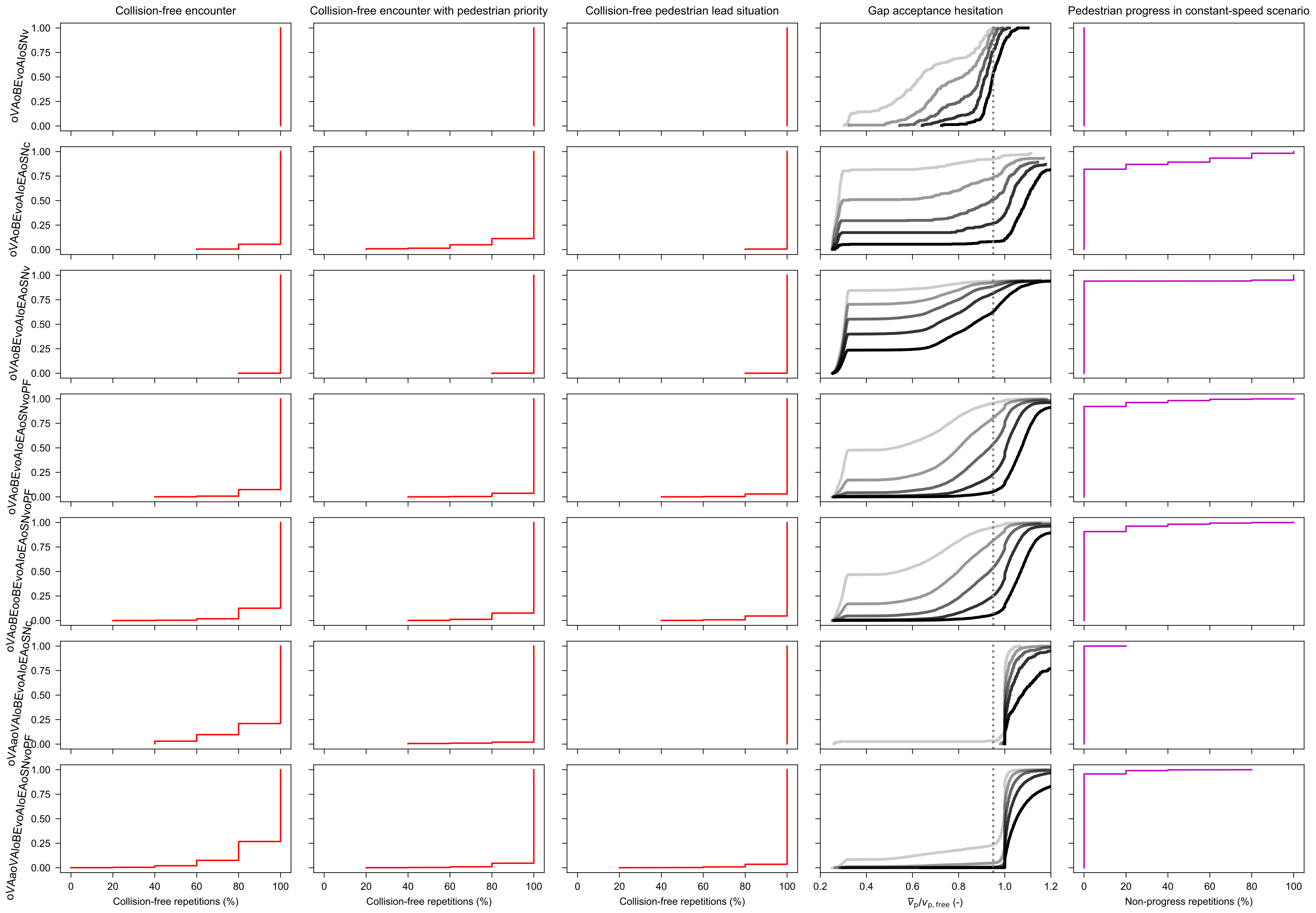

Figure S16: Cumulative distributions of quantitative metric values obtained across all parameterizations of a selected subset of the more complex stochastic model variants. See Fig. S13 for further explanations.

The fifth row shows that the further addition of `oBEo` did not alter the model behaviour notably, and the final two rows show that with `oVAaoVAIoBEvoAI` as the deterministic base instead of `oVAoBEvoAI`, the capacity for gap acceptance hesitation all but disappeared.

Fig. S17 shows parameterizations meeting all of the criteria in these tests, for the `oVAoBEvoAIoEAoSNvoPF` model variant. The distribution of these retained parameters (shown in green in the figure) can be compared to those tested (grey in the figure; sampled, as described above, from the parameterizations retained for the corresponding deterministic and simple stochastic model variants `oVAoBEvoAI` and `oVAoEAoSNvoPF`, as shown in Fig. S10 and Fig. S15, respectively). It can be seen that too small values of the sensory noise magnitude σ_v were rejected, but otherwise the fraction of parameterizations retained/rejected was relatively consistent across those originally sampled. As alluded to above, the rejection of parameterizations was mostly driven by the gap acceptance hesitation criterion, and the uniform pattern of rejection in Fig. S17 suggests that the typical rate of gap acceptance hesitation in this model is lower than the $4/5 = 80$ % used in the retention criterion. This is also discernible in Fig. 3E in the main paper, with the pedestrian model often choosing to increase speed instead. Indeed, this type of behaviour is observed also in human pedestrians [2, 43].

2.3 Tests on controlled experiment data

Fig. 4A in the main paper shows the empirically observed crossing initiation times in Experiment 1, as well as those obtained for the same scenarios with the `oVAoBEvoAIoEAoSNvoPF` model variant. Fig. S18 provides simulation results for a number of other models, showing that only model variants assuming both evidence accumulation and Bayesian perceptual filtering exhibited the empirically observed bimodal crossing pattern. It should be noted that there might very well exist parameterizations of the other model variants which can achieve

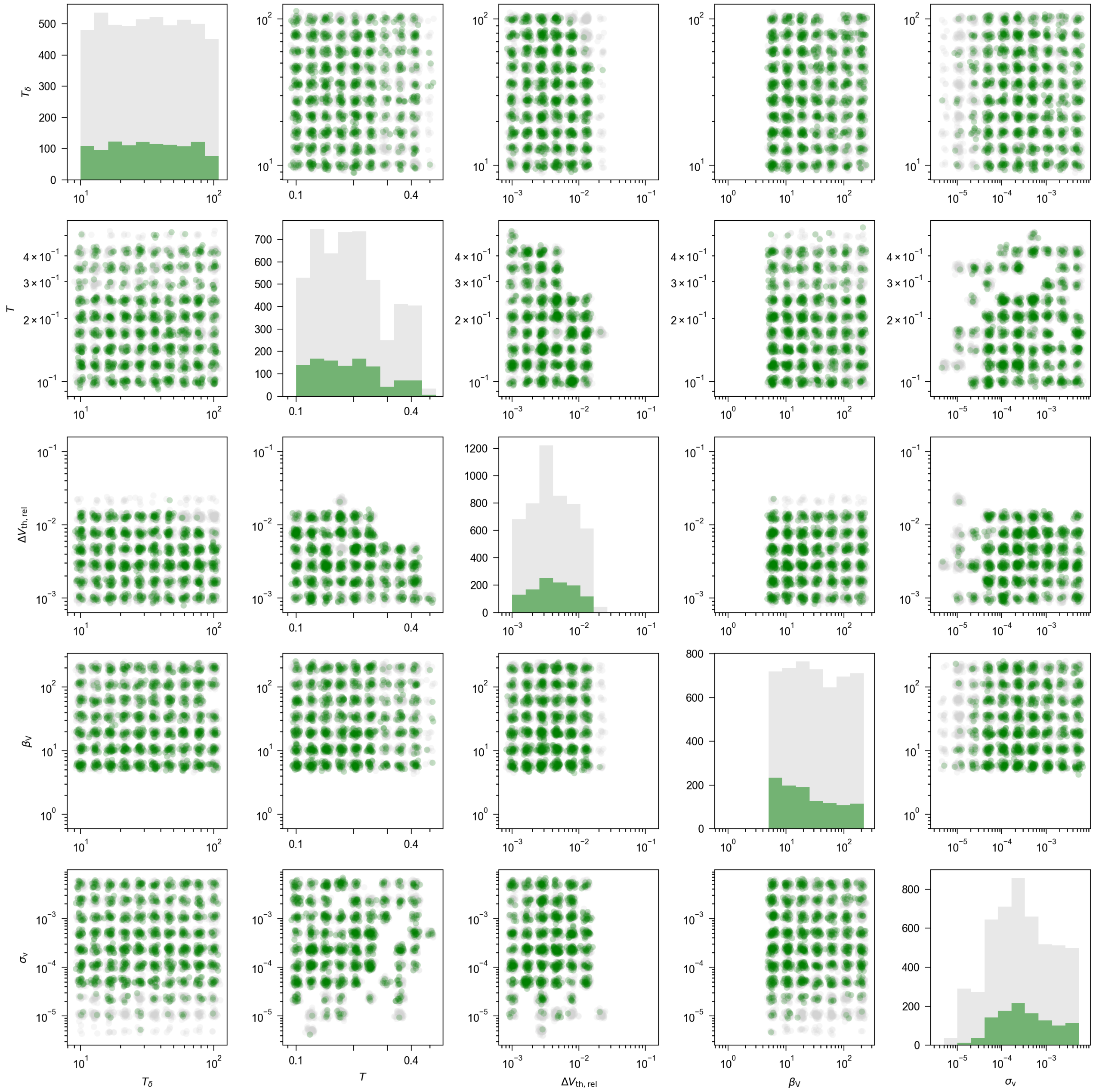

Figure S17: parameterizations sampled (light grey) and retained (green) in the tests of model variant `oVAoBEvoAIoEAoSNvoPF`. See Fig. S11 for a further explanation of how to read this figure.

bimodal crossing distributions. However, crucially, no such parameterizations were found among those which had been identified as achieving the other behavioural phenomena tested for in the deterministic and stochastic tests described above. In other words, only the model variants assuming both `oEA` and `oPF` were simultaneously capable of achieving both bimodal crossing distributions and the other targeted phenomena. The simplest model meeting these criteria was `oVAoBEvoAIoEAoSNvoPF`, which we therefore consider our maximally successful model variant.

Fig. S19 and Fig. S20 show, for model variant `oVAoBEvoAIoEAoSNvoPF`, which parameterizations were excluded due to non-progressing agent behaviour, in the model simulations for Experiment 1 and Experiment 2, respectively. For Experiment 1, it is clear that large sensory noise magnitudes σ_{v} was the main cause of non-progress; this is because with excessive noise a fully stopped vehicle near the pedestrian may on many individual time steps be perceived as already crossing in front of the pedestrian. Fig. S20 shows that this factor is also present in the fully interactive scenarios in Experiment 2, but here also the future reward devaluation time constant T_δ and the evidence accumulation threshold $\Delta V_{\mathrm{th,rel}}$ contribute. It can be seen in the top right (and bottom left) panel of Fig. S20 that there is a region of low σ_{v} and low T_δ, i.e., high aversion to time loss, where this type of non-progress did not occur at all in our simulations. The parameterizations with particularly frequent non-progress are all instead in a region of high T_δ and $\Delta V_{\mathrm{th,rel}}$ (top middle panel of Fig. S20). The reason for the latter is that the evidence accumulation scheme adopted here, which effectively low-pass filters the noisy action value estimates, can have the accumulative value estimate converge to a value below the decision threshold. Future work should investigate more generally formulated evidence accumulation schemes [16, 17, 50, 51].

References

[1] Jami Pekkanen, Oscar Terence Giles, Yee Mun Lee, Ruth Madigan, Tatsuru Daimon, Natasha Merat, and Gustav Markkula. Variable-Drift Diffusion Models of Pedestrian Road-Crossing Decisions. *Computational Brain & Behavior*, 5(1):60–80, 2022.

[2] Andrea Gorrini, Luca Crociani, Giuseppe Vizzari, and Stefania Bandini. Observation results on pedestrian-vehicle interactions at non-signalized intersections towards simulation. *Transportation Research Part F: Traffic Psychology and Behaviour*, 59:269–285, 2018.

[3] Rune Elvik. A review of game-theoretic models of road user behaviour. *Accident Analysis & Prevention*, 62:388–396, 2014.

[4] Wenxiang Chen, Xiangling Zhuang, Zixin Cui, and Guojie Ma. Drivers' recognition of pedestrian road-crossing intentions: Performance and process. *Transportation Research Part F: Traffic Psychology and Behaviour*, 64:552–564, 2019.

[5] András Várhelyi. Drivers' speed behaviour at a zebra crossing: a case study. *Accident Analysis & Prevention*, 30(6):731–743, 1998.

[6] Joshua Domeyer, Azadeh Dinparastdjadid, John D Lee, Grace Douglas, Areen Alsaid, and Morgan Price. Proxemics and kinesics in automated vehicle–pedestrian communication: Representing ethnographic observations. *Transportation Research Record*, 2673(10):70–81, 2019.

[7] Yee Mun Lee, Ruth Madigan, Oscar Giles, Laura Garach-Morcillo, Gustav Markkula, Charles Fox, Fanta Camara, Markus Rothmueller, Signe Alexandra Vendelbo-Larsen, Pernille Holm Rasmussen, Andre Dietrich, Dimitris Nathanael, Villy Portouli, Anna Schieben, and Natasha Merat. Road users rarely use explicit communication when interacting in today's traffic: implications for automated vehicles. *Cognition, Technology & Work*, 23(2):367–380, 2021.

[8] F. Camara, N. Bellotto, S. Cosar, F. Weber, D. Nathanael, M. Althoff, J. Wu, J. Ruenz, A. Dietrich, G. Markkula, A. Schieben, F. Tango, N. Merat, and C. Fox. Pedestrian Models for Autonomous Driving Part II: High-Level Models of Human Behavior. *IEEE Transactions on Intelligent Transportation Systems*, 22(9):5453–5471, 2020.

[9] Scott Ettinger, Shuyang Cheng, Benjamin Caine, Chenxi Liu, Hang Zhao, Sabeek Pradhan, Yuning Chai, Ben Sapp, Charles Qi, Yin Zhou, Zoey Yang, Aurélien Chouard, Pei Sun, Jiquan Ngiam, Vijay Vasudevan, Alexander McCauley, Jonathon Shlens, and Dragomir Anguelov. Large Scale Interactive Motion Forecasting for Autonomous Driving : The Waymo Open Motion Dataset. In *2021 IEEE/CVF International Conference on Computer Vision (ICCV)*, pages 9690–9699, October 2021. ISSN: 2380-7504.

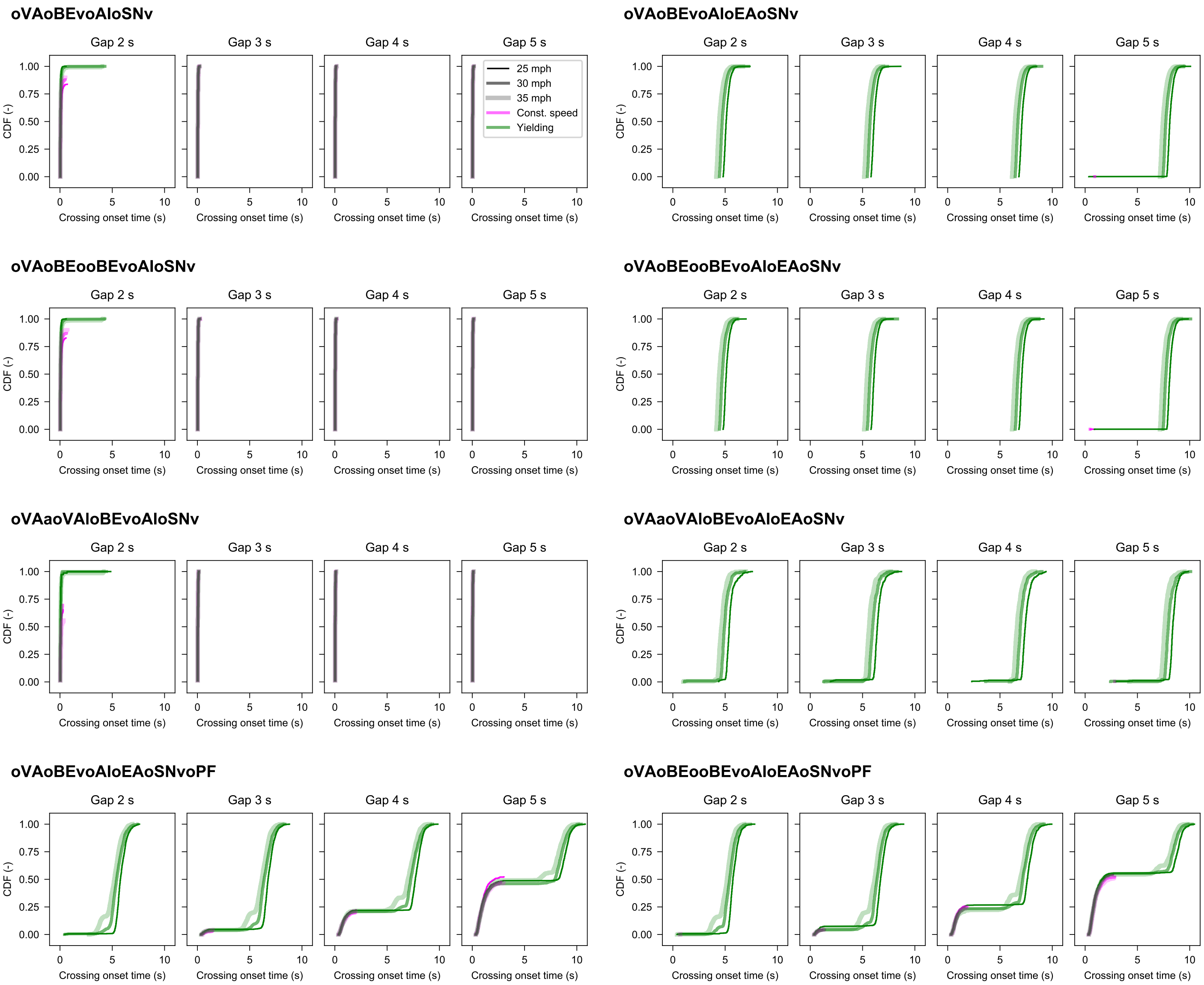

Figure S18: Predicted pedestrian crossing initiation times for the scenarios in Experiment 1, for a number of different model variants. Model variants without `oEA` (three top left examples) were faster than the human participants, almost always crossing before the car, whereas model variants with `oEA` but not `oPF` (three top right examples) made slower crossing decisions than the humans, almost always crossing after the car had passed. Only model variants combining `oEA` and `oPF` (bottom two examples) predicted the bimodal crossing pattern exhibited by the humans (Fig. 4A in the main paper).

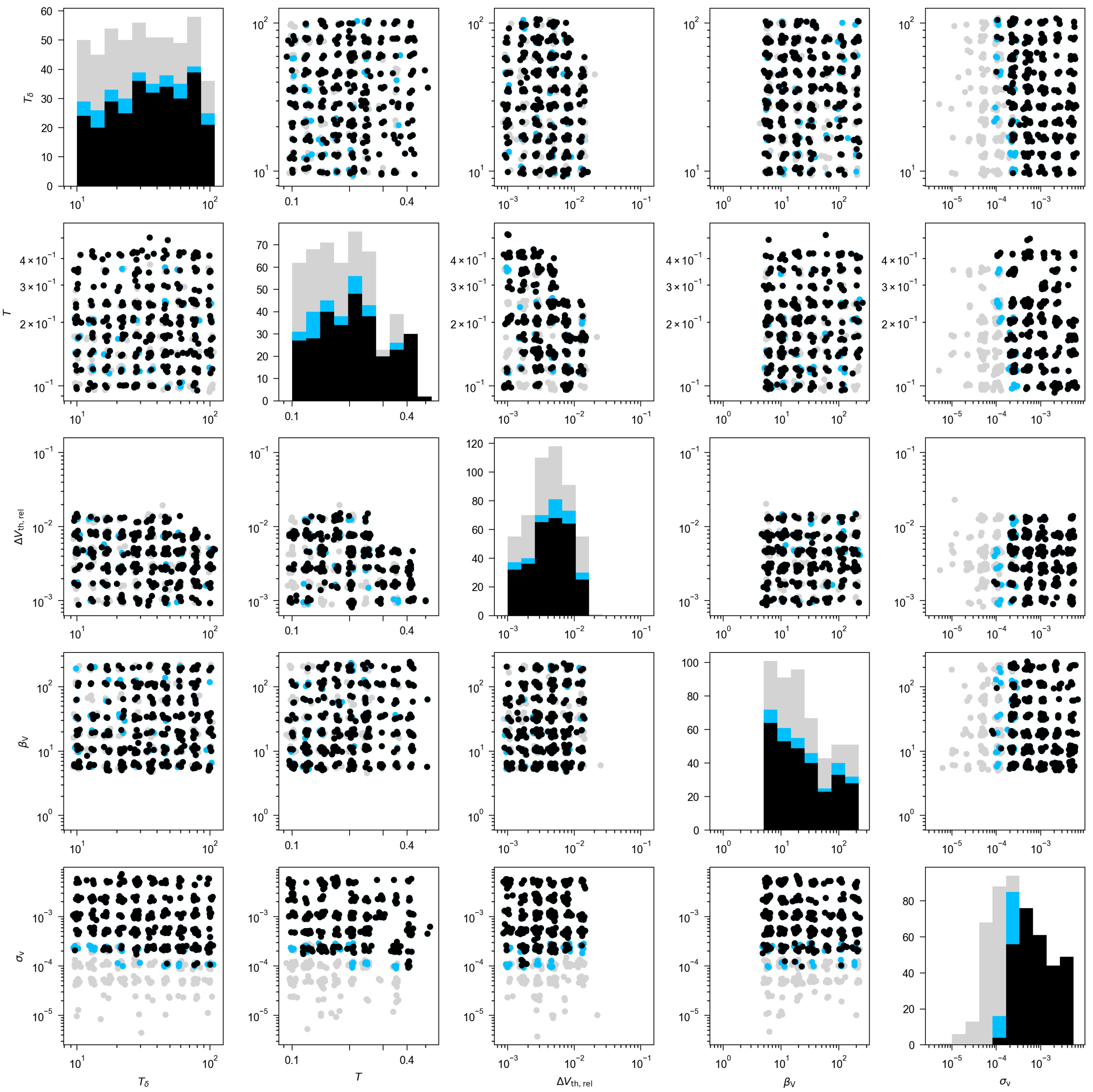

Figure S19: parameterizations sampled (shown in grey) when testing the `oVAoBEvoAIoEAoSNvoPF` model on the Experiment 1 scenarios, and the parameterizations which exhibited a non-progressing pedestrian in one or more simulations (shown in blue) and five or more simulations (shown in black).

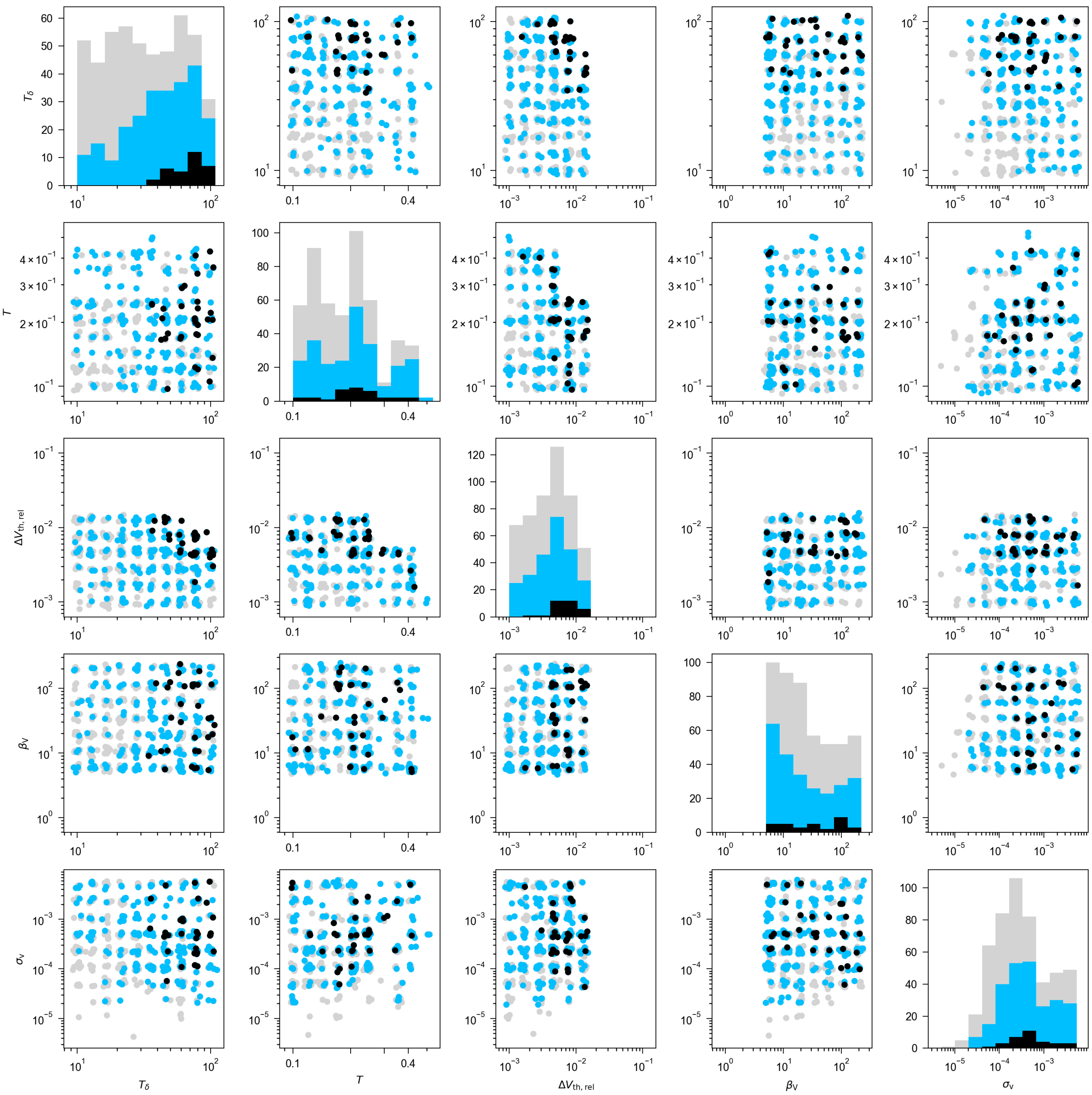

Figure S20: parameterizations sampled (shown in grey) when testing the `oVAoBEvoAIoEAoSNvoPF` model on the Experiment 2 scenarios, and the parameterizations which exhibited a non-progressing pedestrian or driver in one or more simulations (shown in blue) and five or more simulations (shown in black).

[10] Simon Suo, Sebastian Regalado, Sergio Casas, and Raquel Urtasun. TrafficSim: Learning to Simulate Realistic Multi-Agent Behaviors. In *Proceedings of the IEEE/CVF Conference on Computer Vision and Pattern Recognition (CVPR)*, pages 10400–10409, 2021.

[11] Shuo Feng, Xintao Yan, Haowei Sun, Yiheng Feng, and Henry X. Liu. Intelligent driving intelligence test for autonomous vehicles with naturalistic and adversarial environment. *Nature Communications*, 12(1):748, 2021.

[12] Dorsa Sadigh, Nick Landolfi, Shankar S. Sastry, Sanjit A. Seshia, and Anca D. Dragan. Planning for cars that coordinate with people: leveraging effects on human actions for planning and active information gathering over human internal state. *Autonomous Robots*, 42(7):1405–1426, 2018.

[13] Wilko Schwarting, Alyssa Pierson, Javier Alonso-Mora, Sertac Karaman, and Daniela Rus. Social behavior for autonomous vehicles. *Proceedings of the National Academy of Sciences*, 116(50):24972–24978, 2019.

[14] Joshua E. Domeyer, John D. Lee, Heishiro Toyoda, Bruce Mehler, and Bryan Reimer. Driver-Pedestrian Perceptual Models Demonstrate Coupling: Implications for Vehicle Automation. *IEEE Transactions on Human-Machine Systems*, pages 1–10, 2022.

[15] Manon Prédhumeau, Lyuba Mancheva, Julie Dugdale, and Anne Spalanzani. Agent-Based Modeling for Predicting Pedestrian Trajectories Around an Autonomous Vehicle. *Journal of Artificial Intelligence Research*, 73:1385–1433, 2022.

[16] Roger Ratcliff, Philip L. Smith, Scott D. Brown, and Gail McKoon. Diffusion Decision Model: Current Issues and History. *Trends in Cognitive Sciences*, 20(4):260–281, 2016.

[17] Jerome R. Busemeyer, Sebastian Gluth, Jörg Rieskamp, and Brandon M. Turner. Cognitive and Neural Bases of Multi-Attribute, Multi-Alternative, Value-based Decisions. *Trends in Cognitive Sciences*, 23(3):251–263, 2019.

[18] James R. Wright and Kevin Leyton-Brown. Predicting human behavior in unrepeated, simultaneous-move games. *Games and Economic Behavior*, 106:16–37, 2017.

[19] Chris L. Baker, Rebecca Saxe, and Joshua B. Tenenbaum. Action understanding as inverse planning. *Cognition*, 113(3):329–349, 2009.

[20] Giovanni Pezzulo, Francesco Donnarumma, and Haris Dindo. Human Sensorimotor Communication: A Theory of Signaling in Online Social Interactions. *PLoS ONE*, 8(11):e79876, 2013.

[21] Dino J Levy and Paul W Glimcher. The root of all value: a neural common currency for choice. *Current Opinion in Neurobiology*, 22(6):1027–1038, 2012.

[22] Julian Jara-Ettinger, Laura E. Schulz, and Joshua B. Tenenbaum. The Naïve Utility Calculus as a unified, quantitative framework for action understanding. *Cognitive Psychology*, 123:101334, 2020.

[23] David C. Knill and Alexandre Pouget. The Bayesian brain: the role of uncertainty in neural coding and computation. *Trends in Neurosciences*, 27(12):712–719, 2004.

[24] Oh-Sang Kwon, Duje Tadin, and David C. Knill. Unifying account of visual motion and position perception. *Proceedings of the National Academy of Sciences*, 112(26):8142–8147, June 2015.

[25] Alan Newell. You can't play 20 questions with nature and win. In W. G. Chase, editor, *Visual information processing*. Academic Press, New York, 1973.

[26] Donald J. Robinaugh, Jonas M. B. Haslbeck, Oisín Ryan, Eiko I. Fried, and Lourens J. Waldorp. Invisible Hands and Fine Calipers: A Call to Use Formal Theory as a Toolkit for Theory Construction. *Perspectives on Psychological Science*, 16(4):725–743, 2021.

[27] Mark A. Pitt, Woojae Kim, Daniel J. Navarro, and Jay I. Myung. Global model analysis by parameter space partitioning. *Psychological Review*, 113(1):57–83, 2006.

[28] Amir Rasouli, Iuliia Kotseruba, and John K. Tsotsos. Understanding Pedestrian Behavior in Complex Traffic Scenes. *IEEE Transactions on Intelligent Vehicles*, 3(1):61–70, 2018.

[29] Malte Risto, Colleen Emmenegger, Erik Vinkhuyzen, Melissa Cefkin, and Jim Hollan. Human-vehicle interfaces: The power of vehicle movement gestures in human road user coordination. In *Proceedings of the Ninth International Driving Symposium on Human Factors in Driver Assessment, Training and Vehicle Design*, pages 186–192, 2017.

[30] Evangelia Portouli, Dimitris Nathanael, and Nicolas Marmaras. Drivers' communicative interactions: on-road observations and modelling for integration in future automation systems. *Ergonomics*, 57(12):1795–1805, December 2014.

[31] Gustav Markkula, Ruth Madigan, Dimitris Nathanael, Evangelia Portouli, Yee Mun Lee, André Dietrich, Jac Billington, Anna Schieben, and Natasha Merat. Defining interactions: a conceptual framework for understanding interactive behaviour in human and automated road traffic. *Theoretical Issues in Ergonomics Science*, 21(6):728–752, 2020.

[32] Simon F Giszter. Motor primitives—new data and future questions. *Current Opinion in Neurobiology*, 33:156–165, 2015.

[33] Peter Gawthrop, Ian Loram, Martin Lakie, and Henrik Gollee. Intermittent control: a computational theory of human control. *Biological Cybernetics*, 104(1-2):31–51, 2011.

[34] Gustav Markkula, Erwin Boer, Richard Romano, and Natasha Merat. Sustained sensorimotor control as intermittent decisions about prediction errors: computational framework and application to ground vehicle steering. *Biological Cybernetics*, 112(3):181–207, 2018.

[35] Malin Svärd, Gustav Markkula, Jonas Bärgman, and Trent Victor. Computational modeling of driver pre-crash brake response, with and without off-road glances: Parameterization using real-world crashes and near-crashes. *Accident Analysis & Prevention*, 163:106433, 2021.

[36] A. Thorstensson and H. Roberthson. Adaptations to changing speed in human locomotion: speed of transition between walking and running. *Acta Physiologica Scandinavica*, 131(2):211–214, 1987. _eprint: https://onlinelibrary.wiley.com/doi/pdf/10.1111/j.1748-1716.1987.tb08228.x.

[37] K. Hase and R. B. Stein. Analysis of Rapid Stopping During Human Walking. *Journal of Neurophysiology*, 80(1):255–261, July 1998. Publisher: American Physiological Society.

[38] Th. Robin, G. Antonini, M. Bierlaire, and J. Cruz. Specification, estimation and validation of a pedestrian walking behavior model. *Transportation Research Part B: Methodological*, 43(1):36–56, 2009.

[39] Andrew Whiten and RW Byrne. *Natural theories of mind: Evolution, development and simulation of everyday mindreading.* B. Blackwell Oxford, UK, 1991.

[40] Richard S. Sutton and Andrew G. Barto. *Reinforcement Learning: An Introduction.* The MIT Press, Cambridge, MA, USA, 2nd edition, 2018.

[41] Giovanni Pezzulo and Paul Cisek. Navigating the Affordance Landscape: Feedback Control as a Process Model of Behavior and Cognition. *Trends in Cognitive Sciences*, 20(6):414–424, 2016.

[42] Brett R. Fajen. Perceptual learning and the visual control of braking. *Perception & Psychophysics*, 70(6):1117–1129, 2008.

[43] Jeannette Montufar, Jorge Arango, Michelle Porter, and Satoru Nakagawa. Pedestrians' Normal Walking Speed and Speed When Crossing a Street. *Transportation Research Record: Journal of the Transportation Research Board*, 2002(1):90–97, January 2007.

[44] Luca Crosato, Hubert P. H. Shum, Edmond S. L. Ho, and Chongfeng Wei. Interaction-aware Decision-making for Automated Vehicles using Social Value Orientation. *IEEE Transactions on Intelligent Vehicles*, pages 1–11, 2022. Conference Name: IEEE Transactions on Intelligent Vehicles.

[45] John E. Laird, Allen Newell, and Paul S. Rosenbloom. SOAR: An architecture for general intelligence. *Artificial Intelligence*, 33(1):1–64, 1987.

[46] John R. Anderson, Daniel Bothell, Michael D. Byrne, Scott Douglass, Christian Lebiere, and Yulin Qin. An Integrated Theory of the Mind. *Psychological Review*, 111(4):1036–1060, 2004.

[47] Dario D Salvucci. Modeling Driver Behavior in a Cognitive Architecture. *Human Factors*, 48(2):362–380, 2006.

[48] Dario D. Salvucci. Rapid prototyping and evaluation of in-vehicle interfaces. *ACM Transactions on Computer-Human Interaction*, 16(2):1–33, June 2009.

[49] Carsten K. W. de Dreu and Paul A. M. van Lange. The Impact of Social Value Orientations on Negotiator Cognition and Behavior. *Personality and Social Psychology Bulletin*, 21(11):1178–1188, November 1995. Publisher: SAGE Publications Inc.

[50] Marius Usher and James L McClelland. The time course of perceptual choice: the leaky, competing accumulator model. *Psychological Review*, 108(3):550–592, 2001.

[51] Luana F. Nunes and Kevin Gurney. Multi-alternative decision-making with non-stationary inputs. *Royal Society Open Science*, 3(8):160376, 2016.

[52] Peter Dayan and Kent C. Berridge. Model-based and model-free Pavlovian reward learning: Revaluation, revision, and revelation. *Cognitive, Affective, & Behavioral Neuroscience*, 14(2):473–492, 2014.

[53] Andrew D. Wilson and Sabrina Golonka. Embodied Cognition is Not What you Think it is. *Frontiers in Psychology*, 4, 2013.

[54] Benjamin Y. Hayden and Yael Niv. The case against economic values in the orbitofrontal cortex (or anywhere else in the brain). *Behavioral Neuroscience*, 135(2):192–201, 2021.

[55] Mahir Gulzar, Yar Muhammad, and Naveed Muhammad. A Survey on Motion Prediction of Pedestrians and Vehicles for Autonomous Driving. *IEEE Access*, 9:137957–137969, 2021. Conference Name: IEEE Access.

[56] Iyad Rahwan, Manuel Cebrian, Nick Obradovich, Josh Bongard, Jean-François Bonnefon, Cynthia Breazeal, Jacob W. Crandall, Nicholas A. Christakis, Iain D. Couzin, Matthew O. Jackson, Nicholas R. Jennings, Ece Kamar, Isabel M. Kloumann, Hugo Larochelle, David Lazer, Richard McElreath, Alan Mislove, David C. Parkes, Alex 'Sandy' Pentland, Margaret E. Roberts, Azim Shariff, Joshua B. Tenenbaum, and Michael Wellman. Machine behaviour. *Nature*, 568(7753):477–486, April 2019. Number: 7753 Publisher: Nature Publishing Group.

[57] Olger Siebinga, Arkady Zgonnikov, and David Abbink. A human factors approach to validating driver models for interaction-aware automated vehicles. *ACM Transactions on Human-Robot Interaction*, 2022.

[58] Aravinda Ramakrishnan Srinivasan, Yi-Shin Lin, Morris Antonello, Anthony Knittel, Mohamed Hasan, Majd Hawasly, John Redford, Subramanian Ramamoorthy, Matteo Leonetti, Jac Billington, Richard Romano, and Gustav Markkula. Beyond RMSE: Do machine-learned models of road user interaction produce human-like behavior?, June 2022. arXiv:2206.11110 [cs].

[59] David W. Franklin and Daniel M. Wolpert. Computational Mechanisms of Sensorimotor Control. *Neuron*, 72(3):425–442, November 2011.

[60] Hans Straka, John Simmers, and Boris P. Chagnaud. A New Perspective on Predictive Motor Signaling. *Current Biology*, 28(5):R232–R243, March 2018.

[61] Yi-Shin Lin, Aravinda Ramakrishnan Srinivasan, Matteo Leonetti, Jac Billington, and Gustav Markkula. A Utility Maximization Model of Pedestrian and Driver Interactions. *IEEE Access*, pages 1–1, 2022. Conference Name: IEEE Access.

[62] Meng Wang, Serge P. Hoogendoorn, Winnie Daamen, Bart van Arem, and Riender Happee. Game theoretic approach for predictive lane-changing and car-following control. *Transportation Research Part C: Emerging Technologies*, 58:73–92, 2015.

[63] Serge Hoogendoorn and Piet H. L. Bovy. Simulation of pedestrian flows by optimal control and differential games. *Optimal Control Applications and Methods*, 24(3):153–172, 2003.

[64] Kai Tian, Gustav Markkula, Chongfeng Wei, Yee Mun Lee, Ruth Madigan, Natasha Merat, and Richard Romano. Explaining unsafe pedestrian road crossing behaviours using a Psychophysics-based gap acceptance model. *Safety Science*, 154:105837, October 2022.

[65] Bingni W. Brunton, Matthew M. Botvinick, and Carlos D. Brody. Rats and Humans Can Optimally Accumulate Evidence for Decision-Making. *Science*, 340(6128):95–98, 2013.

[66] Teng Leng Ooi, Bing Wu, and Zijiang J. He. Distance determined by the angular declination below the horizon. *Nature*, 414(6860):197–200, 2001.

[67] Christopher R. Fetsch, Gregory C. DeAngelis, and Dora E. Angelaki. Bridging the gap between theories of sensory cue integration and the physiology of multisensory neurons. *Nature Reviews Neuroscience*, 14(6):429–442, 2013.

[68] Haris Dindo, Daniele Zambuto, and Giovanni Pezzulo. Motor Simulation via Coupled Internal Models Using Sequential Monte Carlo. In *Proceedings of IJCAI 2011*, pages 2113–2119, 2011.

[69] Yee Mun Lee, Ruth Madigan, Chinebuli Uzondu, Jorge Garcia, Richard Romano, Gustav Markkula, and Natasha Merat. Learning to interpret novel eHMI: The effect of vehicle kinematics and eHMI familiarity on pedestrian' crossing behavior. *Journal of Safety Research*, 80:270–280, 2022.

[70] Amir Hossein Kalantari, Yue Yang, Jorge Garcia de Pedro, Yee Mun Lee, Anthony Horrobin, Albert Solernou, Christopher Holmes, Natasha Merat, and Gustav Markkula. Who goes first? A distributed simulator study of vehicle-pedestrian interaction, August 2022.

[71] Ehsan Sadraei, Richard Romano, Natasha Merat, J Garcia de Pedro, Yee Mun Lee, Ruth Madigan, Chinebuli Uzondu, Wei Lyu, and Andrew Tomlinson. Vehicle-pedestrian interaction: A distributed simulation study. In *Proceedings of the Driving Simulation 2020 Europe Conference*, pages 147–1154, Antibes, France, 2020.

[72] Danielle J. Navarro. Between the Devil and the Deep Blue Sea: Tensions Between Scientific Judgement and Statistical Model Selection. *Computational Brain & Behavior*, 2(1):28–34, 2019.

[73] Seth Roberts and Harold Pashler. How persuasive is a good fit? A comment on theory testing. *Psychological review*, 107(2):358–367, 2000.

[74] Gustav Markkula. Modeling driver control behavior in both routine and near-accident driving. *Proceedings of the Human Factors and Ergonomics Society Annual Meeting*, 58(1):879–883, 2014.

[75] Florin Dzeladini, Jesse van den Kieboom, and Auke Ijspeert. The contribution of a central pattern generator in a reflex-based neuromuscular model. *Frontiers in Human Neuroscience*, 8, 2014.

[76] Hong Zhu, Abdulkarim Almukdad, Miho Iryo-Asano, Wael K.M. Alhajyaseen, Hideki Nakamura, and Xin Zhang. A novel agent-based framework for evaluating pedestrian safety at unsignalized mid-block crosswalks. *Accident Analysis & Prevention*, 159:106288, 2021.

[77] Thomas F Fugger, Bryan C Randles Jr, Anthony C Stein, William C Whiting, and Brian Gallagher. Analysis of pedestrian gait and perception-reaction at signal-controlled crosswalk intersections. *Transportation Research Record*, 1705(1):20–25, 2000.

[78] Jakub Zębala, Piotr Ciępka, and Adam Reza. Pedestrian acceleration and speeds. *Problems of Forensic Sciences*, 91:227–234, 2012.

[79] Jun Wang, Karen K. Dixon, Hainan Li, and Jennifer Ogle. Normal Acceleration Behavior of Passenger Vehicles Starting from Rest at All-Way Stop-Controlled Intersections. *Transportation Research Record*, 1883(1):158–166, 2004.

[80] P. S. Bokare and A. K. Maurya. Acceleration-Deceleration Behaviour of Various Vehicle Types. *Transportation Research Procedia*, 25:4733–4749, 2017.

[81] David N Lee. A theory of visual control of braking based on information about time-to-collision. *Perception*, 5(4):437–459, 1976.

[82] Qingwan Xue, Gustav Markkula, Xuedong Yan, and Natasha Merat. Using perceptual cues for brake response to a lead vehicle: Comparing threshold and accumulator models of visual looming. *Accident Analysis & Prevention*, 118:114–124, 2018.

[83] Jack M Loomis and Joshua M Knapp. Visual perception of egocentric distance in real and virtual environments. In Lawrence J Hettinger and Michael W Haas, editors, *Virtual and adaptive environments*, pages 21–46. Lawrence Erlbaum Associates, London, 2003.

[84] Russell Golman, Sudeep Bhatia, and Patrick Bodilly Kane. The dual accumulator model of strategic deliberation and decision making. *Psychological Review*, 127(4):477–504, 2020.

[85] Fatema T. Johora and Jörg P. Müller. Zone-Specific Interaction Modeling of Pedestrians and Cars in Shared Spaces. *Transportation Research Procedia*, 47:251–258, 2020.

[86] Tao Li, Suresh P. Sethi, ,Santa Clara University, 500 El Camino Real, Santa Clara, CA 95053, USA, and ,The University of Texas at Dallas, 800 W Campbell Rd, Richardson, TX 75080, USA. A review of dynamic Stackelberg game models. *Discrete & Continuous Dynamical Systems - B*, 22(1):125–159, 2017.

[87] Sebastian Bitzer, Hame Park, Felix Blankenburg, and Stefan J. Kiebel. Perceptual decision making: drift-diffusion model is equivalent to a Bayesian model. *Frontiers in Human Neuroscience*, 8, 2014.

[88] Gustav Markkula, Zeynep Uludağ, Richard McGilchrist Wilkie, and Jac Billington. Accumulation of continuously time-varying sensory evidence constrains neural and behavioral responses in human collision threat detection. *PLOS Computational Biology*, 17(7):e1009096, 2021.

[89] Gustav Markkula, Johan Engström, Johan Lodin, Jonas Bärgman, and Trent Victor. A farewell to brake reaction times? Kinematics-dependent brake response in naturalistic rear-end emergencies. *Accident Analysis & Prevention*, 95:209–226, 2016.
